# Supplementary material for: Effects of brief exposure to misinformation about e-cigarette harms on twitter: a randomised controlled experiment
Source: BMJ Open. 2021 Sep 1;11(9):e045445. doi: 10.1136/bmjopen-2020-045445 (PMC8413940; doi:10.1136/bmjopen-2020-045445)
Supplement: Supplementary data [file bmjopen-2020-045445supp001.pdf]

Supplementary material 1: additional questions about tweets

Questions asked after each tweet

We are interested in learning whether you would reply, retweet, like, and share this message, whether you use Twitter regularly or not. A reply is a response to another person's Tweet, a Retweet is a re-posting of a Tweet, Likes are used to show appreciation for a Tweet, and you can Share a tweet via direct message, text message, or email.

Please rate how much you disagree or agree with the following statements about the message you just saw.

a. PE1: This message is worth remembering.
b. PE2: This message grabbed my attention.
c. PE3: This message is powerful.
d. PE4: This message is informative.
e. PE5: This message is meaningful to me.
f. PE6: This message is convincing.

1. Strongly disagree (1)
2. Disagree (2)
3. Neither disagree nor agree (3)
4. Agree (4)
5. Strongly agree (5)

Intentions of replyiong/retweeting/liking/sharing Tweets

Are you likely to Reply to this message?
1. Yes
2. No

Are you likely Retweet this message?
1. Yes
2. No

Are you likely to Like this message?
1. Yes
2. No

Are you likely to Share this message?
1. Yes
2. No

Emotional responses

Please mark an answer for each question in the table below. When thinking about e-cigarettes, does the message you just saw make you feel…

a. Scared
b. Hopeful
c. Worried
d. Happy

e. Angry
f. Relieved

1. Not at all
2. A little
3. Some
4. A lot
5. Completely
