# Supplementary material for: Effects of brief exposure to misinformation about e-cigarette harms on twitter: a randomised controlled experiment
Source: BMJ Open. 2021 Sep 1;11(9):e045445. doi: 10.1136/bmjopen-2020-045445 (PMC8413940; doi:10.1136/bmjopen-2020-045445)
Supplement: Supplementary data [file bmjopen-2020-045445supp002.pdf]

Supplementary material 2: experimental conditions

Experimental condition	Exposure 1	Exposure 2	Exposure 3	Exposure 4
1: E-cigarettes are as or more harmful than regular cigarettes	Let's focus on the matter at hand which is big tobacco duping an entire generation – again – on our watch. Let's get the word out and educate young people and old, vaping and e-cigarettes kill.	Smoking takes decades to cause cancer. Vaping, it seems, takes only a few years. The evidence is clear enough for US to ban flavoured vaping today, yep, today. The rest will follow as facts emerge, I imagine. It's pretty disgusting anyway – try it.	Vaping is still pretty much as dangerous as cigs because everything goes directly into your lungs. Oh I forgot to mention the flavouring chemicals in vapes can also cause cancer. Seriously, look up actual medical research please.	Juul should be banned immediately. Anyone thinking vaping cigarettes is better than smoking is being conned. Vaping chemicals into your lungs will kill you. Altria is a murderer. Flat out mass murder.
2: E-cigarettes are completely harmless	Wow. You're a doctor and you are spreading this fearmonger propaganda? What happened to your oath do no harm? There are zero proven harms in the 15 years vaping has existed when used in the suggested parameters. I highly suggest you educate yourself on all the facts.	I'm an asthmatic lol. I know the science behind vaping. It's completely safe. Big tobacco scares ppl. Like truth dot .org…big tobacco supports them. It's crazy.	Oh, it's not only safer, they are safe – or, you know of any harm by vaping though ~15y on the market and ~50.000.000 users world-wide? – and ~8.000 flavours! No, didn't think so because none. That's how safe vaping is – did say vaping. Any objections to that?	I don't worry about the ingredients of e-juice for vaping, they are harmless, but I do wonder about the artificial breathing, the regular deep puffing. Do trumpet players get a breathing disorder? My puffing e-cigs is kind of like that.
3: Messages expressing uncertainty about e-cigarettes	We still don't know how safe vaping is – it's time to get more information about the risks of e-cigarettes.	And people are like "but it's not that bad because it's not smoke" ok but nicotine is harmful with or without smoke and there is very limited research done on e-cigs so the FDA doesn't know how harmful they actually are to the extent that	This whole anti-vaping schtick is cooked up by drug regulation & enforcement to make sure the money keeps flowing to their coffers. I have yet to see a single credible piece of evidence that vaping causes real harm.	Is San Francisco's vaping ban backed by science? San Francisco has decided to ban the sale of e-cigarettes in 2020, hoping to curb a surge in vaping among adolescents. But is the policy backed up by the available

		we know cigarettes are harmful.	(As in more harm than drinking too much coffee.)	evidence? How harmful is vaping?
4: Messages about physical activity (control condition)	Today reinforces my passion to push the need to exercise for not only the physical benefits. Get out and do something active for your mental health. Go for a walk and clear your mind. Find someone to join you and talk to them. My prayers go out to all today.	Adults (those aged 18 or older) need 150 minutes per week of moderate intensity physical activity to improve and maintain health.	It’s world mental health day and we know sport and physical activity can have powerful and positive effect on our wellbeing. That’s why we invest in projects that are changing lives.	Physical activity and exercise can have immediate and long-term health benefits. Most importantly, regular activity can improve our quality of life.
