# Supplementary material for: Effects of brief exposure to misinformation about e-cigarette harms on twitter: a randomised controlled experiment
Source: BMJ Open. 2021 Sep 1;11(9):e045445. doi: 10.1136/bmjopen-2020-045445 (PMC8413940; doi:10.1136/bmjopen-2020-045445)
Supplement: Author's manuscript [file bmjopen-2020-045445.draft_revisions.pdf]

BMJ Open is committed to open peer review. As part of this commitment we make the peer review history of every article we publish publicly available.

When an article is published we post the peer reviewers' comments and the authors' responses online. We also post the versions of the paper that were used during peer review. These are the versions that the peer review comments apply to.

The versions of the paper that follow are the versions that were submitted during the peer review process. They are not the versions of record or the final published versions. They should not be cited or distributed as the published version of this manuscript.

BMJ Open is an open access journal and the full, final, typeset and author-corrected version of record of the manuscript is available on our site with no access controls, subscription charges or pay-per-view fees (http://bmjopen.bmj.com).

If you have any questions on BMJ Open's open peer review process please email
info.bmjopen@bmj.com

BMJ Open

Effects of Brief Exposure to Misinformation about E-cigarette Harms on Twitter: A Randomised Controlled Experiment

Journal:	*BMJ Open*
Manuscript ID	bmjopen-2020-045445
Article Type:	Original research
Date Submitted by the Author:	02-Oct-2020

Complete List of Authors:	Wright, Caroline; University of Bristol, Bristol Medical School<br>Williams, Philippa; University of Bristol, Bristol Medical School<br>Elizarova, Olga; Play Collaborate Change<br>Dahne, Jennifer; Medical University of South Carolina College of Medicine, Psychiatry and Behavioral Sciences<br>Bian, Jiang; University of Florida<br>Zhao, Yunpeng; University of Florida, Health Outcomes & Biomedical Informatics, College of Medicine<br>Tan, Andy; University of Pennsylvania, Annenberg School for CommunicationU

Keywords:	PREVENTIVE MEDICINE, PUBLIC HEALTH, SOCIAL MEDICINE

SCHOLARONE™
Manuscripts

I, the Submitting Author has the right to grant and does grant on behalf of all authors of the Work (as defined in the below author licence), an exclusive licence and/or a non-exclusive licence for contributions from authors who are: i) UK Crown employees; ii) where BMJ has agreed a CC-BY licence shall apply, and/or iii) in accordance with the terms applicable for US Federal Government officers or employees acting as part of their official duties; on a worldwide, perpetual, irrevocable, royalty-free basis to BMJ Publishing Group Ltd ("BMJ") its licensees and where the relevant Journal is co-owned by BMJ to the co-owners of the Journal, to publish the Work in this journal and any other BMJ products and to exploit all rights, as set out in our licence.

The Submitting Author accepts and understands that any supply made under these terms is made by BMJ to the Submitting Author unless you are acting as an employee on behalf of your employer or a postgraduate student of an affiliated institution which is paying any applicable article publishing charge ("APC") for Open Access articles. Where the Submitting Author wishes to make the Work available on an Open Access basis (and intends to pay the relevant APC), the terms of reuse of such Open Access shall be governed by a Creative Commons licence – details of these licences and which Creative Commons licence will apply to this Work are set out in our licence referred to above.

Other than as permitted in any relevant BMJ Author's Self Archiving Policies, I confirm this Work has not been accepted for publication elsewhere, is not being considered for publication elsewhere and does not duplicate material already published. I confirm all authors consent to publication of this Work and authorise the granting of this licence.

For peer review only

Effects of Brief Exposure to Misinformation about E-cigarette Harms on Twitter: A Randomised Controlled Experiment

Authors and affiliations:

Caroline Wright PhD[1]*, Philippa Williams MSc[1]*, Olga Elizarova MPH[2], Jennifer Dahne PhD[3], Jiang Bian PhD[4], Yunpeng Zhao MSc[4] and Andy SL Tan PhD[5]

[1] Population Health Sciences, Bristol Medical School, University of Bristol, Bristol, UK.
[2] Play Collaborate Change, Boston, USA
[3] Psychiatry and Behavioral Sciences, College of Medicine, Medical University of South Carolina
[4] Health Outcomes & Biomedical Informatics, College of Medicine, University of Florida
[5] Annenberg School for Communication, University of Pennsylvania

* Joint first authors

Corresponding author:

Caroline Wright, Population Health Sciences, Bristol Medical School, University of Bristol, BF4, Barley House, Oakfield Grove, Bristol, BS8 2BN caroline.wright@bristol.ac.uk +44(0)117 3314011

Word count (main text): 3,400
Word count (abstract): 300
References: 31
Figures: 5
Tables: 5
Supplemental figures: 0

Back to top

For peer review only

Abstract

Objectives: To assess the effect of exposure to misinformation about e-cigarette harms on Twitter on adult current smokers' intention to quit smoking cigarettes, intention to purchase e-cigarettes and perceived relative harm of e-cigarettes compared to regular cigarettes.

Setting: An online randomized controlled experiment conducted in November 2019 among United States (US) and United Kingdom (UK) current smokers.

Participants: 2,400 adult current smokers aged ≥18 years who were not current e-cigarette users recruited from an online panel. Participants' were randomized in a 1:1:1:1 ratio using a least-fill randomizer function.

Interventions: Viewing 4 tweets in random order within one of four conditions: 1) e-cigarettes are just as or more harmful than smoking, 2) e-cigarettes are completely harmless, 3) e-cigarette harms are uncertain, and 4) a control condition of tweets about physical activity.

Primary outcomes measures: Self-reported post-test intention to quit smoking cigarettes, intention to purchase e-cigarettes, and perceived relative harm of e-cigarettes compared with smoking.

Results: Among US and UK participants, after controlling for baseline measures of the outcome, exposure to tweets that e-cigarettes are as more harmful than smoking versus control was associated with lower post-test intention to purchase e-cigarettes (β=-0.339,95%CI:-0.487,-0.191,p<0.001) and increased post-test perceived relative harm of e-cigarettes (β=0.341,95%CI:0.273,0.410,p<0.001). Among US smokers, exposure to tweets that e-cigarettes are completely harmless was associated with higher post-test intention to purchase e-cigarettes (β=0.229,95%CI:0.002,0.456,p=0.048) and lower post-test perceived relative harm of e-cigarettes (β=-0.154,95%CI:-0.258,0.050,p=0.004).

Conclusions: US and UK adult current smokers may be deterred from considering using e-cigarettes after brief exposure to tweets that e-cigarettes were just as or more harmful than smoking. Conversely, US adult current smokers may be encouraged to use e-cigarettes after exposure to tweets that e-cigarettes are completely harmless. These findings suggest that misinformation about e-cigarette harms may influence some adult smokers' decisions to consider using e-cigarettes.

Trial registration: ISRCTN registry: ISRCTN16082420.

For peer review only

Strengths and limitations of this study

- This is the first study to explore the effect of exposure to misinformation about e-cigarette harms on Twitter, showing that misinformation about e-cigarettes may be hindering efforts to reduce the burden of tobacco smoking on current smokers in the US and UK.
- We used a randomised controlled experimental design, which means we are unlikely to suffer from problems like confounding.
- We excluded visual content from our exposures and focused on Twitter: more research could be done to explore the impact of these factors.
- Our study sample did not fully represent the populations they were drawn from, which may mean our findings are not generalisable.

For peer review only

INTRODUCTION

Although e-cigarette use is not completely harmless,(1,2) there is a general agreement that the short-term health risks are considerably lower than smoking regular cigarettes.(2) Despite this growing consensus, several recent studies show misperceptions about the relative harms of e-cigarettes among current smokers are increasing in both the United States (US) and the United Kingdom (UK).(3–5) Between 2014-2019, the percentage of current adult smokers in the UK who thought e-cigarettes were less harmful than cigarettes decreased from 45% to 34% and an even lower proportions of people believe so among smokers who were not using e-cigarettes.(5) Many smokers also do not think that complete replacement of cigarettes with e-cigarettes would lead to major health benefits.(2) The US has a similar trend, with the percentage of adults perceiving e-cigarettes as less harmful than cigarettes declining from 29.3% to 25.8% between 2017-2018. Over the same period there was an increase from 1.8% to 4.4% of US adult smokers perceiving e-cigarettes as much more harmful than cigarettes.(4) The increasing trends of misperceptions about the relative harms of e-cigarettes compared with regular cigarettes are important for public health because perceived harms of e-cigarettes are associated with smokers' willingness to use e-cigarettes (6) as a harm reduction strategy.

Misperceptions of e-cigarette harms may be related to exposure to misinformation —information that is incorrect or misleading.(7) Based on the state of the science of e-cigarette harms,(1,2) misinformation related to e-cigarette harms was classified as the statements that either claim that e-cigarettes are equally or more harmful than smoking regular cigarettes or are completely harmless. As the evidence-base on e-cigarette harms has developed, related media and public discussion has involved uncertainty, defined as existing "when details of the situation are ambiguous, complex, unpredictable, or probabilistic; when information is unavailable or

For peer review only - http://bmjopen.bmj.com/site/about/guidelines.xhtml

inconsistent".(8) Therefore, the impact of exposure to statements that claim the evidence of e-cigarette harms are uncertain are also important.

Health information is commonly accessed online, with 63% of UK adults using the internet to look for health-related information,(9) and 75% of US adults using the internet as their first source of health information.(10) People are increasingly encountering health information through social media platforms such as Twitter or Facebook. (11) These platforms enable users to generate and share content (12) and contrary to other media, there is often limited verification of accuracy of health information.(13,14) A systematic review found user generated content was often inconsistent with clinical guidelines and health misinformation was increasingly available online.(15) We therefore focused on misinformation of e-cigarettes occurring on social media.

This study comprised US and UK participants as the contrasting policy approaches toward e-cigarette use across the two countries may mean that US and UK participants view harms associated with e-cigarettes differently. While the US approach focuses on protecting non-smokers from uptake of smoking via e-cigarette use, the UK's approach emphasises e-cigarettes as a harm reduction strategy to reduce the burden of risk on current smokers.(16) Further, the UK also has much stricter regulations relating to e-cigarette advertising and nicotine content of e-liquids compared with the US.(5)

To date, most studies have focused on health misinformation in relation to communicable diseases (7) and there is limited research on misinformation related to tobacco product use including e-cigarettes. While existing studies examined current perceptions of e-cigarette harms, little is known about the role of exposure to misinformation on social media on these perceptions, and consequently on e-cigarette intentions and use.(17) To address this research gap, we conducted a web-based randomised controlled experiment to assess the effect of

For peer review only

exposure to misinformation about e-cigarette harm on Twitter, on smokers' intentions to quit smoking, intentions to purchase e-cigarettes, and perceptions of the relative harm of e-cigarettes compared to regular cigarettes.

For peer review only

METHODS

Study design

We used a randomised controlled experimental design.(18) The study was conducted using the online consumer research panel, Prodege which recruited participants from the US and the UK via internet sources (i.e., email invitations, telephone alerts, banners and messaging on web sites and online communities). Participants' received reward points as per Prodege policies. The University of Bristol's Institutional Review Board approved this study.

Participants

Study participants were 2,400 self-reported adult smokers aged ≥18 years, who were not currently using e-cigarettes. Informed consent was obtained electronically through the survey platform.

Randomisation and masking

Following eligibility screening and having provided informed consent, participants completed baseline measures of study outcomes. Participants were then randomised to one of four experimental conditions: 1) E-cigarettes are as or more harmful, 2) E-cigarettes are completely harmless, and 3) Uncertain message about e-cigarettes. Tweets for the control condition comprised four tweets about physical activity from Twitter. Participants were randomised in a 1:1:1:1 ratio using the in-built least-fill randomiser function on the Prodege survey platform. Randomisation ensures that all participants have an equal chance of being assigned to each of the exposure conditions, and as such eliminates selection bias and associated problems with confounding. Adjusting for covariates is thus not needed in subsequent analysis.

Procedures

Participants were told they would be shown different types of health-related information and

For peer review only

asked for their opinions about e-cigarettes. After randomisation to a condition, they viewed one tweet at a time in random order (four tweets in total) and were asked brief questions about each tweet (perceived effectiveness, liking and sharing, and emotions). Next, they completed post-test measures of the study outcomes, current tobacco use behaviours, health information exposure, media use, and sociodemographic and psychological characteristics.

We captured tweets about e-cigarette harms using a validated machine learning algorithm the study team developed in an earlier phase of this research.(19,20) Using the random sample function within SPSS we selected a random 1% sample (n=499) of these tweets. Next the study team narrowed this sample of tweets to approximately 20 tweets per experimental condition and discussed whether each tweet would be suitable as experimental stimuli based on the following criteria: 1) explicit statement that e-cigarettes were either as or more harmful than smoking, completely harmless, or uncertain; 2) no mention of children or young people: 3) no mention of specific diseases; 4) no profanities; 5) had multiple 'likes' or 'retweets'; 6) no advertising; 7) no pictures; and 8) was available publicly (i.e., not deleted).

We selected four representative tweets for each of the three experimental conditions: 1) E-cigarettes are as or more harmful, 2) E-cigarettes are completely harmless, and 3) Uncertain message about e-cigarettes. Tweets for the control condition comprised four tweets about physical activity from Twitter. We selected physical activity promotion messages as the control condition to reduce potential bias due to experimenter demand and avoided topics related to e-cigarettes such as other forms of tobacco, alcohol or substance use behaviours. Figures 1-4 display the tweets that comprised each message condition.

Outcome measures

Baseline and post-test intention to quit smoking

For peer review only

Participants were asked to consider a smoking cessation contemplation ladder.(21) They were asked: “You have told us that you are currently smoking cigarettes. Each number below represents where various smokers are in their thinking about quitting. Please enter a number that indicates where you are now, ranging from “No thought of quitting” (0) to “Taking action to quit (e.g., cutting down, enrolling in a program)” (10).

Baseline and post-test intention to purchase e-cigarettes

Participants were asked: "How probable is it that you will purchase e-cigarettes in the next month?" Answer options ranged from “No chance, almost no chance” (0) to “Certain, practically certain” (10).(22)

Baseline and post-test perceived relative harm of e-cigarettes compared to regular cigarettes

Participants were asked: “Compared to smoking cigarettes, would you say that electronic cigarettes are” Much less harmful (1) to much more harmful (5). This question included the option of don’t know.(23) Two hundred and thirty-three participants answered ‘don’t know’ to this question either at baseline or post-exposure and as such were not included in the analysis.

Demographic and health information.

Participants were asked to provide sociodemographic information including age, sex, race, highest education level, number of days of cigarette smoking in the past 30 days, ever use of e-cigarettes, information search about e-cigarettes, and social media use (see Table 1).

For peer review only

Table 1: Sociodemographic characteristics of study sample by experimental condition and country

	US				UK			
**Condition**	1	2	3	4	1	2	3	4
**Characteristics**	n=300	n=300	n=300	n=300	n=300	n=300	n=300	n=300
**Age: Mean (SD)**								
	50.5 (13.6)	50.0 (13.6)	50.0 (14.7)	50.3 (13.5)	44.1 (14.6)	44.2 (14.4)	44.0 (14.8)	42.8 (14.6)
**Sex: No. (%)**								
Female	153 (51.0)	154 (51.3)	154 (51.3)	140 (46.7)	126 (42.0)	136 (45.3)	125 (41.7)	135 (45.0)
**US Race: No. (%)**								
White	206 (68.7)	214 (71.3)	211 (70.3)	220 (73.3)				
Black or African American	51 (17.0)	47 (15.7)	52 (17.3)	51 (17.0)				
Other ethnicity	43 (14.3)	39 (13.0)	37 (12.3)	29 (9.7)				
**UK Ethnicity: No. (%)**								
White					284 (94.7)	276 (92.0)	278 (92.7)	282 (94.0)
Other ethnicity					16 (5.3)	24 (8.0)	22 (7.3)	18 (6.0)
**Education: No. (%)**								
High/Secondary school or below	83 (27.7)	99 (33.0)	91 (30.3)	89 (29.7)	118 (39.3)	126 (42.0)	122 (40.7)	129 (43.0)
Some college/ further education college	111 (37.0)	122 (40.7)	123 (41.0)	110 (36.7)	110 (36.7)	103 (34.3)	105 (35.0)	105 (35.0)
College/ University degree or higher	106 (35.3)	79 (26.3)	86 (28.7)	101 (33.7)	72 (24.0)	71 (23.7)	73 (24.3)	66 (22.0)
**Smoking status: Mean (SD)**								
No. days smoked in last 30 days	28.9 (4.2)	27.8 (5.9)	27.7 (5.9)	28.1 (5.4)	27.5 (6.3)	27.4 (6.9)	26.7 (7.7)	27.1 (7.00)
**E-cigarette use: No. (%)**								
Never used e-cigarettes	145 (48.3)	144 (48.0)	152 (50.7)	158 (52.7)	138 (46.0)	124 (41.3)	152 (50.7)	148 (49.3)
**Have you ever looked for e-cigarette information: No. (%)**								
Yes	75 (25.0)	81 (27.0)	58 (19.3)	76 (25.3)	72 (24.0)	78 (26.0)	74 (24.7)	82 (27.3)
**Frequency of hearing e-cigarettes harmful: No. (%)**								
Not at all	22 (7.3)	37 (12.3)	38 (12.7)	16 (5.3)	42 (14.0)	54 (18.0)	64 (21.3)	45 (15.0)
A little	68 (22.7)	78 (26.0)	83 (27.7)	67 (22.3)	123 (41.0)	113 (37.7)	131 (43.7)	122 (40.7)
Some	105 (35.0)	102 (34.0)	103 (34.3)	96 (32.0)	81 (27.0)	90 (30.0)	69 (23.0)	75 (25.0)
A lot	105 (35.0)	83 (27.7)	76 (25.3)	121 (40.3)	54 (18.0)	43 (14.3)	36 (12.0)	58 (19.3)
**Frequency of hearing e-cigarettes harmless: No. (%)**								
Not at all	132 (44.0)	107 (35.7)	150 (50.0)	137 (45.7)	122 (40.7)	97 (32.3)	115 (38.3)	145 (48.3)
A little	86 (28.7)	100 (33.3)	76 (25.3)	75 (25.0)	97 (32.3)	104 (34.7)	110 (36.7)	85 (28.3)
Some	56 (18.7)	61 (20.3)	55 (18.3)	61 (20.3)	53 (17.7)	68 (22.7)	53 (17.7)	58 (19.3)
A lot	26 (8.7)	32 (10.7)	19 (6.3)	27 (9.0)	28 (9.3)	31 (10.3)	22 (7.3)	12 (4.0)

Notes. Test for variance across conditions; continuous variables analysed using one-way Anova test, categorical variables analysed using χ^2 test

For peer review only

Statistical Analysis

We used GPower (version 3.1) (24) to estimate effect sizes in the outcome variables as a function of message condition, assuming two-tailed tests, with 80% power and $\alpha=0.05$. Based on these analyses, a final sample size of 2,400 (600 in each arm) was deemed sufficient power to detect small effects in between-subject analyses of the main effect of condition among adult smokers (f=0.07). In stratified analyses by country, a sample size of 1,200 (300 in each arm) will also ensure sufficient power to detect small effects between conditions (f=0.10).

Analyses were completed in 2020. We performed univariate analyses for all study variables. Next, we analysed whether participants across conditions differed in terms of individual characteristics. To address the study hypothesis, we utilised linear regression to predict post-test intentions to quit smoking, intentions to purchase e-cigarettes, and perceived relative harm of e-cigarettes by experimental condition compared with the control condition, adjusting for baseline measures of each outcome respectively. Owing to overdispersion of the second outcome measure, intentions to purchase e-cigarettes, we additionally ran negative binomial regression models. We also ran sensitivity analyses, including country as a covariate (owing to the differences in baseline measurements between the US and the UK; analysis using robust standard errors and bootstrapping – owing to non-normal distribution of residuals. We further conducted stratified analyses to compare the effects of experimental condition on each study outcome among US and UK participants separately. Stata version 15.1 was used to conduct all analyses.(25)

For peer review only

RESULTS

Participants were 2,400 adult current smokers recruited between 8-28 November 2019 (see Figure 5: CONSORT diagram). They were aged 18-84 years (mean=47.0, SD=14.58), 46.8% were female, 70.9% of the US participants were White, 16.8% Black or African American and 12.3% were of Other racial background. While 93.3% of the UK cohort were White and 6.7% were from other ethnic backgrounds. Most of the cohort (82.1%) smoked cigarettes every day and 51.6% had ever used e-cigarettes. Table 1 summarises the sample characteristics by experimental condition. We found that randomisation had been achieved and all covariates were distributed evenly across the four study conditions.

Three quarters of participants (n=1,804, 75.2%) had not previously searched for information on e-cigarettes. Participants were more likely to report that they had heard that e-cigarettes are more harmful than cigarettes (n=1,297, 54.0%), than hearing that e-cigarettes are harmless (n=662, 27.6%). Over half of the sample (n=1,426, 59.4%) had never used Twitter, with Facebook being the most common social media platform used several times a day (n=1,194, 49.8%).

At baseline, 25.2% of participants (n= 605) placed themselves in the middle of the intention to quit ladder (mean=5.0, SD=3.0), this was similar for both US and UK participants. Over half the participants (n=1,312, 54.7%) said that there was no chance/almost no chance that they would buy e-cigarettes in the next month. The distributions for intentions to buy, were also very similar for US and UK participants. However, there were marked differences between the two populations with respect to perceptions of the relative harm of e-cigarettes: nearly twice as many UK participants said that e-cigarettes are much less harmful than regular cigarettes compared to US participants. Similarly, more than twice as many UK participants said that e-cigarettes are less harmful than regular cigarettes (n=448, 37.3%), compared to US participants (n=222,

For peer review only

18.5%). Conversely, more than three times as many US participants thought that e-cigarettes are much more harmful than regular cigarettes (US: n=217, 18.1%, UK: n=69, 5.8%) and more than twice as many saw them as more harmful (US: n=128, 10.7%, UK: n=62, 5.2%).

We additionally compared the mean and standard deviation (SD) for the outcome measures, both pre- and post-exposure across the four conditions for the US and UK separately (Table 2). We found that pre-exposure intentions to quit and perceptions of the relative harm of e-cigarettes were generally higher and intentions to purchase e-cigarettes were generally lower among US participants.

Tables 3 and 4 summarise the results from the regression analyses. The adjusted analysis includes both the experimental condition as the exposure and the baseline measure of the outcomes. We present the adjusted analysis here. Compared with the control condition, there was no difference in the post-test intention to quit smoking among those who viewed tweets stating that e-cigarettes are as or more harmful than cigarettes, the completely harmless condition or tweets that are uncertain. The results did not change substantially in the stratified analysis (Table 4).

Compared with participants assigned to the control group, there was a statistically significant reduction in post-test intention to purchase e-cigarettes for those exposed to the as or more harmful messages (β= -0.339, 95%CI: -0.487, -0.191, $p<0.001$). In the stratified analysis, the effect of viewing as or more harmful tweets on reducing intentions to purchase e-cigarettes was observed in both US (β=-0.312, 95%CI: -0.522, -0.073, $p=0.011$) and UK samples (β= -0.365, 95%CI: -0.551, -0.178, $p<0.001$). Further, the effect of viewing tweets that e-cigarettes are completely harmless was associated with an increase in intention to purchase e-cigarettes but

For peer review only

Table 2: Outcome measures by experimental condition and country

	US				UK			
**Condition**	1	2	3	4	1	2	3	4
**Outcome measures**								
**Intentions to quit smoking**	n=300	n=300	n=300	n=300	n=300	n=300	n=300	n=300
Pre-exposure: Mean (SD)	5.16 (2.94)	5.25 (3.17)	5.23 (3.00)	5.48 (3.14)	4.72 (2.85)	4.73 (2.86)	4.83 (2.90)	4.78 (3.04)
Post-exposure: Mean (SD)	5.29 (2.96)	5.34 (3.15)	5.46 (3.04)	5.72 (3.20)	4.93 (2.90)	4.80 (2.91)	4.96 (2.89)	4.93 (3.09)
**Intentions to purchase e-cigarettes**	n=300	n=300	n=300	n=300	n=300	n=300	n=300	n=300
Pre-exposure: Mean (SD)	1.33 (2.24)	1.15 (2.08)	1.25 (2.20)	1.29 (2.23)	1.67 (2.37)	1.57 (2.33)	1.88 (2.54)	1.71 (2.47)
Post-exposure: Mean (SD)	0.98 (2.02)	1.30 (2.27)	1.16 (2.17)	1.27 (2.31)	1.21 (2.16)	1.68 (2.56)	1.73 (2.50)	1.79 (2.61)
**Perceptions of relative harms of e-cigarettes**	n=274	n=268	n=274	n=276	n=272	n=270	n=262	n=271
Pre-exposure: Mean (SD)	3.17 (1.03)	3.35 (1.28)	3.20 (1.04)	3.26 (1.10)	2.64 (0.95)	2.67 (0.93)	2.60 (0.90)	2.68 (0.90)
Post-exposure: Mean (SD)	3.45 (1.06)	3.15 (1.12)	3.22 (1.02)	3.22 (1.07)	3.02 (1.00)	2.60 (0.98)	2.60 (0.93)	2.66 (0.92)

For peer review only

Table 3: Adjusted regression analysis predicting intention to quit regular cigarettes, intention to purchase an e-cigarette and perceived relative harm of e-cigarettes compared to regular cigarettes

	Intention to quit smoking regular cigarettes (n=2,400)			Intention to purchase e-cigarette[1] (n=2,400)			Perceived relative harm of e-cigarettes compared to regular cigarettes (n=2,167)		
	β	95% CI	p	β	95% CI	p	β	95% CI	p
Control (referent)									
As or more harmful	-0.031	[-0.152, 0.091]	0.622	-0.339	[-0.487, -0.191]	≤0.001	0.341	[0.273, 0.410]	≤0.001
Completely harmless	-0.120	[-0.241, 0.002]	0.054	0.111	[-0.029, 0.250]	0.120	-0.106	[-0.174, -0.037]	0.003
Uncertainty	-0.017	[-0.139, 0.104]	0.780	-0.106	[-0.247, 0.036]	0.143	-0.018	[-0.051, 0.086]	0.615
		R^2= 0.874			*Pseudo R^2 = 0.2125* *Alpha=0: p*≤0.001			*R^2 =0.704*	

1. For intention to purchase e-cigarettes Negative Binomial Regression was conducted due to a zero-inflated distribution / non-normal distribution

For peer review only

Table 4: Adjusted regression analysis predicting intention to quit regular cigarettes, intention to purchase an e-cigarette and perceived relative harm of e-cigarettes compared to regular cigarettes stratified by country of residence status

	Intention to quit smoking regular cigarettes (US, n=1,200; UK n=1,200)			Intention to purchase e-cigarette[1] (US, n=1,200; UK n=1,200)			Perceived relative harm of e-cigarettes compared to regular cigarettes (US, n=1,092; UK n=1,075)		
	β	95% CI	p	β	95% CI	p	β	95% CI	p
**USA**									
Control (referent)									
As or more harmful	-0.126	[-0.305, 0.054]	0.169	-0.312	[-0.552, -0.073]	0.011	0.296	[0.193, 0.400]	≤0.001
Completely harmless	-0.161	[-0.340, 0.019]	0.079	0.229	[0.002, 0.456]	0.048	-0.154	[-0.258, -0.050]	0.004
Uncertainty	-0.025	[-0.204, 0.155]	0.786	-0.102	[-0.334, 0.130]	0.389	0.036	[-0.067, 0.140]	0.492
		R^2=0.869			*Pseudo R^2 = 0.205*<br>*Alpha=0: p*≤0.001			*R^2 =0.666*	
**UK**									
Control (referent)									
As or more harmful	0.063	[-0.101, 0.228]	0.451	-0.365	[-0.551, -0.178]	≤0.001	0.385	[0.297, 0.474]	≤0.001
Completely harmless	-0.079	[-0.244, 0.085]	0.344	0.034	[-0.141, 0.208]	0.707	-0.053	[-0.142, 0.035]	0.238
Uncertainty	-0.011	[-0.176, 0.154]	0.895	-0.113	[-0.289, 0.062]	0.205	-0.002	[-0.092, 0.087]	0.958
		R^2= 0.879			*Pseudo R^2 = 0.217*<br>*Alpha=0: p*≤0.001			*R^2 =0.701*	

Notes. Above models controlled for pre-exposure measure of outcome.

1. For intention to purchase e-cigarettes Negative Binomial Regression was conducted due to a zero-inflated distribution / non-normal distribution

For peer review only

only after stratification (β=0.229, 95%CI: 0.002, 0.456, p=0.048) and only among US participants.

Compared to participants assigned to the control messages, participants who viewed the as or more harmful messages were significantly more likely to perceive e-cigarettes as *more* harmful than regular cigarettes (β=0.341, 95%CI: 0.273, 0.410, p<0.001). Participants assigned to the completely harmless messages were significantly more likely to perceive e-cigarettes as *less* harmful then regular cigarettes (β=-0.106, 95%CI: -0.174, -0.037, p=0.003). These effects remained following stratification by country (UK: β=0.385, 95%CI: 0.298, 0.476, p<0.001; US: β= 0.296, 95%CI: 0.193, 0.400, p<0.001). The effect of the completely harmless misinformation on participants perceiving e-cigarettes as less harmful than cigarettes was limited to the US population after stratification (β= -0.154, 95%CI: -0.258, -0.050, p=0.004).

We additionally ran a number of sensitivity analyses owing to differences in baseline measurement between the US and the UK, and non-normality of residuals in the regression analyses. However, there were no substantial differences to report from any of the sensitivity analyses (see Table 5).

For peer review only

Table 5: Sensitivity analyses: adjusted regression analysis predicting intention to quit regular cigarettes, intention to purchase an e-cigarette and perceived relative harm of e-cigarettes compared to regular cigarettes A: includes country as a covariate, B: analysis with robust standard errors and C: analysis with bootstrapping

	Intention to quit smoking regular cigarettes (n=2,400)			Intention to purchase e-cigarette (n=2,400)			Perceived relative harm of e-cigarettes (n=2,167)		
	β	95% CI	p	β	95% CI	p	β	95% CI	p
A									
Control (referent)									
As or more harmful	-0.031	[-0.153, 0.091]	0.620	-0.337	[-0.485, -0.189]	≤0.001	0.341	[0.273, 0.410]	≤0.001
Completely harmless	-0.120	[-0.241, 0.002]	0.054	0.111	[-0.029, 0.250]	0.120	-0.105	[-0.174, -0.037]	0.003
Uncertainty	-0.017	[-0.139, 0.104]	0.779	-0.106	[-0.247, 0.035]	0.142	-0.017	[-0.052, 0.086]	0.628
B									
Control (referent)									
As or more harmful	-0.031	[-0.156, 0.095]	0.633	-0.339	[-0.499, -0.179]	≤0.001	0.341	[0.271, 0.412]	≤0.001
Completely harmless	-0.120	[-0.241, 0.002]	0.054	0.111	[-0.036, 0.258]	0.141	-0.106	[-0.163, -0.048]	≤0.001
Uncertainty	-0.017	[-0.132, 0.097]	0.767	-0.106	[-0.253, 0.042]	0.160	0.018	[-0.044, 0.079]	0.572
C									
Control (referent)									
As or more harmful	-0.031	[-0.147, 0.085]	0.605	-0.339	[-0.493, -0.185]	≤0.001	0.341	[0.280, 0.403]	≤0.001
Completely harmless	-0.120	[-0.237, 0.002]	0.047	0.111	[-0.040, 0.262]	0.151	-0.106	[-0.168, -0.044]	0.001
Uncertainty	-0.017	[-0.151, 0.116]	0.799	-0.106	[-0.239, 0.028]	0.121	0.018	[-0.051, 0.087]	0.617

For peer review only

DISCUSSION

Our results suggest that exposure to misinformation about e-cigarette harms influences adult smokers' decisions to purchase e-cigarettes and their perceived relative harm of e-cigarettes, compared to regular cigarettes. To our knowledge this is the first study to test the effect of brief exposure to misinformation and uncertainty about e-cigarette harms on Twitter on smokers' intentions to quit smoking, intentions to use e-cigarettes and perceptions of relative harm. Both US and UK samples of adult smokers were adversely affected by misinformation about e-cigarettes.

These findings are important because they show that misinformation about e-cigarettes may be hindering efforts to reduce the burden of tobacco smoking on current smokers. There is consensus that debunking or correcting exposure to misinformation is extremely challenging, common techniques have even been found to further engrain misinformation.(14,30) Reducing exposure to misinformation has its own challenges, as misinformation on social media spreads more pervasively than accurate information and the spread is due to mostly human actions, rather than automated bots.(31) In addition, it is often hard to categorise content as misinformation, especially when the evidence around a given health topic is inconclusive, or the way the information is communicated is unclear. This creates challenges in both harnessing algorithms to alert users to misinformation and also communicating ways to spot misinformation. These points, combined with our findings, have the potential to undermine the efforts of the public health community to reduce harm among current smokers. However, innovative health communication approaches need to be developed and tested to both reduce exposure to and counter misinformation using effective harm reduction and health promotion strategies. Strategies are already being employed by social media platforms to address the problem of

For peer review only

misinformation, for example, downranking content and removing or blocking users with content identified as misinformation. While it may be difficult to keep up with and identify health misinformation as such, it is possible to warn smokers of the problem of misinformation and encourage them to seek out their health information from official sources. Health care providers' should be aware that their patients may have seen misinformation on social media and hold incorrect beliefs about e-cigarettes. They should always correct these and consider the ways they can help their patients to identify accurate health information. Finally, governments and policy makers should make sure all social media searches for e-cigarettes are flagged with official health guidance. They should also regulate all forms of misinformation on social media and improve people's awareness and ability to find accurate information.

There are several limitations of the study, first, we excluded visual content from the exposures to ensure that the format of tweets was consistent across conditions and participants were focused on the content of the tweets. However, prior studies indicate that visual cues within e-cigarette advertisements are associated with perceptions about and decisions to use e-cigarettes.(7,26) Second, health misinformation is spread in different ways. We used Twitter due to our team's experience of handling their data, limitations with accessing data from other platforms (e.g. Facebook) and documented prevalence of health misinformation on Twitter.(27,28) Third, there is the issue of the reliability of self-reported smoking compared to biochemical verification of smoking status. However, given that we used an online self-administered survey, it is unlikely to have a big impact on participants' answers. Further, it's been shown that self-reported smoking prevalence, checked by biochemical verification, was underestimated by only 0.6% in the US and 2.8% in the UK.(29) Fourth, our study sample was not fully representative of the populations they were drawn from. For example, White people make up 86% of the UK

For peer review only

population, but represented 93.3% of the UK sample in this study, which may mean our findings are not generalisable. Finally, there was an outbreak of e-cigarette or vaping product use-associated lung injuries that were first identified in August 2019 in the US and subsequently traced to products containing Tetrahydrocannabinol (THC) from the illicit market. This outbreak, in combination with the different contexts of the two countries, may have influenced participants' views on e-cigarettes during the time of the study data collection. However, because of the experimental design to randomly assign participants into conditions, we do not anticipate that this would have biased our findings systematically.

Future research should focus on identifying the factors that make misinformation effective and how it is perceived by exposed individuals. Conducting research using different social media platforms, study designs and analytical tools, and focusing on analysing the message or communication factors are all important. According to our study, Facebook was overwhelmingly the social media platform used by these participants. It would therefore be interesting to replicate this research using Facebook. Second, there is a need to explore the role of cognitive factors, beliefs, past experiences and other individual level factors in the effects of misinformation. For instance, based on the theory of bias assimilation stating that people gravitate to information they have previously heard, future research should test whether the observed results could be explained by the fact that many individuals were previously exposed to misinformation. Third, it is important to refine and further develop a reliable algorithm that could distinguish between accurate and misinformation about e-cigarettes. With the amount of information that is currently generated by users on different social media platforms, an automated approach of identifying misinformation could be most cost-effective and timely. Nevertheless, any algorithms, evident from our prior work,(19,20) cannot achieve 100% accuracy, leading to misclassification errors

For peer review only

and require constant refinement and evaluation as new types of misinformation emerge. Fourth, we were not able to examine the impact of specific features of the tweets, for example the source of the message or the sender's authoritativeness. Future research is needed to determine the effects of varying these features on smokers' processing of misinformation about e-cigarettes. Finally, future research to evaluate the effect of longer or repeated exposures to misinformation would also be useful, to assess the effects on e-cigarette use intentions and subsequent vaping or smoking behaviours.

For peer review only

CONCLUSIONS

US and UK adult current smokers may be deterred from considering using e-cigarettes after brief exposure to tweets that e-cigarettes are as or more harmful than smoking. Conversely, US adult current smokers may be encouraged to use e-cigarettes and view them as less harmful than regular cigarettes, after exposure to tweets that e-cigarettes are completely harmless. These findings suggest that misinformation about e-cigarette harms may influence adult smokers' decisions to consider using e-cigarettes.

For peer review only

Acknowledgements: We would like to thank Cancer Research UK who funded this research.

Funding: Research reported in this publication was supported by a Cancer Policy Research Centre Innovation grant (C60153/A28664). Dr Wright is funded by a Cancer Research UK Population Research Postdoctoral Fellowship (C60153/A23895). Dr Dahne is supported by the National Institute on Drug Abuse, K23 DA045766. We would also like to thank the National Cancer Institute (NCI) for their support during the sandpit event where this research idea came about.

Competing interests: We declare no competing interests.

Contributors: CW, AT, OE, JD and JB all contributed to the original research idea. JB and YZ did the machine learning and JB and PW annotated the tweets. All authors contributed to the design of the survey instrument. CW and PW did the statistical analysis with input from all the authors. All authors contributed to the drafting and editing of the paper.

Data sharing statement: Following the completion of the research outlined in our research proposal and subsequent publications, instructions for accessing the data will be made available. Requests for data will be fielded by Dr Caroline Wright and shared with other researchers, provided a satisfactory data-sharing agreement has been completed by the requesting researcher. The data-sharing agreement will impose appropriate limitations on the secondary use of the data, with reference to the 'Samples and Data for Cancer Research: Template for Access Policy Development' document. We also plan to publish the data from the study once all research outlined in our research proposal has been submitted for publication.

Ethical approval: The University of Bristol's Institutional Review Board approved this study. The tweets used in this research are all in the public domain and participants could therefore have been exposed to this misinformation at any time. We further provided participants with a debrief of accurate information about e-cigarettes compared to regular cigarette harms as well as information about smoking cessation services.

Patient and Public Involvement: Patients were not involved in the design of this research.

For peer review only

REFERENCES

1. NASEM. Public Health Consequences of E-Cigarettes. Eaton DL, Kwan LY, Stratton K, editors. Washington (DC); 2018.
2. McNeill A, Brose LS, Calder R BL& RD. Evidence review of ecigarettes and heated tobacco products 2018. A report commissioned by Public Health England. London; 2018.
3. Smith CA, Shahab L, McNeill A, Jackson SE, Brown J, Brose L. Harm perceptions of e-cigarettes among smokers with and without mental health conditions in England: A cross-sectional population survey. Nicotine Tob Res Off J Soc Res Nicotine Tob. 2020 Jan;
4. Nyman AL, Huang J, Weaver SR, Eriksen MP. Perceived Comparative Harm of Cigarettes and Electronic Nicotine Delivery Systems. JAMA Netw Open [Internet]. 2019 Nov 20;2(11):e1915680–e1915680. Available from: https://doi.org/10.1001/jamanetworkopen.2019.15680
5. McNeill, A., Brose, L.S., Calder, R., Bauld, L., and Robson D. Vaping in England: an evidence update including mental health and pregnancy, March 2020: a report commissioned by Public Health England. London; 2020.
6. Persoskie A, O'Brien EK, Poonai K. Perceived relative harm of using e-cigarettes predicts future product switching among US adult cigarette and e-cigarette dual users. Addiction. 2019 Dec;114(12):2197–205.
7. Wang Y, McKee M, Torbica A, Stuckler D. Systematic Literature Review on the Spread of Health-related Misinformation on Social Media. Soc Sci Med [Internet]. 2019;240:112552. Available from: http://www.sciencedirect.com/science/article/pii/S0277953619305465
8. Brashers DE. Communication and Uncertainty Management. J Commun [Internet]. 2001 Sep 1;51(3):477–97. Available from: https://doi.org/10.1111/j.1460-2466.2001.tb02892.x
9. ONS. Internet access – households and individuals, Great Britain: 2019. London; 2019.
10. Finney Rutten LJ, Blake KD, Greenberg-Worisek AJ, Allen S V, Moser RP, Hesse BW. Online Health Information Seeking Among US Adults: Measuring Progress Toward a Healthy People 2020 Objective. Public Health Rep. 2019;134(6):617–25.
11. Chou W-YS, Oh A, Klein WMP. Addressing Health-Related Misinformation on Social Media. JAMA. 2018 Dec;320(23):2417–8.
12. Chong E, Xie B. The Use of Theory in Social Studies of Web 2.0. 2011. 1–10 p.
13. Walther JB, Jang J. Communication Processes in Participatory Websites. J Comput Commun [Internet]. 2012;18(1):2–15. Available from: https://doi.org/10.1111/j.1083-6101.2012.01592.x
14. Lewandowsky S, Ecker UKH, Seifert CM, Schwarz N, Cook J. Misinformation and Its Correction: Continued Influence and Successful Debiasing. Psychol Sci Public Interes [Internet]. 2012 Sep 17;13(3):106–31. Available from: https://doi.org/10.1177/1529100612451018
15. Chou WS, Prestin A, Lyons C, Wen K. Web 2.0 for Health Promotion: Reviewing the Current Evidence. 2013;103(1):e9–18. Available from: https://ajph.aphapublications.org/doi/abs/10.2105/AJPH.2012.301071
16. Fairchild AL, Bayer R, Lee JS. The E-Cigarette Debate: What Counts as Evidence? Am J Public Health [Internet]. 2019 May 16;109(7):1000–6. Available from: https://doi.org/10.2105/AJPH.2019.305107
17. Amin S, Dunn AG, Laranjo L. Social Influence in the Uptake and Use of Electronic

For peer review only

Cigarettes: A Systematic Review. Am J Prev Med. 2020 Jan;58(1):129–41.
18. Wright CL, Williams P, Elizarova O, Dahne J, Jiang B, Zhao Y, et al. Does viewing false messages about e-cigarette harms on Twitter change current smokers' perceptions of e-cigarettes in the US and the UK? A randomised controlled experiment [Internet]. ISRCTN. 2020. Available from: http://www.isrctn.com/ISRCTN16082420
19. Guo B, Ding Y, Yao L, Liang Y, Yu Z. The Future of Misinformation Detection: New Perspectives and Trends. 2019.
20. Thota, Aswini; Tilak, Priyanka; Ahluwalia, Simrat; and Lohia N. Fake News Detection: A Deep Learning Approach. SMU Data Sci Rev [Internet]. 2018;1(3). Available from: https://scholar.smu.edu/datasciencereview/vol1/iss3/10
21. Biener, L., & Abrams DB. The Contemplation Ladder: Validation of a measure of readiness to consider smoking cessation. Heal Psychol [Internet]. 1991;10(5):360–5. Available from: https://doi.org/10.1037/0278-6133.10.5.360
22. Juster FT. Consumer Buying Intentions and Purchase Probability: An Experiment in Survey Design. J Am Stat Assoc [Internet]. 1966 Sep 1;61(315):658–96. Available from: https://amstat.tandfonline.com/doi/abs/10.1080/01621459.1966.10480897
23. NIH. HINTS 5 Cycle 2 Survey Materials [Internet]. 2018. Available from: file://ads.bris.ac.uk/filestore/BRMS/Studies/E-Mute/Papers/Main_analysis_paper/HINTS5_Cycle2_Annotated_Instrument_English.pdf
24. Erdfelder E, Faul F, Buchner A. GPOWER: A general power analysis program. Behav Res Methods, Instruments, Comput [Internet]. 1996;28(1):1–11. Available from: https://doi.org/10.3758/BF03203630
25. StataCorp. Stata Statistical Software: Release 15. Texas: StataCorp LLC.; 2017.
26. King AC, Smith LJ, Fridberg DJ, Matthews AK, McNamara PJ, Cao D. Exposure to electronic nicotine delivery systems (ENDS) visual imagery increases smoking urge and desire. Psychol Addict Behav [Internet]. 2015/11/30. 2016 Feb;30(1):106–12. Available from: https://pubmed.ncbi.nlm.nih.gov/26618797
27. Allem J-P, Ferrara E, Uppu SP, Cruz TB, Unger JB. E-Cigarette Surveillance With Social Media Data: Social Bots, Emerging Topics, and Trends. JMIR Public Heal Surveill [Internet]. 2017;3(4):e98. Available from: http://publichealth.jmir.org/2017/4/e98/
28. Jamison AM, Broniatowski DA, Quinn SC. Malicious Actors on Twitter: A Guide for Public Health Researchers. Am J Public Health. 2019 May;109(5):688–92.
29. West R, Zatonski W, Przewozniak K, Jarvis MJ. Can We Trust National Smoking Prevalence Figures? Discrepancies Between Biochemically Assessed and Self-Reported Smoking Rates in Three Countries. Cancer Epidemiol Biomarkers & Prev [Internet]. 2007 Apr 1;16(4):820 LP – 822. Available from: http://cebp.aacrjournals.org/content/16/4/820.abstract
30. Cappella JN, Maloney E, Ophir Y, Brennan E. Interventions to Correct Misinformation About Tobacco Products. Tob Regul Sci [Internet]. 2015/07/01. 2015 Jul;1(2):186–97. Available from: https://pubmed.ncbi.nlm.nih.gov/27135046
31. Vosoughi S, Roy D, Aral S. The spread of true and false news online. Science (80-) [Internet]. 2018 Mar 9;359(6380):1146 LP – 1151. Available from: http://science.sciencemag.org/content/359/6380/1146.abstract

For peer review only - http://bmjopen.bmj.com/site/about/guidelines.xhtml

Figure 1. Condition 1: E-cigarettes are as or more harmful

Deborah L Hayes, Founder of I Can Do Stuff
@Deborah_L_Hayes

Replying to @matthewjdowd

How about let's focus on the matter at hand which is Big Tobacco duping an entire generation - again - on our watch. Let's get the word out and educate young and old, vaping and E-cigarettes kill. @RAI_News

9:56 PM · Sep 12, 2019 · Twitter for Android

Brea ♥
@breablackey

Replying to @Justinmb123 @jnelson_98 and @skylerleexo

Vaping is still pretty much just as dangerous as cigs bc everything goes DIRECTLY into your lungs. Oh I forgot to mention the flavoring chemicals in vapes can also cause cancer. Seriously, look up actual medical research please.

6:15 PM · Jul 21, 2019 · Twitter for iPhone

7 Likes

JIM FITZPATRICK
@jimfitzpatrick

Replying to @ivancorless

Smoking takes decades to cause cancer. Vaping, it seems, takes only a few years. The evidence is clear enough for US to ban flavoured vaping today, yep, today. The rest will follow as facts emerge, I imagine. It's pretty disgusting anyway -try it.

5:41 pm · 15 Sep 2019 · Twitter Web App

Ross Gerber
@GerberKawasaki

#JUUL should be banned immediately. Anyone thinking vaping cigarettes is better than smoking is being conned. Vaping chemicals into your lungs will kill you. Altria is a murderer. Flat out mass murder. $MO

5:34 PM · Sep 9, 2019 · Twitter for iPhone

22 Retweets **128** Likes

Figure 2. Condition 2: E-cigarettes are completely harmless

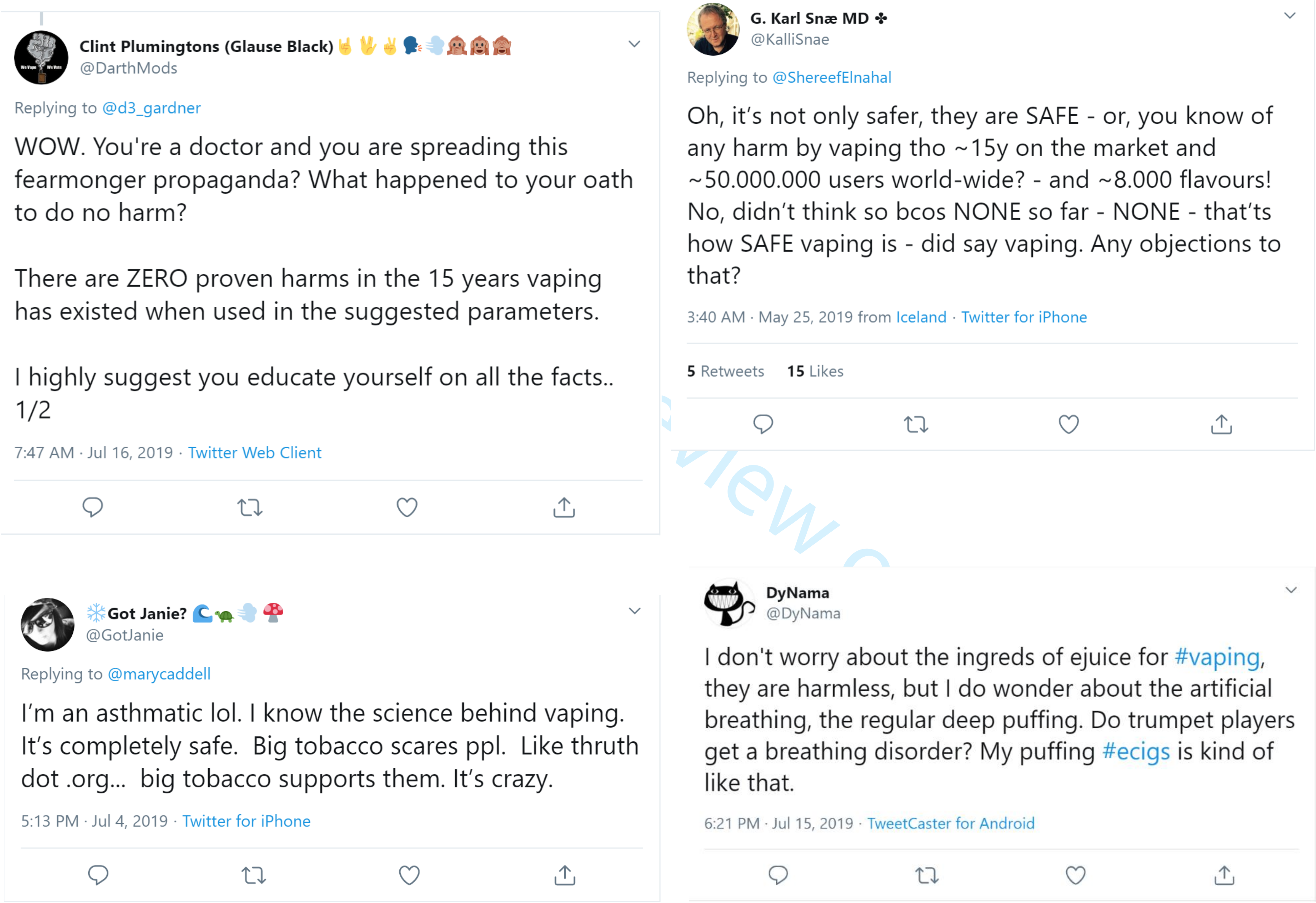

Figure 3. Condition 3: Messages expressing uncertainty about e-cigarettes

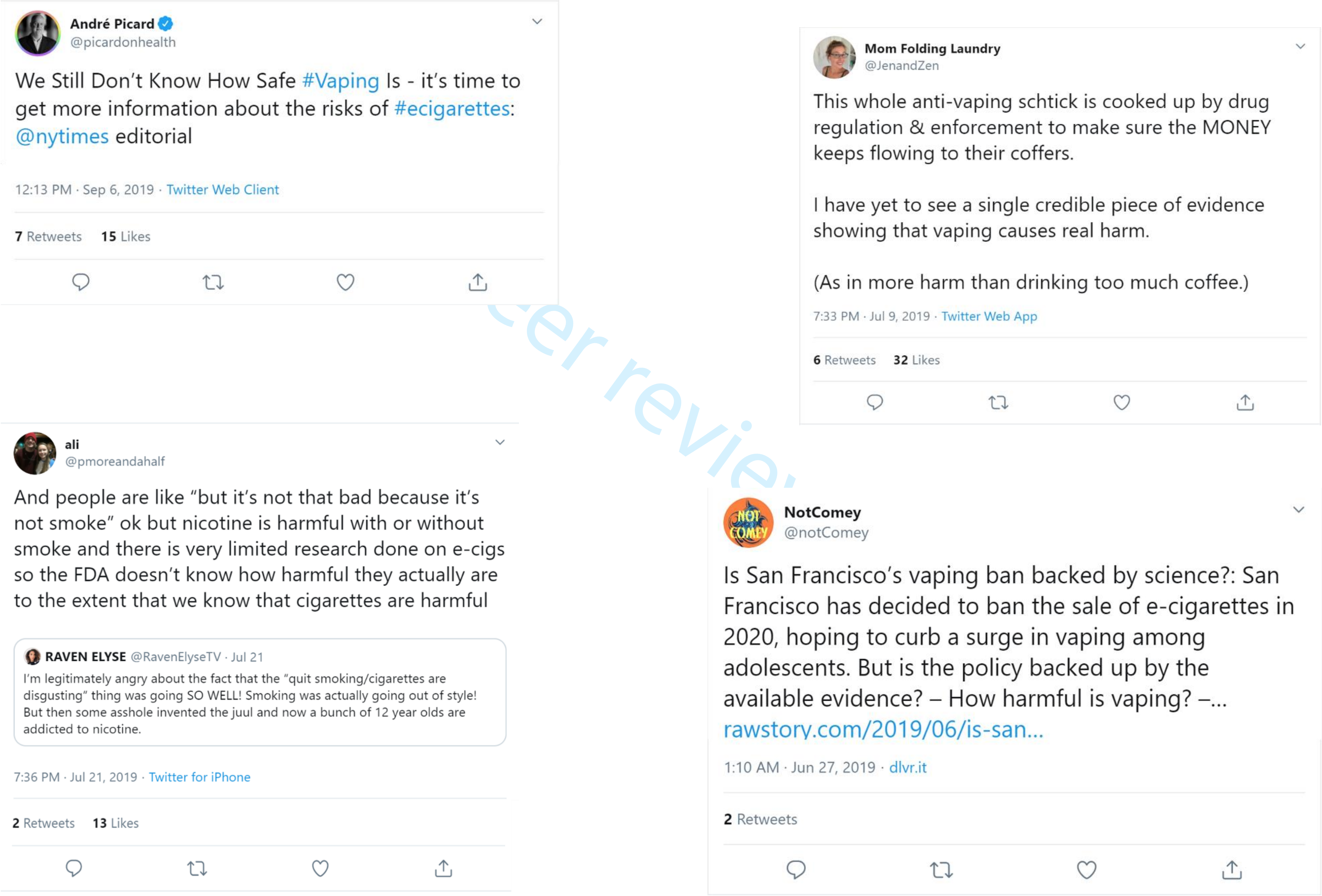

For peer review only - http://bmjopen.bmj.com/site/about/guidelines.xhtml

Figure 4. Condition 4: Messages about Physical Activity

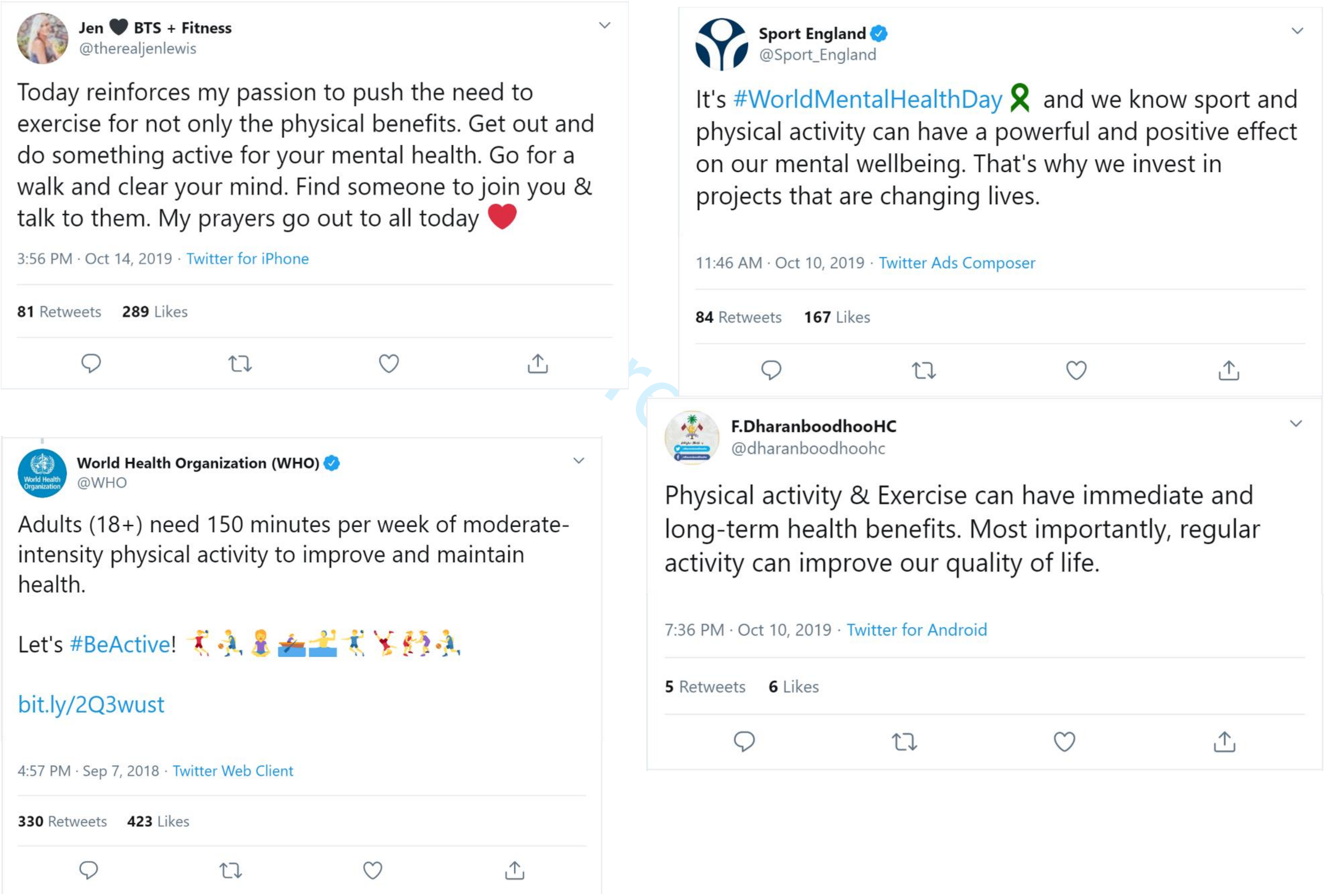

Figure 5. CONSORT Flow Diagram

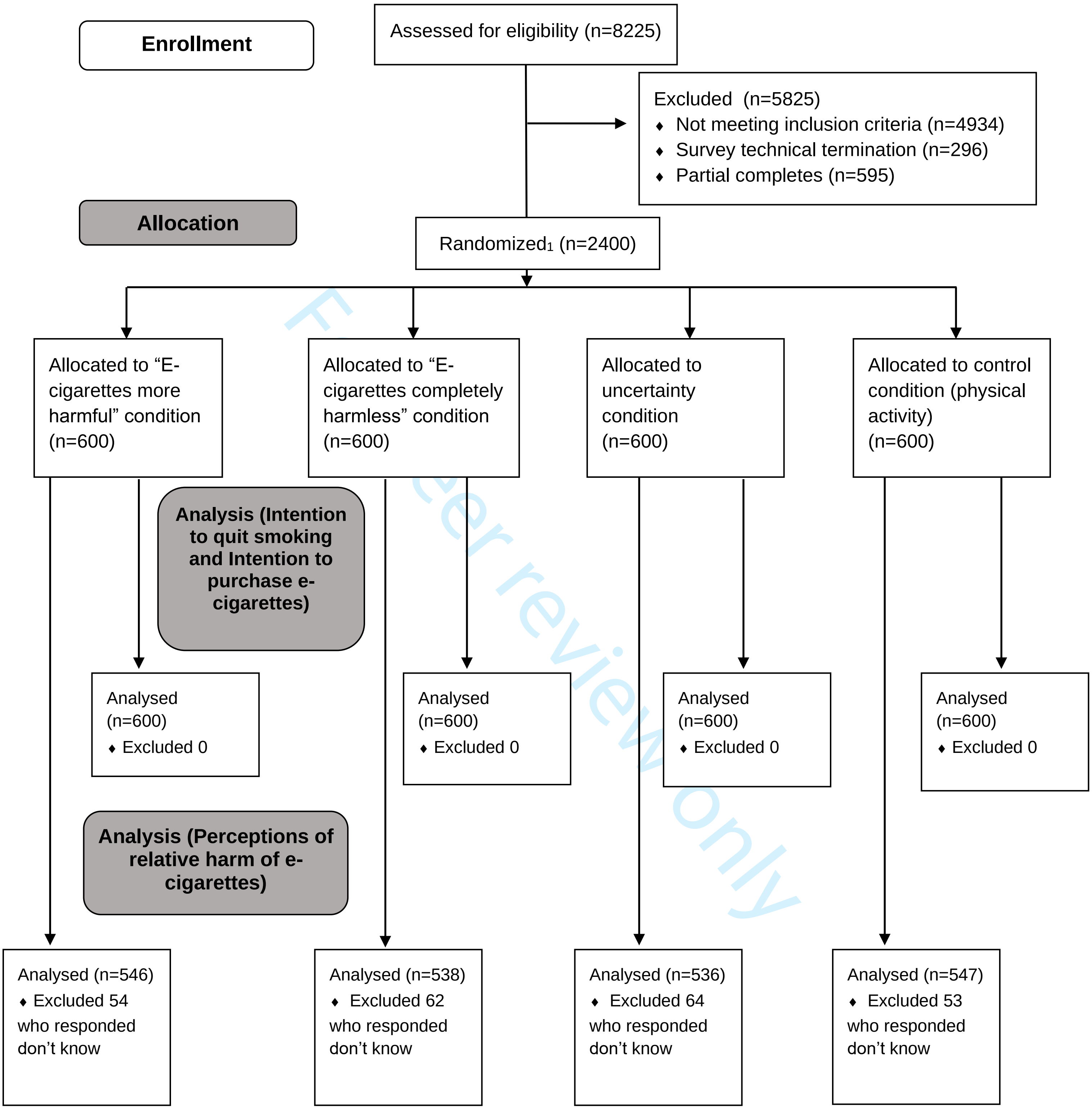

[1]. Survey recruitment used a least-fill approach; as a respondent came in, they were assigned to the exposure with the lowest complete count.

For peer review only - http://bmjopen.bmj.com/site/about/guidelines.xhtml

CONSORT 2010 checklist of information to include when reporting a randomised trial*

Section/Topic	Item No	Checklist item	Reported on page No
**Title and abstract**			
	1a	Identification as a randomised trial in the title	1
	1b	Structured summary of trial design, methods, results, and conclusions (for specific guidance see CONSORT for abstracts)	2
**Introduction**			
Background and objectives	2a	Scientific background and explanation of rationale	4
	2b	Specific objectives or hypotheses	5-6
**Methods**			
Trial design	3a	Description of trial design (such as parallel, factorial) including allocation ratio	7-8
	3b	Important changes to methods after trial commencement (such as eligibility criteria), with reasons	N/A
Participants	4a	Eligibility criteria for participants	7
	4b	Settings and locations where the data were collected	7
Interventions	5	The interventions for each group with sufficient details to allow replication, including how and when they were actually administered	8
Outcomes	6a	Completely defined pre-specified primary and secondary outcome measures, including how and when they were assessed	8-9
	6b	Any changes to trial outcomes after the trial commenced, with reasons	N/A
Sample size	7a	How sample size was determined	11
	7b	When applicable, explanation of any interim analyses and stopping guidelines	N/A
Randomisation:			
Sequence generation	8a	Method used to generate the random allocation sequence	7
	8b	Type of randomisation; details of any restriction (such as blocking and block size)	7
Allocation concealment mechanism	9	Mechanism used to implement the random allocation sequence (such as sequentially numbered containers), describing any steps taken to conceal the sequence until interventions were assigned	7
Implementation	10	Who generated the random allocation sequence, who enrolled participants, and who assigned participants to interventions	7
Blinding	11a	If done, who was blinded after assignment to interventions (for example, participants, care providers, those	N/A

For peer review only

For peer review only - http://bmjopen.bmj.com/site/about/guidelines.xhtml

		assessing outcomes) and how	
	11b	If relevant, description of the similarity of interventions	N/A
Statistical methods	12a	Statistical methods used to compare groups for primary and secondary outcomes	11
	12b	Methods for additional analyses, such as subgroup analyses and adjusted analyses	11
**Results**			
Participant flow (a diagram is strongly recommended)	13a	For each group, the numbers of participants who were randomly assigned, received intended treatment, and were analysed for the primary outcome	Figure 5
	13b	For each group, losses and exclusions after randomisation, together with reasons	Figure 5
Recruitment	14a	Dates defining the periods of recruitment and follow-up	12
	14b	Why the trial ended or was stopped	N/A
Baseline data	15	A table showing baseline demographic and clinical characteristics for each group	10
Numbers analysed	16	For each group, number of participants (denominator) included in each analysis and whether the analysis was by original assigned groups	12
Outcomes and estimation	17a	For each primary and secondary outcome, results for each group, and the estimated effect size and its precision (such as 95% confidence interval)	Table 3 p.15
	17b	For binary outcomes, presentation of both absolute and relative effect sizes is recommended	N/A
Ancillary analyses	18	Results of any other analyses performed, including subgroup analyses and adjusted analyses, distinguishing pre-specified from exploratory	Table 4 p.16
Harms	19	All important harms or unintended effects in each group (for specific guidance see CONSORT for harms)	N/A
**Discussion**			
Limitations	20	Trial limitations, addressing sources of potential bias, imprecision, and, if relevant, multiplicity of analyses	20-21
Generalisability	21	Generalisability (external validity, applicability) of the trial findings	21
Interpretation	22	Interpretation consistent with results, balancing benefits and harms, and considering other relevant evidence	19-22
**Other information**			
Registration	23	Registration number and name of trial registry	26
Protocol	24	Where the full trial protocol can be accessed, if available	26
Funding	25	Sources of funding and other support (such as supply of drugs), role of funders	24

*We strongly recommend reading this statement in conjunction with the CONSORT 2010 Explanation and Elaboration for important clarifications on all the items. If relevant, we also recommend reading CONSORT extensions for cluster randomised trials, non-inferiority and equivalence trials, non-pharmacological treatments, herbal interventions, and pragmatic trials. Additional extensions are forthcoming: for those and for up to date references relevant to this checklist, see www.consort-statement.org.

For peer review only - http://bmjopen.bmj.com/site/about/guidelines.xhtml

Invitation to take part in the study

We would like to invite you to take part in our new research study. Before you decide we would like you to understand why the research is being done and what it would involve for you. The research involves getting participants' opinions about e-cigarettes.

Please read the attached information sheet entitled **'Participant Information Sheet'** carefully. This explains what participation in this study will involve. If you are interested in and would like to participate in the study, please **also complete the attached 'CONSENT FORM'/click 'agree' on the electronic consent**.

If you have any further questions about the study, please contact Dr Caroline Wright on +44 (0)117 3314011 or email caroline.wright@bristol.ac.uk. If participants wish to make a complaint to an independent party, they can email research-governance@bristol.ac.uk.

For peer review only

Participant Information Sheet

Study title: E-MUte - Examining Prevalence, Mechanism of action and Effects of E-cigarette Misinformation on Twitter

What is the purpose of the study?

There is lots of information about e-cigarettes online, especially on social media. This type of information may be hindering efforts to reduce tobacco smoking and in turn result in more cases of cancer.

We would like to find out how information about e-cigarettes impacts on people's attitudes towards using e-cigarettes. We have created a questionnaire designed to find out what people think about e-cigarettes after seeing different types of information about e-cigarettes.

Who is participating in the study?

Smokers aged 18 and over who do not currently use e-cigarettes/vapes will participate in the study.

We are aiming to recruit adult smokers online to complete the questionnaire using ProdegeMR), an online opt-in research panel of adults recruited via tv, radio and internet sources across all regions of the US and UK. Once in the panel, members are randomly invited to take part in surveys using email invitations and messages in the member's inbox on the panel website. Study participants will receive an incentive as per ProdegeMR policies.

What do I have to do?

Participants will be asked to complete an online questionnaire about e-cigarette use. Participants will then be shown some information related to the health impacts of e-cigarettes on separate screens. Following the information messages, they will be asked to complete a second questionnaire about e-cigarette use.

At the start of the questionnaire there will be information about the study and a statement asking for participants to consent to completing the questionnaire. Each questionnaire will take approximately 20 minutes to complete. Participants will need to complete both questionnaires before receiving their incentive from ProdegeMR.

Do I have to take part?
Your participation in the study is voluntary. You can choose not to take part, or you may withdraw at any time.

Expenses and payments
Upon completion of the questionnaires, participants will receive the incentive from Prodege MR.

What are the possible disadvantages and risks of taking part?
There are no risks associated with taking part in this study.

For peer review only

What are the possible benefits of taking part?
The results from this study will help us to better understand how information impacts on people's beliefs about e-cigarettes/vapes, which in turn could help with smoking cessation programmes and reduce cancer.

What will happen if I don't want to carry on with the study?
Participants can withdraw from the study, during the survey, at any time without giving a reason. By completing the questionnaire you are agreeing to your data/information being used in the research. Once submitted this data is anonymised and cannot be retrieved.

Will my taking part in this study be kept confidential?
We will not be asking for study participants names which will make the questionnaires anonymous. All information you provide us with during the study will remain confidential. No names or identifying information will be used in any of the results, publication or presentations.

What will happen to the results of the research study?
The results from the study will be published and will be available to participants on request. You will not be identified in any report/publication.

Who is organising the research?
The project is being led by Dr Caroline Wright from the University of Bristol.

Who is funding the research?
Cancer Research UK.

What are the timescales?

The study starts in February 2019 and will end in March 2020.

Who has reviewed the study?
This project has been reviewed and approved by the University of Bristol Faculty of Health Sciences Research Ethics Committee.

Further information and contact details

If you have any further questions about the study, please contact Dr Caroline Wright on +44 (0)117 3314011 or email caroline.wright@bristol.ac.uk. If participants wish to make a complaint to an independent party, they can email research-governance@bristol.ac.uk.

For peer review only

Consent form

E-MUte - Examining Prevalence, Mechanism of action and Effects of E-cigarette Misinformation on Twitter

I confirm that I have read and understand the participant information sheet provided for the above study. I have had the opportunity to ask questions and have had these answered satisfactorily.	Yes ☐
I understand that my participation is voluntary and that I am free to withdraw from the study at any time without giving any reason.	Yes ☐
I understand that the information collected will be used to support other research in the future and may be shared openly and anonymously with other researchers.	Yes ☐
I do wish to take part in the above study.	Yes ☐

Electronic Consent:

Clicking on the "agree" button below indicates that:

- You have carefully read and understand the Participant Information Sheet
- You voluntarily agree to participate in the research study, which involves completing this questionnaire

If you agree you will be taken to the questionnaire.

o Agree

For peer review only

BMJ Open

Effects of Brief Exposure to Misinformation about E-cigarette Harms on Twitter: A Randomised Controlled Experiment

Journal:	*BMJ Open*
Manuscript ID	bmjopen-2020-045445.R1
Article Type:	Original research
Date Submitted by the Author:	10-Feb-2021
Complete List of Authors:	Wright, Caroline; University of Bristol, Bristol Medical School<br>Williams, Philippa; University of Bristol, Bristol Medical School<br>Elizarova, Olga; Play Collaborate Change<br>Dahne, Jennifer; Medical University of South Carolina College of Medicine, Psychiatry and Behavioral Sciences<br>Bian, Jiang; University of Florida<br>Zhao, Yunpeng; University of Florida, Health Outcomes & Biomedical Informatics, College of Medicine<br>Tan, Andy; University of Pennsylvania, Annenberg School for CommunicationU
**Primary Subject Heading**:	Public health
Secondary Subject Heading:	Epidemiology, Smoking and tobacco
Keywords:	PREVENTIVE MEDICINE, PUBLIC HEALTH, SOCIAL MEDICINE, EPIDEMIOLOGY

SCHOLARONE™
Manuscripts

I, the Submitting Author has the right to grant and does grant on behalf of all authors of the Work (as defined in the below author licence), an exclusive licence and/or a non-exclusive licence for contributions from authors who are: i) UK Crown employees; ii) where BMJ has agreed a CC-BY licence shall apply, and/or iii) in accordance with the terms applicable for US Federal Government officers or employees acting as part of their official duties; on a worldwide, perpetual, irrevocable, royalty-free basis to BMJ Publishing Group Ltd ("BMJ") its licensees and where the relevant Journal is co-owned by BMJ to the co-owners of the Journal, to publish the Work in this journal and any other BMJ products and to exploit all rights, as set out in our licence.

The Submitting Author accepts and understands that any supply made under these terms is made by BMJ to the Submitting Author unless you are acting as an employee on behalf of your employer or a postgraduate student of an affiliated institution which is paying any applicable article publishing charge ("APC") for Open Access articles. Where the Submitting Author wishes to make the Work available on an Open Access basis (and intends to pay the relevant APC), the terms of reuse of such Open Access shall be governed by a Creative Commons licence – details of these licences and which Creative Commons licence will apply to this Work are set out in our licence referred to above.

Other than as permitted in any relevant BMJ Author's Self Archiving Policies, I confirm this Work has not been accepted for publication elsewhere, is not being considered for publication elsewhere and does not duplicate material already published. I confirm all authors consent to publication of this Work and authorise the granting of this licence.

For peer review only

Effects of Brief Exposure to Misinformation about E-cigarette Harms on Twitter: A Randomised Controlled Experiment

Authors and affiliations:

Caroline Wright PhD[1]*, Philippa Williams MSc[1]*, Olga Elizarova MPH[2], Jennifer Dahne PhD[3], Jiang Bian PhD[4], Yunpeng Zhao MSc[4] and Andy SL Tan PhD[5]

[1] Population Health Sciences, Bristol Medical School, University of Bristol, Bristol, UK.
[2] Play Collaborate Change, Boston, USA
[3] Psychiatry and Behavioral Sciences, College of Medicine, Medical University of South Carolina
[4] Health Outcomes & Biomedical Informatics, College of Medicine, University of Florida
[5] Annenberg School for Communication, University of Pennsylvania

* Joint first authors

Corresponding author:

Caroline Wright, Population Health Sciences, Bristol Medical School, University of Bristol, BF4, Barley House, Oakfield Grove, Bristol, BS8 2BN caroline.wright@bristol.ac.uk +44(0)117 3314011

Word count (main text): 3,918
Word count (abstract): 300
References: 39
Figures: 5
Tables: 5
Supplemental figures: 1

Back to top

For peer review only

Abstract

Objectives: To assess the effect of exposure to misinformation about e-cigarette harms on Twitter on adult current smokers' intention to quit smoking cigarettes, intention to purchase e-cigarettes and perceived relative harm of e-cigarettes compared to regular cigarettes.

Setting: An online randomised controlled experiment conducted in November 2019 among United States (US) and United Kingdom (UK) current smokers.

Participants: 2,400 adult current smokers aged ≥18 years who were not current e-cigarette users recruited from an online panel. Participants' were randomised in a 1:1:1:1 ratio using a least-fill randomiser function.

Interventions: Viewing 4 tweets in random order within one of four conditions: 1) e-cigarettes are just as or more harmful than smoking, 2) e-cigarettes are completely harmless, 3) e-cigarette harms are uncertain, and 4) a control condition of tweets about physical activity.

Primary outcomes measures: Self-reported post-test intention to quit smoking cigarettes, intention to purchase e-cigarettes, and perceived relative harm of e-cigarettes compared with smoking.

Results: Among US and UK participants, after controlling for baseline measures of the outcome, exposure to tweets that e-cigarettes are as or more harmful than smoking versus control was associated with lower post-test intention to purchase e-cigarettes (β=-0.339,95%CI:-0.487,-0.191,$p<0.001$) and increased post-test perceived relative harm of e-cigarettes (β=0.341,95%CI:0.273,0.410,$p<0.001$). Among US smokers, exposure to tweets that e-cigarettes are completely harmless was associated with higher post-test intention to purchase e-cigarettes (β=0.229,95%CI:0.002,0.456,p=0.048) and lower post-test perceived relative harm of e-cigarettes (β=-0.154,95%CI:-0.258,0.050,p=0.004).

Conclusions: US and UK adult current smokers may be deterred from considering using e-cigarettes after brief exposure to tweets that e-cigarettes were just as or more harmful than smoking. Conversely, US adult current smokers may be encouraged to use e-cigarettes after exposure to tweets that e-cigarettes are completely harmless. These findings suggest that misinformation about e-cigarette harms may influence some adult smokers' decisions to consider using e-cigarettes.

Trial registration: ISRCTN registry: ISRCTN16082420.

For peer review only

Strengths and limitations of this study

- This is the first study to explore the effect of exposure to misinformation about e-cigarette harms on Twitter, showing that misinformation about e-cigarettes may be hindering efforts to reduce the burden of tobacco smoking on current smokers in the US and UK.
- We used a randomised controlled experimental design, which reduces the threat of potential confounding from observed and unobserved variables.
- We excluded visual content from our exposures and focused on Twitter: more research could be done to explore the impact of these factors.
- Our study sample did not fully represent the populations they were drawn from, which may mean our findings are not generalisable.

For peer review only

INTRODUCTION

Although e-cigarette use is not completely harmless,(1,2) there is a general agreement that the short-term health risks are considerably lower than smoking regular cigarettes.(2) Despite this growing consensus, several recent studies show misperceptions about the relative harms of e-cigarettes among current smokers are increasing in both the United States (US) and the United Kingdom (UK).(3–5) Between 2014-2019, the percentage of current adult smokers in the UK who thought e-cigarettes were less harmful than cigarettes decreased from 45% to 34% and an even lower proportions of people believe so among smokers who were not using e-cigarettes.(5) Many smokers also do not think that complete replacement of cigarettes with e-cigarettes would lead to major health benefits.(2) The US has a similar trend, with the percentage of adults perceiving e-cigarettes as less harmful than cigarettes declining from 29.3% to 25.8% between 2017-2018. Over the same period there was an increase from 1.8% to 4.4% of US adult smokers perceiving e-cigarettes as much more harmful than cigarettes.(4) The increasing trends of misperceptions about the relative harms of e-cigarettes compared with regular cigarettes are important for public health because perceived harms of e-cigarettes are associated with smokers' willingness to use e-cigarettes (6) as a harm reduction strategy.

Misperceptions, defined as false or inaccurate beliefs of the individual,(7) of e-cigarette harms may be related to exposure to misinformation — information that is incorrect or misleading.(8) Based on the state of the science of e-cigarette harms,(1,2) misinformation related to e-cigarette harms was classified as the statements that either claim that e-cigarettes are equally or more harmful than smoking regular cigarettes or are completely harmless. As the evidence-base on e-cigarette harms has developed, related media and public discussion has involved uncertainty, defined as existing "when details of the situation are ambiguous, complex, unpredictable, or

For peer review only

probabilistic; when information is unavailable or inconsistent".(9) Therefore, the impact of exposure to statements that claim the evidence of e-cigarette harms are uncertain are also important.

Health information is commonly accessed online, with 63% of UK adults using the internet to look for health-related information,(10) and 75% of US adults using the internet as their first source of health information.(11) People are increasingly encountering health information through social media platforms such as Twitter or Facebook. (12) These platforms enable users to generate and share content (13) and contrary to other media, there is often limited verification of accuracy of health information.(14,15) A systematic review found user generated content was often inconsistent with clinical guidelines and health misinformation was increasingly available online.(16) We therefore focused on misinformation of e-cigarettes occurring on social media. We used Twitter data because it is free and publicly available and because of the documented prevalence of health misinformation on Twitter.(17,18) It is estimated that just over one in 5 Americans (22%) and 45% of social media users in the UK use Twitter. (19,20)

This study comprised US and UK participants as the contrasting policy approaches toward e-cigarette use across the two countries may mean that US and UK participants view harms associated with e-cigarettes differently. While the US approach focuses on protecting non-smokers from uptake of smoking via e-cigarette use, the UK's approach emphasises e-cigarettes as a harm reduction strategy to reduce the burden of risk on current smokers.(21) Further, the UK also has much stricter regulations relating to e-cigarette advertising and nicotine content of e-liquids compared with the US.(5)

To date, most studies have focused on health misinformation in relation to communicable diseases (8) and there is limited research on misinformation related to tobacco product use

For peer review only

including e-cigarettes. While existing studies examined current perceptions of e-cigarette harms, little is known about the role of exposure to misinformation on social media on these perceptions, and consequently on e-cigarette intentions and use.(22) To address this research gap, we conducted a web-based randomised controlled experiment to assess the effect of exposure to misinformation about e-cigarette harm found on Twitter, on smokers' intentions to quit smoking, intentions to purchase e-cigarettes, and perceptions of the relative harm of e-cigarettes compared to regular cigarettes.

For peer review only

METHODS

Study design

We used a randomised controlled experimental design.(23) The study was conducted using the online consumer research panel, Prodege which recruited participants from the US and the UK via internet sources (i.e., email invitations, telephone alerts, banners and messaging on web sites and online communities). Participants' received reward points as per Prodege policies. The University of Bristol's Institutional Review Board approved this study.

Participants

Study participants were 2,400 self-reported adult smokers aged ≥18 years, who were not currently using e-cigarettes. Informed consent was obtained electronically through the survey platform.

Randomisation and masking

Following eligibility screening and having provided informed consent, participants completed baseline measures of study outcomes. Participants were then randomised to one of four experimental conditions using a least-fill randomiser function: 1) E-cigarettes are as or more harmful than regular cigarettes, 2) E-cigarettes are completely harmless, 3) Uncertain messages about e-cigarettes, and 4) Messages for the control condition about physical activity from Twitter. Participants were randomised in a 1:1:1:1 ratio using the in-built least-fill randomiser function on the Prodege survey platform. Randomisation ensures that all participants have an equal chance of being assigned to each of the exposure conditions, and as such eliminates selection bias and associated problems with confounding. Adjusting for covariates is thus not needed in subsequent analysis, provided randomisation has been successful and covariates are equally distributed across experimental conditions.

For peer review only

Procedures

Participants were told they would be shown different types of health-related information and asked for their opinions about e-cigarettes. Next, participants' provided baseline measures for: intention to quit smoking, intention to purchase e-cigarettes and perceived relative harm of e-cigarettes compared to regular cigarettes. After randomisation to a condition, they viewed one tweet at a time in random order (four tweets in total) and were asked brief questions about each tweet -perceived effectiveness of the tweet, likelihood of replying, retweeting, liking and sharing the tweet, and their emotional response to the tweet, more details of these questions can be found in supplemental material 1. Next, they completed post-test measures of the study outcomes, current tobacco use behaviours, health information exposure, media use, and sociodemographic and psychological characteristics. The average time taken to complete the survey was 29 minutes.

We captured tweets about e-cigarette harms using a validated machine learning algorithm the study team developed in an earlier phase of this research.(24,25) Using the random sample function within SPSS we selected a random 1% sample (n=499) of these tweets. Next the study team narrowed this sample of tweets to 20 tweets per experimental condition using the following criteria: 1) explicit statement that e-cigarettes were either as or more harmful than smoking, completely harmless, or uncertain; 2) no mention of children or young people: 3) no mention of specific diseases; 4) no profanities; 5) had multiple 'likes' or 'retweets'; 6) no advertising; 7) no pictures; and 8) was available publicly (i.e., not deleted).

We selected four representative tweets for each of the three experimental conditions: 1) E-cigarettes are as or more harmful, 2) E-cigarettes are completely harmless, and 3) Uncertain message about e-cigarettes. Tweets for the control condition comprised four tweets about

For peer review only

physical activity from Twitter. We selected physical activity promotion messages as the control condition to reduce potential bias due to experimenter demand and avoided topics related to e-cigarettes such as other forms of tobacco, alcohol or substance use behaviours. Figures 1-4 and supplementary material 2 displays the content from the tweets that comprised each experimental condition.

Outcome measures

Baseline and post-test intention to quit smoking

Participants were asked to consider a smoking cessation contemplation ladder.(26) They were asked: “You have told us that you are currently smoking cigarettes. Each number below represents where various smokers are in their thinking about quitting. Please enter a number that indicates where you are now, ranging from “No thought of quitting” (0) to “Taking action to quit (e.g., cutting down, enrolling in a program)” (10).

Baseline and post-test intention to purchase e-cigarettes

Participants were asked: "How probable is it that you will purchase e-cigarettes in the next month?" Answer options ranged from “No chance, almost no chance” (0) to “Certain, practically certain” (10).(27)

Baseline and post-test perceived relative harm of e-cigarettes compared to regular cigarettes

Participants were asked: “Compared to smoking cigarettes, would you say that electronic cigarettes are” Much less harmful (1) to much more harmful (5). This question included the option of don’t know.(28) Two hundred and thirty-three participants answered ‘don’t know’ to this question either at baseline or post-exposure and as such were not included in the analysis. Participants who answered ‘don’t know’ to the baseline question regarding relative harm

For peer review only

distribute evenly across the experimental conditions and therefore pose no problem with respect to confounding or selection bias.

Demographic and health information.

Participants were asked to provide sociodemographic information including age, sex, race, ethnicity, highest education level, number of days of cigarette smoking in the past 30 days, ever use of e-cigarettes, information search about e-cigarettes, and social media use (see Table 1).

For peer review only

Table 1: Sociodemographic characteristics of study sample by experimental condition and country

	US				UK			
**Condition**	1: as or more	2: completely harmless	3: uncertainty	4: control	1: as or more	2: completely harmless	3: uncertainty	4: control
**Characteristics**	n=300	n=300	n=300	n=300	n=300	n=300	n=300	n=300
**Age: Mean (SD)**	50.5 (13.6)	50.0 (13.6)	50.0 (14.7)	50.3 (13.5)	44.1 (14.6)	44.2 (14.4)	44.0 (14.8)	42.8 (14.6)
**Sex: No. (%)**								
Female	153 (51.0)	154 (51.3)	154 (51.3)	140 (46.7)	126 (42.0)	136 (45.3)	125 (41.7)	135 (45.0)
**US Race: No. (%)**								
White	206 (68.7)	214 (71.3)	211 (70.3)	220 (73.3)				
Black or African American	51 (17.0)	47 (15.7)	52 (17.3)	51 (17.0)				
Other races	43 (14.3)	39 (13.0)	37 (12.3)	29 (9.7)				
US Ethnicity: No (%)								
Non-Hispanic	271 (90.3)	269 (89.7)	270 (90.0)	274 (91.3)				
Hispanic	29 (8.7)	31 (10.3)	30 (10.0)	26 (8.7)				
**UK Ethnicity: No. (%)**								
White					284 (94.7)	276 (92.0)	278 (92.7)	282 (94.0)
Other ethnicity					16 (5.3)	24 (8.0)	22 (7.3)	18 (6.0)
**Education: No. (%)**								
High/Secondary school or below	83 (27.7)	99 (33.0)	91 (30.3)	89 (29.7)	118 (39.3)	126 (42.0)	122 (40.7)	129 (43.0)
Some college/ further education college	111 (37.0)	122 (40.7)	123 (41.0)	110 (36.7)	110 (36.7)	103 (34.3)	105 (35.0)	105 (35.0)
College/ University degree or higher	106 (35.3)	79 (26.3)	86 (28.7)	101 (33.7)	72 (24.0)	71 (23.7)	73 (24.3)	66 (22.0)
**Smoking status: Mean (SD)**								
No. days smoked in last 30 days	28.9 (4.2)	27.8 (5.9)	27.7 (5.9)	28.1 (5.4)	27.5 (6.3)	27.4 (6.9)	26.7 (7.7)	27.1 (7.00)
**E-cigarette use: No. (%)**								
Never used e-cigarettes	145 (48.3)	144 (48.0)	152 (50.7)	158 (52.7)	138 (46.0)	124 (41.3)	152 (50.7)	148 (49.3)
**Have you ever looked for e-cigarette information: No. (%)**								
Yes	75 (25.0)	81 (27.0)	58 (19.3)	76 (25.3)	72 (24.0)	78 (26.0)	74 (24.7)	82 (27.3)
**Frequency of hearing e-cigarettes harmful: No. (%)**								
Not at all	22 (7.3)	37 (12.3)	38 (12.7)	16 (5.3)	42 (14.0)	54 (18.0)	64 (21.3)	45 (15.0)
A little	68 (22.7)	78 (26.0)	83 (27.7)	67 (22.3)	123 (41.0)	113 (37.7)	131 (43.7)	122 (40.7)
Some	105 (35.0)	102 (34.0)	103 (34.3)	96 (32.0)	81 (27.0)	90 (30.0)	69 (23.0)	75 (25.0)
A lot	105 (35.0)	83 (27.7)	76 (25.3)	121 (40.3)	54 (18.0)	43 (14.3)	36 (12.0)	58 (19.3)
**Frequency of hearing e-cigarettes harmless: No. (%)**								
Not at all	132 (44.0)	107 (35.7)	150 (50.0)	137 (45.7)	122 (40.7)	97 (32.3)	115 (38.3)	145 (48.3)
A little	86 (28.7)	100 (33.3)	76 (25.3)	75 (25.0)	97 (32.3)	104 (34.7)	110 (36.7)	85 (28.3)

For peer review only

Some	56 (18.7)	61 (20.3)	55 (18.3)	61 (20.3)	53 (17.7)	68 (22.7)	53 (17.7)	58 (19.3)
A lot	26 (8.7)	32 (10.7)	19 (6.3)	27 (9.0)	28 (9.3)	31 (10.3)	22 (7.3)	12 (4.0)

Notes. Test for variance across conditions; continuous variables analysed using one-way Anova test, categorical variables analysed using χ^2 test

For peer review only

Statistical Analysis

We used GPower (version 3.1) (29) to estimate effect sizes in the outcome variables as a function of message condition, assuming two-tailed tests, with 80% power and α=0.05. Based on these analyses, a final sample size of 2,400 (600 in each arm) was deemed sufficient power to detect small effects in between-subject analyses of the main effect of condition among adult smokers (f=0.07). In stratified analyses by country, a sample size of 1,200 (300 in each arm) will also ensure sufficient power to detect small effects between conditions (f=0.10).

Analyses were completed in 2020. Randomised controlled trials aim to compare groups of participants that differ only with respect to the intervention, (30) in this case exposure to misinformation. We performed univariate analyses for all study variables. Next, we analysed whether participants across conditions differed in terms of individual characteristics. To address the study hypothesis, we utilised linear regression to predict post-test intentions to quit smoking, intentions to purchase e-cigarettes, and perceived relative harm of e-cigarettes by experimental condition compared with the control condition, adjusting for baseline measures of each outcome respectively. Owing to overdispersion of the second outcome measure, intentions to purchase e-cigarettes, we additionally ran negative binomial regression models. We also ran sensitivity analyses, including country as a covariate (owing to the differences in baseline measurements between the US and the UK; analysis using robust standard errors and bootstrapping – owing to non-normal distribution of residuals. We further conducted stratified analyses to compare the effects of experimental condition on each study outcome among US and UK participants separately. We also tested for interactions between experimental conditions and country (US or UK). Stata version 15.1 was used to conduct all analyses.(31)

For peer review only

RESULTS

Participants were 2,400 adult current smokers recruited between 8-28 November 2019 (see Figure 5: CONSORT diagram). They were aged 18-84 years (mean=47.0, SD=14.58), 46.8% were female, 70.9% of the US participants were White, 16.8% Black or African American and 12.3% were of Other racial background, 90.3% of US participants were non-Hispanic. While 93.3% of the UK cohort were White and 6.7% were from other ethnic backgrounds. Most of the cohort (82.1%) smoked cigarettes every day and 51.6% had ever used e-cigarettes. Table 1 summarises the sample characteristics by experimental condition. We found that randomisation had been achieved and all covariates were distributed evenly across the four study conditions. Three quarters of participants (n=1,804, 75.2%) had not previously searched for information on e-cigarettes. Participants were more likely to report that they had heard that e-cigarettes are more harmful than cigarettes (n=1,297, 54.0%), than hearing that e-cigarettes are harmless (n=662, 27.6%). Over half of the sample (n=1,426, 59.4%) had never used Twitter, with Facebook being the most common social media platform used several times a day (n=1,194, 49.8%).

At baseline, 25.2% of participants (n= 605) placed themselves in the middle of the intention to quit ladder (mean=5.0, SD=3.0), this was similar for both US and UK participants. Over half the participants (n=1,312, 54.7%) said that there was no chance/almost no chance that they would buy e-cigarettes in the next month. The distributions for intentions to buy, were also very similar for US and UK participants. However, there were marked differences between the two populations with respect to perceptions of the relative harm of e-cigarettes: nearly twice as many UK participants said that e-cigarettes are much less harmful than regular cigarettes compared to US participants. Similarly, more than twice as many UK participants said that e-cigarettes are less harmful than regular cigarettes (n=448, 37.3%), compared to US participants (n=222,

For peer review only

18.5%). Conversely, more than three times as many US participants thought that e-cigarettes are much more harmful than regular cigarettes (US: n=217, 18.1%, UK: n=69, 5.8%) and more than twice as many saw them as more harmful (US: n=128, 10.7%, UK: n=62, 5.2%).

We additionally compared the mean and standard deviation (SD) for the outcome measures, both pre- and post-exposure across the four conditions for the US and UK separately (Table 2). We found that pre-exposure intentions to quit and perceptions of the relative harm of e-cigarettes were generally higher and intentions to purchase e-cigarettes were generally lower among US participants.

Tables 3 and 4 summarise the results from the regression analyses. The adjusted analysis includes both the experimental condition as the exposure and the baseline measure of the outcomes. We present the adjusted analysis here. Compared with the control condition, there was no difference in the post-test intention to quit smoking among those who viewed tweets stating that e-cigarettes are as or more harmful than cigarettes, the completely harmless condition or tweets that are uncertain. The results did not change substantially in the stratified analysis (Table 4).

Compared with participants assigned to the control group, there was a statistically significant reduction in post-test intention to purchase e-cigarettes for those exposed to the as or more harmful messages (β= -0.339, 95%CI: -0.487, -0.191, $p<0.001$). In the stratified analysis, the effect of viewing as or more harmful tweets on reducing intentions to purchase e-cigarettes was observed in both US (β=-0.312, 95%CI: -0.522, -0.073, $p=0.011$) and UK samples (β= -0.365, 95%CI: -0.551, -0.178, $p<0.001$). Further, the effect of viewing tweets that e-cigarettes are completely harmless was associated with an increase in intention to purchase e-cigarettes but

For peer review only

Table 2: Outcome measures by experimental condition and country

	US				UK			
**Condition**	1	2	3	4	1	2	3	4
**Outcome measures**								
**Intentions to quit smoking**	n=300	n=300	n=300	n=300	n=300	n=300	n=300	n=300
Pre-exposure: Mean (SD)	5.16 (2.94)	5.25 (3.17)	5.23 (3.00)	5.48 (3.14)	4.72 (2.85)	4.73 (2.86)	4.83 (2.90)	4.78 (3.04)
Post-exposure: Mean (SD)	5.29 (2.96)	5.34 (3.15)	5.46 (3.04)	5.72 (3.20)	4.93 (2.90)	4.80 (2.91)	4.96 (2.89)	4.93 (3.09)
**Intentions to purchase e-cigarettes**	n=300	n=300	n=300	n=300	n=300	n=300	n=300	n=300
Pre-exposure: Mean (SD)	1.33 (2.24)	1.15 (2.08)	1.25 (2.20)	1.29 (2.23)	1.67 (2.37)	1.57 (2.33)	1.88 (2.54)	1.71 (2.47)
Post-exposure: Mean (SD)	0.98 (2.02)	1.30 (2.27)	1.16 (2.17)	1.27 (2.31)	1.21 (2.16)	1.68 (2.56)	1.73 (2.50)	1.79 (2.61)
**Perceptions of relative harms of e-cigarettes**	n=274	n=268	n=274	n=276	n=272	n=270	n=262	n=271
Pre-exposure: Mean (SD)	3.17 (1.03)	3.35 (1.28)	3.20 (1.04)	3.26 (1.10)	2.64 (0.95)	2.67 (0.93)	2.60 (0.90)	2.68 (0.90)
Post-exposure: Mean (SD)	3.45 (1.06)	3.15 (1.12)	3.22 (1.02)	3.22 (1.07)	3.02 (1.00)	2.60 (0.98)	2.60 (0.93)	2.66 (0.92)

For peer review only

Table 3: Regression analysis predicting intention to quit regular cigarettes, intention to purchase an e-cigarette and perceived relative harm of e-cigarettes compared to regular cigarettes (adjusted for baseline measures of outcome)

	Intention to quit smoking regular cigarettes (n=2,400)			Intention to purchase e-cigarette[1] (n=2,400)			Perceived relative harm of e-cigarettes compared to regular cigarettes (n=2,167)		
	β	95% CI	p	β	95% CI	p	β	95% CI	p
Control (referent)									
As or more harmful	-0.031	[-0.152, 0.091]	0.622	-0.339	[-0.487, -0.191]	≤0.001	0.341	[0.273, 0.410]	≤0.001
Completely harmless	-0.120	[-0.241, 0.002]	0.054	0.111	[-0.029, 0.250]	0.120	-0.106	[-0.174, -0.037]	0.003
Uncertainty	-0.017	[-0.139, 0.104]	0.780	-0.106	[-0.247, 0.036]	0.143	-0.018	[-0.051, 0.086]	0.615
Pre-exposure intention to quit	0.945	[0.931, 0.960]	≤0.001	-	-	-	-	-	-
Pre-exposure intention to purchase	-	-	-	0.437	[0.417, 0.458]	≤0.001	-	-	-
Pre-exposure Perceived relative harm of e-cigarettes	-	-	-	-	-	-	0.841	[0.818, 0.864]	≤0.001
		R^2= 0.874			*Pseudo R^2 = 0.2125*<br>*Alpha=0: p≤0.001*			*R^2 =0.704*	

1. For intention to purchase e-cigarettes Negative Binomial Regression was conducted due to a zero-inflated distribution / non-normal distribution

Table 4: Regression analysis predicting intention to quit regular cigarettes, intention to purchase an e-cigarette and perceived relative harm of e-cigarettes compared to regular cigarettes stratified by country of residence status (adjusted for baseline measures of outcome)

	Intention to quit smoking regular cigarettes (US, n=1,200; UK n=1,200)			Intention to purchase e-cigarette[1] (US, n=1,200; UK n=1,200)			Perceived relative harm of e-cigarettes compared to regular cigarettes (US, n=1,092; UK n=1,075)		
	β	95% CI	p	β	95% CI	p	β	95% CI	p
**USA**									
Control (referent)									
As or more harmful	-0.126	[-0.305, 0.054]	0.169	-0.312	[-0.552, -0.073]	0.011	0.296	[0.193, 0.400]	≤0.001
Completely harmless	-0.161	[-0.340, 0.019]	0.079	0.229	[0.002, 0.456]	0.048	-0.154	[-0.258, -0.050]	0.004
Uncertainty	-0.025	[-0.204, 0.155]	0.786	-0.102	[-0.334, 0.130]	0.389	0.036	[-0.067, 0.140]	0.492
Pre-exposure intention to quit	0.940	[0.920, 0.961]	≤0.001	-	-	-	-	-	-
Pre-exposure intention to purchase	-	-	-	0.475	[0.439, 0.510]	≤0.001	-	-	-
Pre-exposure Perceived relative harm of e-cigarettes	-	-	-	-	-	-	0.807	[0.773, 0.841]	≤0.001
		R^2=0.869			*Pseudo R^2 = 0.205*<br>*Alpha=0: p≤0.001*			*R^2 =0.666*	
**UK**									
Control (referent)									
As or more harmful	0.063	[-0.101, 0.228]	0.451	-0.365	[-0.551, -0.178]	≤0.001	0.385	[0.297, 0.474]	≤0.001
Completely harmless	-0.079	[-0.244, 0.085]	0.344	0.034	[-0.141, 0.208]	0.707	-0.053	[-0.142, 0.035]	0.238
Uncertainty	-0.011	[-0.176, 0.154]	0.895	-0.113	[-0.289, 0.062]	0.205	-0.002	[-0.092, 0.087]	0.958
Pre-exposure intention to quit	0.948	[0.928, 0.968]	≤0.001	-	-	-	-	-	-
Pre-exposure intention to purchase	-	-	-	0.406	[0.381, 0.431]	≤0.001	-	-	-
Pre-exposure Perceived relative harm of e-cigarettes	-	-	-	-	-	-	0.875	[0.840, 0.909]	≤0.001

For peer review only

R^2= 0.879	*Pseudo R^2 = 0.217*<br>*Alpha=0: p*≤0.001	*R^2 =0.701*

Notes. Above models controlled for pre-exposure measure of outcome.

1. For intention to purchase e-cigarettes Negative Binomial Regression was conducted due to a zero-inflated distribution / non-normal distribution

For peer review only

only after stratification (β=0.229, 95%CI: 0.002, 0.456, p=0.048) and only among US participants.

Compared to participants assigned to the control messages, participants who viewed the as or more harmful messages were significantly more likely to perceive e-cigarettes as *more* harmful than regular cigarettes (β=0.341, 95%CI: 0.273, 0.410, p<0.001). Participants assigned to the completely harmless messages were significantly more likely to perceive e-cigarettes as *less* harmful than regular cigarettes (β=-0.106, 95%CI: -0.174, -0.037, p=0.003). These effects remained following stratification by country (UK: β=0.385, 95%CI: 0.298, 0.476, p<0.001; US: β= 0.296, 95%CI: 0.193, 0.400, p<0.001). The effect of the completely harmless misinformation on participants perceiving e-cigarettes as less harmful than cigarettes was limited to the US population after stratification (β= -0.154, 95%CI: -0.258, -0.050, p=0.004).

We additionally ran a number of sensitivity analyses owing to differences in baseline measurement between the US and the UK, and non-normality of residuals in the regression analyses. However, there were no substantial differences to report from any of the sensitivity analyses (see Table 5). We additionally tested for interactions between experimental conditions and country (US or UK), but found no evidence of an effect.

For peer review only

Table 5: Sensitivity analyses: adjusted regression analysis predicting intention to quit regular cigarettes, intention to purchase an e-cigarette and perceived relative harm of e-cigarettes compared to regular cigarettes A: includes country as a covariate, B: analysis with robust standard errors and C: analysis with bootstrapping

	Intention to quit smoking regular cigarettes (n=2,400)			Intention to purchase e-cigarette (n=2,400)			Perceived relative harm of e-cigarettes (n=2,167)		
	β	95% CI	p	β	95% CI	p	β	95% CI	p
A									
Control (referent)									
As or more harmful	-0.031	[-0.153, 0.091]	0.620	-0.337	[-0.485, -0.189]	≤0.001	0.341	[0.273, 0.410]	≤0.001
Completely harmless	-0.120	[-0.241, 0.002]	0.054	0.111	[-0.029, 0.250]	0.120	-0.105	[-0.174, -0.037]	0.003
Uncertainty	-0.017	[-0.139, 0.104]	0.779	-0.106	[-0.247, 0.035]	0.142	-0.017	[-0.052, 0.086]	0.628
B									
Control (referent)									
As or more harmful	-0.031	[-0.156, 0.095]	0.633	-0.339	[-0.499, -0.179]	≤0.001	0.341	[0.271, 0.412]	≤0.001
Completely harmless	-0.120	[-0.241, 0.002]	0.054	0.111	[-0.036, 0.258]	0.141	-0.106	[-0.163, -0.048]	≤0.001
Uncertainty	-0.017	[-0.132, 0.097]	0.767	-0.106	[-0.253, 0.042]	0.160	0.018	[-0.044, 0.079]	0.572
C									
Control (referent)									
As or more harmful	-0.031	[-0.147, 0.085]	0.605	-0.339	[-0.493, -0.185]	≤0.001	0.341	[0.280, 0.403]	≤0.001
Completely harmless	-0.120	[-0.237, 0.002]	0.047	0.111	[-0.040, 0.262]	0.151	-0.106	[-0.168, -0.044]	0.001
Uncertainty	-0.017	[-0.151, 0.116]	0.799	-0.106	[-0.239, 0.028]	0.121	0.018	[-0.051, 0.087]	0.617

For peer review only

DISCUSSION

Our results suggest that exposure to misinformation about e-cigarette harms influences adult smokers' decisions to purchase e-cigarettes and their perceived relative harm of e-cigarettes, compared to regular cigarettes. To our knowledge this is the first study to test the effect of brief exposure to misinformation and uncertainty about e-cigarette harms on Twitter on smokers' intentions to quit smoking, intentions to use e-cigarettes and perceptions of relative harm. Both US and UK samples of adult smokers were adversely affected by misinformation about e-cigarettes. We also observed that US smokers who viewed tweets that e-cigs were completely harmless reported lower perceived harms of vaping and higher intentions to purchase e-cigarettes in this study. This effect was absent among UK smokers. This difference between US and UK smokers may be due to the differing policy contexts of the countries. However, further research is needed to assess underlying policy and contextual factors that explain these differences between countries in the effects of e-cigarette misinformation.

These findings are important because they show that misinformation about e-cigarettes may be hindering efforts to reduce the burden of tobacco smoking on current smokers. There is consensus that debunking or correcting exposure to misinformation is extremely challenging, common techniques have even been found to further engrain misinformation.(15,32) Reducing exposure to misinformation has its own challenges, as misinformation on social media spreads more pervasively than accurate information and the spread is due to mostly human actions, rather than automated bots.(33) In addition, it is often hard to categorise content as misinformation, especially when the evidence around a given health topic is inconclusive, or the way the information is communicated is unclear. This creates challenges in both harnessing algorithms to alert users to misinformation and also communicating ways to spot misinformation. These

For peer review only

points, combined with our findings, have the potential to undermine the efforts of the public health community to reduce harm among current smokers. However, innovative health communication approaches need to be developed and tested to both reduce exposure to and counter misinformation using effective harm reduction and health promotion strategies. Strategies are already being employed by social media platforms to address the problem of misinformation, for example, downranking content and removing or blocking users with content identified as misinformation. While it may be difficult to keep up with and identify health misinformation as such, it is possible to warn smokers of the problem of misinformation and encourage them to seek out their health information from official sources. Health care providers' should be aware that their patients may have seen misinformation on social media and hold incorrect beliefs about e-cigarettes. They should always correct these and consider the ways they can help their patients to identify accurate health information. Finally, governments and policy makers should make sure all social media searches for e-cigarettes are flagged with official health guidance. They should also regulate all forms of misinformation on social media and improve people's awareness and ability to find accurate information.

There are several limitations of the study, first, we excluded visual content from the exposures to ensure that the format of tweets was consistent across conditions and participants were focused on the content of the tweets. However, prior studies indicate that visual cues within e-cigarette advertisements are associated with perceptions about and decisions to use e-cigarettes.(8,34) Second, health misinformation is spread in different ways. We used Twitter data because it is free and publicly available and because of the documented prevalence of health misinformation on Twitter.(17,18) However, over half of participants (59%) indicated they did not use Twitter meaning they may not be familiar with viewing or engaging with tweets. To address this, we

For peer review only

included definitions of each of these engagement behaviours, prior to responding to questions on the likelihood of replying, retweeting, liking, or sharing each message. Further, our findings are still useful because intentions are strong predictors of behaviour, as shown by Ajzen's Theory of Planned Behavior.(35) Misinformation is ubiquitous - Allcott and colleagues found a total of 672 sites producing false stories or unique fake news sites.(36) Stories from these sites are shared on Facebook, Twitter and cross-posted on other social media platforms. Therefore, while this sample may not be exposed to misinformation on Twitter in real life, they are likely exposed via different channels. Third, there is the issue of the reliability of self-reported smoking compared to biochemical verification of smoking status. However, given that we used an online self-administered survey, it is unlikely to have a big impact on participants' answers. Further, it's been shown that self-reported smoking prevalence, checked by biochemical verification, was underestimated by only 0.6% in the US and 2.8% in the UK.(37) Fourth, our study sample was not fully representative of the populations they were drawn from. For example, White people make up 86% of the UK population, but represented 93.3% of the UK sample in this study, which may mean our findings are not generalisable. Fifth, previous research on health misinformation on social media identified important factors that might play a role in the mechanism of action of misinformation. Among those factors are the type of content, the source of the message, the sender's authoritativeness, the argument length, the novelty, timing, repetition and hashtags. We were not able to examine the impact of these message features in detail. Future research is needed to determine the effects of varying these features on smokers' processing of misinformation about e-cigarettes. (33,38,39) Finally, there was an outbreak of e-cigarette or vaping product use-associated lung injuries that were first identified in August 2019 in the US and subsequently traced to products containing Tetrahydrocannabinol (THC) from the

For peer review only

illicit market. This outbreak, in combination with the different contexts of the two countries, may have influenced participants' views on e-cigarettes during the time of the study data collection. However, because of the experimental design to randomly assign participants into conditions, we do not anticipate that this would have biased our findings systematically.

Future research should focus on identifying the factors that make misinformation effective and how it is perceived by exposed individuals. Conducting research using different social media platforms, study designs and analytical tools, and focusing on analysing the message or communication factors are all important. According to our study, Facebook was overwhelmingly the social media platform used by these participants. It would therefore be interesting to replicate this research using Facebook. Second, there is a need to explore the role of cognitive factors, beliefs, past experiences and other individual level factors in the effects of misinformation. For instance, based on the theory of bias assimilation stating that people gravitate to information they have previously heard, future research should test whether the observed results could be explained by the fact that many individuals were previously exposed to misinformation. Third, it is important to refine and further develop a reliable algorithm that could distinguish between accurate and misinformation about e-cigarettes. With the amount of information that is currently generated by users on different social media platforms, an automated approach of identifying misinformation could be most cost-effective and timely. Nevertheless, any algorithms, evident from our prior work,(24,25) cannot achieve 100% accuracy, leading to misclassification errors and require constant refinement and evaluation as new types of misinformation emerge. Fourth, we were not able to examine the impact of specific features of the tweets, for example the source of the message or the sender's authoritativeness. Future research is needed to determine the effects of varying these features on smokers' processing of misinformation about e-cigarettes.

For peer review only

Future research to evaluate the effect of longer or repeated exposures to misinformation would also be useful, to assess the effects on e-cigarette use intentions and subsequent vaping or smoking behaviours. Finally, future research could extend our analysis to include behaviours as well as intentions.

For peer review only

CONCLUSIONS

US and UK adult current smokers may be deterred from considering using e-cigarettes after brief exposure to tweets that e-cigarettes are as or more harmful than smoking. Conversely, US adult current smokers may be encouraged to use e-cigarettes and view them as less harmful than regular cigarettes, after exposure to tweets that e-cigarettes are completely harmless. These findings suggest that misinformation about e-cigarette harms may influence adult smokers' decisions to consider using e-cigarettes.

For peer review only

Acknowledgements: We would like to thank Cancer Research UK who funded this research.

Funding: Research reported in this publication was supported by a Cancer Policy Research Centre Innovation grant (C60153/A28664). Dr Wright is funded by a Cancer Research UK Population Research Postdoctoral Fellowship (C60153/A23895). Dr Dahne is supported by the National Institute on Drug Abuse, K23 DA045766. We would also like to thank the National Cancer Institute (NCI) for their support during the sandpit event where this research idea came about.

Competing interests: We declare no competing interests.

Contributors: CW, AT, OE, JD and JB all contributed to the original research idea. JB and YZ did the machine learning and JB and PW annotated the tweets. All authors contributed to the design of the survey instrument. CW and PW did the statistical analysis with input from all the authors. All authors contributed to the drafting and editing of the paper.

Data sharing statement: Following the completion of the research outlined in our research proposal and subsequent publications, instructions for accessing the data will be made available. Requests for data will be fielded by Dr Caroline Wright and shared with other researchers, provided a satisfactory data-sharing agreement has been completed by the requesting researcher. The data-sharing agreement will impose appropriate limitations on the secondary use of the data, with reference to the 'Samples and Data for Cancer Research: Template for Access Policy Development' document. We also plan to publish the data from the study once all research outlined in our research proposal has been submitted for publication.

Data availability: Unpublished data, the details of which can be found here: https://doi.org/10.1186/ISRCTN16082420 are available upon reasonable request.

Ethical approval: The University of Bristol's Institutional Review Board approved this study. The tweets used in this research are all in the public domain and participants could therefore have been exposed to this misinformation at any time. We further provided participants with a debrief of accurate information about e-cigarettes compared to regular cigarette harms as well as information about smoking cessation services.

Patient and Public Involvement: Patients were not involved in the design of this research.

Figure captions:

For peer review only

Figure 1. Condition 1 E-cigarettes are as or more harmful

Figure 2. Condition 2 E-cigarettes are completely harmless

Figure 3. Condition 3 Messages expressing uncertainty about e-cigarettes

Figure 4. Condition 4 Messages about Physical Activity

Figure 5. CONSORT Flow Diagram

For peer review only

REFERENCES

1. NASEM. Public Health Consequences of E-Cigarettes. Eaton DL, Kwan LY, Stratton K, editors. Washington (DC); 2018.
2. McNeill A, Brose LS, Calder R BL& RD. Evidence review of ecigarettes and heated tobacco products 2018. A report commissioned by Public Health England. London; 2018.
3. Smith CA, Shahab L, McNeill A, Jackson SE, Brown J, Brose L. Harm perceptions of e-cigarettes among smokers with and without mental health conditions in England: A cross-sectional population survey. Nicotine Tob Res Off J Soc Res Nicotine Tob. 2020 Jan;
4. Nyman AL, Huang J, Weaver SR, Eriksen MP. Perceived Comparative Harm of Cigarettes and Electronic Nicotine Delivery Systems. JAMA Netw Open [Internet]. 2019 Nov 20;2(11):e1915680–e1915680. Available from: https://doi.org/10.1001/jamanetworkopen.2019.15680
5. McNeill, A., Brose, L.S., Calder, R., Bauld, L., and Robson D. Vaping in England: an evidence update including mental health and pregnancy, March 2020: a report commissioned by Public Health England. London; 2020.
6. Persoskie A, O'Brien EK, Poonai K. Perceived relative harm of using e-cigarettes predicts future product switching among US adult cigarette and e-cigarette dual users. Addiction. 2019 Dec;114(12):2197–205.
7. Southwell BG, Niederdeppe J, Cappella JN, Gaysynsky A, Kelley DE, Oh A, et al. Misinformation as a Misunderstood Challenge to Public Health. Am J Prev Med. 2019 Aug;57(2):282–5.
8. Wang Y, McKee M, Torbica A, Stuckler D. Systematic Literature Review on the Spread of Health-related Misinformation on Social Media. Soc Sci Med [Internet]. 2019;240:112552. Available from: http://www.sciencedirect.com/science/article/pii/S0277953619305465
9. Brashers DE. Communication and Uncertainty Management. J Commun [Internet]. 2001 Sep 1;51(3):477–97. Available from: https://doi.org/10.1111/j.1460-2466.2001.tb02892.x
10. ONS. Internet access – households and individuals, Great Britain: 2019. London; 2019.
11. Finney Rutten LJ, Blake KD, Greenberg-Worisek AJ, Allen S V, Moser RP, Hesse BW. Online Health Information Seeking Among US Adults: Measuring Progress Toward a Healthy People 2020 Objective. Public Health Rep. 2019;134(6):617–25.
12. Chou W-YS, Oh A, Klein WMP. Addressing Health-Related Misinformation on Social Media. JAMA. 2018 Dec;320(23):2417–8.
13. Chong E, Xie B. The Use of Theory in Social Studies of Web 2.0. 2011. 1–10 p.
14. Walther JB, Jang J. Communication Processes in Participatory Websites. J Comput Commun [Internet]. 2012;18(1):2–15. Available from: https://doi.org/10.1111/j.1083-6101.2012.01592.x
15. Lewandowsky S, Ecker UKH, Seifert CM, Schwarz N, Cook J. Misinformation and Its Correction: Continued Influence and Successful Debiasing. Psychol Sci Public Interes [Internet]. 2012 Sep 17;13(3):106–31. Available from: https://doi.org/10.1177/1529100612451018
16. Chou WS, Prestin A, Lyons C, Wen K. Web 2.0 for Health Promotion: Reviewing the Current Evidence. 2013;103(1):e9–18. Available from: https://ajph.aphapublications.org/doi/abs/10.2105/AJPH.2012.301071
17. Allem J-P, Ferrara E, Uppu SP, Cruz TB, Unger JB. E-Cigarette Surveillance With Social

Media Data: Social Bots, Emerging Topics, and Trends. JMIR Public Heal Surveill [Internet]. 2017;3(4):e98. Available from: http://publichealth.jmir.org/2017/4/e98/
18. Jamison AM, Broniatowski DA, Quinn SC. Malicious Actors on Twitter: A Guide for Public Health Researchers. Am J Public Health. 2019 May;109(5):688–92.
19. Pew Research Center. Social Media Use in 2019. 2019.
20. We Are Social. Digital 2020: The United Kingdom [Internet]. London; 2020. Available from: https://wearesocial.com/uk/digital-2020-uk
21. Fairchild AL, Bayer R, Lee JS. The E-Cigarette Debate: What Counts as Evidence? Am J Public Health [Internet]. 2019 May 16;109(7):1000–6. Available from: https://doi.org/10.2105/AJPH.2019.305107
22. Amin S, Dunn AG, Laranjo L. Social Influence in the Uptake and Use of Electronic Cigarettes: A Systematic Review. Am J Prev Med. 2020 Jan;58(1):129–41.
23. Wright CL, Williams P, Elizarova O, Dahne J, Jiang B, Zhao Y, et al. Does viewing false messages about e-cigarette harms on Twitter change current smokers' perceptions of e-cigarettes in the US and the UK? A randomised controlled experiment [Internet]. ISRCTN. 2020. Available from: http://www.isrctn.com/ISRCTN16082420
24. Guo B, Ding Y, Yao L, Liang Y, Yu Z. The Future of Misinformation Detection: New Perspectives and Trends. 2019.
25. Thota, Aswini; Tilak, Priyanka; Ahluwalia, Simrat; and Lohia N. Fake News Detection: A Deep Learning Approach. SMU Data Sci Rev [Internet]. 2018;1(3). Available from: https://scholar.smu.edu/datasciencereview/vol1/iss3/10
26. Biener, L., & Abrams DB. The Contemplation Ladder: Validation of a measure of readiness to consider smoking cessation. Heal Psychol [Internet]. 1991;10(5):360–5. Available from: https://doi.org/10.1037/0278-6133.10.5.360
27. Juster FT. Consumer Buying Intentions and Purchase Probability: An Experiment in Survey Design. J Am Stat Assoc [Internet]. 1966 Sep 1;61(315):658–96. Available from: https://amstat.tandfonline.com/doi/abs/10.1080/01621459.1966.10480897
28. NIH. HINTS 5 Cycle 2 Survey Materials [Internet]. 2018. Available from: file://ads.bris.ac.uk/filestore/BRMS/Studies/E-Mute/Papers/Main_analysis_paper/HINTS5_Cycle2_Annotated_Instrument_English.pdf
29. Erdfelder E, Faul F, Buchner A. GPOWER: A general power analysis program. Behav Res Methods, Instruments, Comput [Internet]. 1996;28(1):1–11. Available from: https://doi.org/10.3758/BF03203630
30. Moher D, Hopewell S, Schulz KF, Montori V, Gøtzsche PC, Devereaux PJ, et al. CONSORT 2010 Explanation and Elaboration: updated guidelines for reporting parallel group randomised trials. J Clin Epidemiol [Internet]. 2010;63(8):e1–37. Available from: http://www.sciencedirect.com/science/article/pii/S0895435610001034
31. StataCorp. Stata Statistical Software: Release 15. Texas: StataCorp LLC.; 2017.
32. Cappella JN, Maloney E, Ophir Y, Brennan E. Interventions to Correct Misinformation About Tobacco Products. Tob Regul Sci [Internet]. 2015/07/01. 2015 Jul;1(2):186–97. Available from: https://pubmed.ncbi.nlm.nih.gov/27135046
33. Vosoughi S, Roy D, Aral S. The spread of true and false news online. Science (80-) [Internet]. 2018 Mar 9;359(6380):1146 LP – 1151. Available from: http://science.sciencemag.org/content/359/6380/1146.abstract
34. King AC, Smith LJ, Fridberg DJ, Matthews AK, McNamara PJ, Cao D. Exposure to electronic nicotine delivery systems (ENDS) visual imagery increases smoking urge and

For peer review only

desire. Psychol Addict Behav [Internet]. 2015/11/30. 2016 Feb;30(1):106–12. Available from: https://pubmed.ncbi.nlm.nih.gov/26618797

35. Ajzen I. The theory of planned behavior. Organ Behav Hum Decis Process [Internet]. 1991;50(2):179–211. Available from: https://www.sciencedirect.com/science/article/pii/074959789190020T

36. Allcott H, Gentzkow M, Yu C. Trends in the diffusion of misinformation on social media. Res Polit [Internet]. 2019 Apr 1;6(2):2053168019848554. Available from: https://doi.org/10.1177/2053168019848554

37. West R, Zatonski W, Przewozniak K, Jarvis MJ. Can We Trust National Smoking Prevalence Figures? Discrepancies Between Biochemically Assessed and Self-Reported Smoking Rates in Three Countries. Cancer Epidemiol Biomarkers & Prev [Internet]. 2007 Apr 1;16(4):820 LP – 822. Available from: http://cebp.aacrjournals.org/content/16/4/820.abstract

38. Zhou J, Liu F, Zhou H. Understanding health food messages on Twitter for health literacy promotion. Perspect Public Health [Internet]. 2018 Mar 7;138(3):173–9. Available from: https://doi.org/10.1177/1757913918760359

39. Wood MJ. Propagating and Debunking Conspiracy Theories on Twitter During the 2015-2016 Zika Virus Outbreak. Cyberpsychol Behav Soc Netw [Internet]. 2018/07/18. 2018 Aug;21(8):485–90. Available from: https://pubmed.ncbi.nlm.nih.gov/30020821

For peer review only

Figure 1. Condition 1: E-cigarettes are as or more harmful

How about let's focus on the matter at hand which is Big Tobacco duping an entire generation - again - on our watch. Let's get the word out and educate young and old, vaping and E-cigarettes kill. @RAI_News

Smoking takes decades to cause cancer. Vaping, it seems, takes only a few years. The evidence is clear enough for US to ban flavoured vaping today, yep, today. The rest will follow as facts emerge, I imagine. It's pretty disgusting anyway -try it.

Vaping is still pretty much just as dangerous as cigs bc everything goes DIRECTLY into your lungs. Oh I forgot to mention the flavoring chemicals in vapes can also cause cancer. Seriously, look up actual medical research please.

#JUUL should be banned immediately. Anyone thinking vaping cigarettes is better than smoking is being conned. Vaping chemicals into your lungs will kill you. Altria is a murderer. Flat out mass murder. $MO

Figure 2. Condition 2: E-cigarettes are completely harmless

WOW. You're a doctor and you are spreading this fearmonger propaganda? What happened to your oath to do no harm?

There are ZERO proven harms in the 15 years vaping has existed when used in the suggested parameters.

I highly suggest you educate yourself on all the facts.. 1/2

I'm an asthmatic lol. I know the science behind vaping. It's completely safe. Big tobacco scares ppl. Like thruth dot .org... big tobacco supports them. It's crazy.

Oh, it's not only safer, they are SAFE - or, you know of any harm by vaping tho ~15y on the market and ~50.000.000 users world-wide? - and ~8.000 flavours! No, didn't think so bcos NONE so far - NONE - that'ts how SAFE vaping is - did say vaping. Any objections to that?

I don't worry about the ingreds of ejuice for #vaping, they are harmless, but I do wonder about the artificial breathing, the regular deep puffing. Do trumpet players get a breathing disorder? My puffing #ecigs is kind of like that.

Figure 3. Condition 3: Messages expressing uncertainty about e-cigarettes

We Still Don't Know How Safe #Vaping Is - it's time to get more information about the risks of #ecigarettes: @nytimes editorial

And people are like "but it's not that bad because it's not smoke" ok but nicotine is harmful with or without smoke and there is very limited research done on e-cigs so the FDA doesn't know how harmful they actually are to the extent that we know that cigarettes are harmful

This whole anti-vaping schtick is cooked up by drug regulation & enforcement to make sure the MONEY keeps flowing to their coffers.

I have yet to see a single credible piece of evidence showing that vaping causes real harm.

(As in more harm than drinking too much coffee.)

Is San Francisco's vaping ban backed by science?: San Francisco has decided to ban the sale of e-cigarettes in 2020, hoping to curb a surge in vaping among adolescents. But is the policy backed up by the available evidence? – How harmful is vaping? –...

For peer review only - http://bmjopen.bmj.com/site/about/guidelines.xhtml

Figure 4. Condition 4: Messages about Physical Activity

Today reinforces my passion to push the need to exercise for not only the physical benefits. Get out and do something active for your mental health. Go for a walk and clear your mind. Find someone to join you & talk to them. My prayers go out to all today ❤️

Adults (18+) need 150 minutes per week of moderate-intensity physical activity to improve and maintain health.

Let's #BeActive!

It's #WorldMentalHealthDay and we know sport and physical activity can have a powerful and positive effect on our mental wellbeing. That's why we invest in projects that are changing lives.

Physical activity & Exercise can have immediate and long-term health benefits. Most importantly, regular activity can improve our quality of life.

Figure 5. CONSORT Flow Diagram

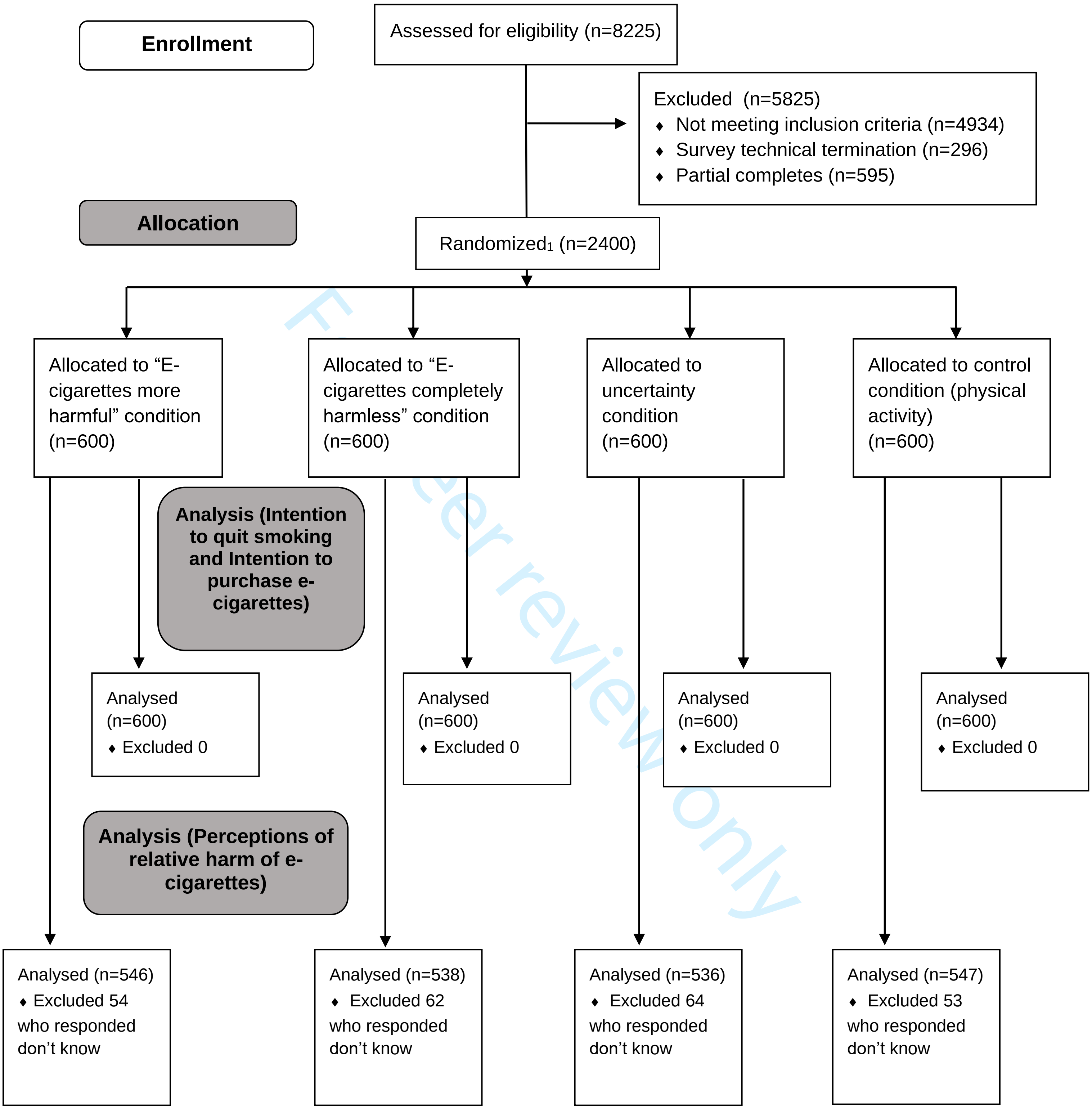

1. Survey recruitment used a least-fill approach; as a respondent came in, they were assigned to the exposure with the lowest complete count.

For peer review only

Supplemental material 1: additional questions about tweets

Questions asked after each tweet

We are interested in learning whether you would reply, retweet, like, and share this message, whether you use Twitter regularly or not. A reply is a response to another person's Tweet, a Retweet is a re-posting of a Tweet, Likes are used to show appreciation for a Tweet, and you can Share a tweet via direct message, text message, or email.

Please rate how much you disagree or agree with the following statements about the message you just saw.

a. PE1: This message is worth remembering.
b. PE2: This message grabbed my attention.
c. PE3: This message is powerful.
d. PE4: This message is informative.
e. PE5: This message is meaningful to me.
f. PE6: This message is convincing.

1. Strongly disagree (1)
2. Disagree (2)
3. Neither disagree nor agree (3)
4. Agree (4)
5. Strongly agree (5)

Intentions of replyiong/retweeting/liking/sharing Tweets

Are you likely to Reply to this message?

1. Yes
2. No

Are you likely Retweet this message?

1. Yes
2. No

Are you likely to Like this message?

1. Yes
2. No

Are you likely to Share this message?

1. Yes
2. No

Emotional responses

Please mark an answer for each question in the table below. When thinking about e-cigarettes, does the message you just saw make you feel…

a. Scared
b. Hopeful
c. Worried

For peer review only

d. Happy
e. Angry
f. Relieved

1. Not at all
2. A little
3. Some
4. A lot
5. Completely

For peer review only

Supplemental material 2: experimental conditions

Experimental condition	Exposure 1	Exposure 2	Exposure 3	Exposure 4
1: E-cigarettes are as or more harmful than regular cigarettes	Let's focus on the matter at hand which is big tobacco duping an entire generation – again – on our watch. Let’s get the word out and educate young people and old, vaping and e-cigarettes kill.	Smoking takes decades to cause cancer. Vaping, it seems, takes only a few years. The evidence is clear enough for US to ban flavoured vaping today, yep, today. The rest will follow as facts emerge, I imagine. It’s pretty disgusting anyway – try it.	Vaping is still pretty much as dangerous as cigs because everything goes directly into your lungs. Oh I forgot to mention the flavouring chemicals in vapes can also cause cancer. Seriously, look up actual medical research please.	Juul should be banned immediately. Anyone thinking vaping cigarettes is better than smoking is being conned. Vaping chemicals into your lungs will kill you. Altria is a murderer. Flat out mass murder.
2: E-cigarettes are completely harmless	Wow. You’re a doctor and you are spreading this fearmonger propaganda? What happened to your oath do no harm? There are zero proven harms in the 15 years vaping has existed when used in the suggested parameters. I highly suggest you educate yourself on all the facts.	I’m an asthmatic lol. I know the science behind vaping. It’s completely safe. Big tobacco scares ppl. Like truth dot .org…big tobacco supports them. It’s crazy.	Oh, it’s not only safer, they are safe – or, you know of any harm by vaping though ~15y on the market and ~50.000.000 users world-wide? – and ~8.000 flavours! No, didn’t think so because none. That’s how safe vaping is – did say vaping. Any objections to that?	I don’t worry about the ingredients of e-juice for vaping, they are harmless, but I do wonder about the artificial breathing, the regular deep puffing. Do trumpet players get a breathing disorder? My puffing e-cigs is kind of like that.
3: Messages expressing uncertainty about e-cigarettes	We still don’t know how safe vaping is – it’s time to get more information about the risks of e-cigarettes.	And people are like “but it’s not that bad because it’s not smoke” ok but nicotine is harmful with or without smoke and there is very limited research done on e-cigs so the FDA doesn’t know how harmful they	This whole anti-vaping schtick is cooked up by drug regulation & enforcement to make sure the money keeps flowing to their coffers. I have yet to see a single credible piece of evidence that vaping causes real harm.	Is San Francisco’s vaping ban backed by science? San Francisco has decided to ban the sale of e-cigarettes in 2020, hoping to curb a surge in vaping among adolescents. But is the policy backed up by the available

		actually are to the extent that we know cigarettes are harmful.	(As in more harm than drinking too much coffee.)	evidence? How harmful is vaping?
4: Messages about physical activity (control condition)	Today reinforces my passion to push the need to exercise for not only the physical benefits. Get out and do something active for your mental health. Go for a walk and clear your mind. Find someone to join you and talk to them. My prayers go out to all today.	Adults (those aged 18 or older) need 150 minutes per week of moderate intensity physical activity to improve and maintain health.	It's world mental health day and we know sport and physical activity can have powerful and positive effect on our wellbeing. That's why we invest in projects that are changing lives.	Physical activity and exercise can have immediate and long-term health benefits. Most importantly, regular activity can improve our quality of life.

For peer review only

CONSORT 2010 checklist of information to include when reporting a randomised trial*

Section/Topic	Item No	Checklist item	Reported on page No
**Title and abstract**			
	1a	Identification as a randomised trial in the title	1
	1b	Structured summary of trial design, methods, results, and conclusions (for specific guidance see CONSORT for abstracts)	2
**Introduction**			
Background and objectives	2a	Scientific background and explanation of rationale	4
	2b	Specific objectives or hypotheses	5-6
**Methods**			
Trial design	3a	Description of trial design (such as parallel, factorial) including allocation ratio	7-8
	3b	Important changes to methods after trial commencement (such as eligibility criteria), with reasons	N/A
Participants	4a	Eligibility criteria for participants	7
	4b	Settings and locations where the data were collected	7
Interventions	5	The interventions for each group with sufficient details to allow replication, including how and when they were actually administered	8
Outcomes	6a	Completely defined pre-specified primary and secondary outcome measures, including how and when they were assessed	8-9
	6b	Any changes to trial outcomes after the trial commenced, with reasons	N/A
Sample size	7a	How sample size was determined	11
	7b	When applicable, explanation of any interim analyses and stopping guidelines	N/A
Randomisation:			
Sequence generation	8a	Method used to generate the random allocation sequence	7
	8b	Type of randomisation; details of any restriction (such as blocking and block size)	7
Allocation concealment mechanism	9	Mechanism used to implement the random allocation sequence (such as sequentially numbered containers), describing any steps taken to conceal the sequence until interventions were assigned	7
Implementation	10	Who generated the random allocation sequence, who enrolled participants, and who assigned participants to interventions	7
Blinding	11a	If done, who was blinded after assignment to interventions (for example, participants, care providers, those	N/A

For peer review only - http://bmjopen.bmj.com/site/about/guidelines.xhtml
For peer review only

		assessing outcomes) and how	
	11b	If relevant, description of the similarity of interventions	N/A
Statistical methods	12a	Statistical methods used to compare groups for primary and secondary outcomes	11
	12b	Methods for additional analyses, such as subgroup analyses and adjusted analyses	11
**Results**			
Participant flow (a diagram is strongly recommended)	13a	For each group, the numbers of participants who were randomly assigned, received intended treatment, and were analysed for the primary outcome	Figure 5
	13b	For each group, losses and exclusions after randomisation, together with reasons	Figure 5
Recruitment	14a	Dates defining the periods of recruitment and follow-up	12
	14b	Why the trial ended or was stopped	N/A
Baseline data	15	A table showing baseline demographic and clinical characteristics for each group	10
Numbers analysed	16	For each group, number of participants (denominator) included in each analysis and whether the analysis was by original assigned groups	12
Outcomes and estimation	17a	For each primary and secondary outcome, results for each group, and the estimated effect size and its precision (such as 95% confidence interval)	Table 3 p.15
	17b	For binary outcomes, presentation of both absolute and relative effect sizes is recommended	N/A
Ancillary analyses	18	Results of any other analyses performed, including subgroup analyses and adjusted analyses, distinguishing pre-specified from exploratory	Table 4 p.16
Harms	19	All important harms or unintended effects in each group (for specific guidance see CONSORT for harms)	N/A
**Discussion**			
Limitations	20	Trial limitations, addressing sources of potential bias, imprecision, and, if relevant, multiplicity of analyses	20-21
Generalisability	21	Generalisability (external validity, applicability) of the trial findings	21
Interpretation	22	Interpretation consistent with results, balancing benefits and harms, and considering other relevant evidence	19-22
**Other information**			
Registration	23	Registration number and name of trial registry	26
Protocol	24	Where the full trial protocol can be accessed, if available	26
Funding	25	Sources of funding and other support (such as supply of drugs), role of funders	24

*We strongly recommend reading this statement in conjunction with the CONSORT 2010 Explanation and Elaboration for important clarifications on all the items. If relevant, we also recommend reading CONSORT extensions for cluster randomised trials, non-inferiority and equivalence trials, non-pharmacological treatments, herbal interventions, and pragmatic trials. Additional extensions are forthcoming: for those and for up to date references relevant to this checklist, see www.consort-statement.org.

For peer review only - http://bmjopen.bmj.com/site/about/guidelines.xhtml

Invitation to take part in the study

We would like to invite you to take part in our new research study. Before you decide we would like you to understand why the research is being done and what it would involve for you. The research involves getting participants' opinions about e-cigarettes.

Please read the attached information sheet entitled **'Participant Information Sheet'** carefully. This explains what participation in this study will involve. If you are interested in and would like to participate in the study, please **also complete the attached 'CONSENT FORM'/click 'agree' on the electronic consent**.

If you have any further questions about the study, please contact Dr Caroline Wright on +44 (0)117 3314011 or email caroline.wright@bristol.ac.uk. If participants wish to make a complaint to an independent party, they can email research-governance@bristol.ac.uk.

For peer review only

Participant Information Sheet

Study title: E-MUte - Examining Prevalence, Mechanism of action and Effects of E-cigarette Misinformation on Twitter

What is the purpose of the study?

There is lots of information about e-cigarettes online, especially on social media. This type of information may be hindering efforts to reduce tobacco smoking and in turn result in more cases of cancer.

We would like to find out how information about e-cigarettes impacts on people's attitudes towards using e-cigarettes. We have created a questionnaire designed to find out what people think about e-cigarettes after seeing different types of information about e-cigarettes.

Who is participating in the study?

Smokers aged 18 and over who do not currently use e-cigarettes/vapes will participate in the study.

We are aiming to recruit adult smokers online to complete the questionnaire using ProdegeMR), an online opt-in research panel of adults recruited via tv, radio and internet sources across all regions of the US and UK. Once in the panel, members are randomly invited to take part in surveys using email invitations and messages in the member's inbox on the panel website. Study participants will receive an incentive as per ProdegeMR policies.

What do I have to do?

Participants will be asked to complete an online questionnaire about e-cigarette use. Participants will then be shown some information related to the health impacts of e-cigarettes on separate screens. Following the information messages, they will be asked to complete a second questionnaire about e-cigarette use.

At the start of the questionnaire there will be information about the study and a statement asking for participants to consent to completing the questionnaire. Each questionnaire will take approximately 20 minutes to complete. Participants will need to complete both questionnaires before receiving their incentive from ProdegeMR.

Do I have to take part?
Your participation in the study is voluntary. You can choose not to take part, or you may withdraw at any time.

Expenses and payments
Upon completion of the questionnaires, participants will receive the incentive from Prodege MR.

What are the possible disadvantages and risks of taking part?
There are no risks associated with taking part in this study.

For peer review only

What are the possible benefits of taking part?
The results from this study will help us to better understand how information impacts on people's beliefs about e-cigarettes/vapes, which in turn could help with smoking cessation programmes and reduce cancer.

What will happen if I don't want to carry on with the study?
Participants can withdraw from the study, during the survey, at any time without giving a reason. By completing the questionnaire you are agreeing to your data/information being used in the research. Once submitted this data is anonymised and cannot be retrieved.

Will my taking part in this study be kept confidential?
We will not be asking for study participants names which will make the questionnaires anonymous. All information you provide us with during the study will remain confidential. No names or identifying information will be used in any of the results, publication or presentations.

What will happen to the results of the research study?
The results from the study will be published and will be available to participants on request. You will not be identified in any report/publication.

Who is organising the research?
The project is being led by Dr Caroline Wright from the University of Bristol.

Who is funding the research?
Cancer Research UK.

What are the timescales?

The study starts in February 2019 and will end in March 2020.

Who has reviewed the study?
This project has been reviewed and approved by the University of Bristol Faculty of Health Sciences Research Ethics Committee.

Further information and contact details

If you have any further questions about the study, please contact Dr Caroline Wright on +44 (0)117 3314011 or email caroline.wright@bristol.ac.uk. If participants wish to make a complaint to an independent party, they can email research-governance@bristol.ac.uk.

For peer review only

Consent form

E-MUte - Examining Prevalence, Mechanism of action and Effects of E-cigarette Misinformation on Twitter

I confirm that I have read and understand the participant information sheet provided for the above study. I have had the opportunity to ask questions and have had these answered satisfactorily.	Yes	☐
I understand that my participation is voluntary and that I am free to withdraw from the study at any time without giving any reason.	Yes	☐
I understand that the information collected will be used to support other research in the future and may be shared openly and anonymously with other researchers.	Yes	☐
I do wish to take part in the above study.	Yes	☐

Electronic Consent:

Clicking on the “agree” button below indicates that:

- You have carefully read and understand the Participant Information Sheet
- You voluntarily agree to participate in the research study, which involves completing this questionnaire

If you agree you will be taken to the questionnaire.

o Agree

For peer review only

BMJ Open

Effects of Brief Exposure to Misinformation about E-cigarette Harms on Twitter: A Randomised Controlled Experiment

Journal:	*BMJ Open*
Manuscript ID	bmjopen-2020-045445.R2
Article Type:	Original research
Date Submitted by the Author:	10-Jun-2021
Complete List of Authors:	Wright, Caroline; University of Bristol, Bristol Medical School<br>Williams, Philippa; University of Bristol, Bristol Medical School<br>Elizarova, Olga; Play Collaborate Change<br>Dahne, Jennifer; Medical University of South Carolina College of Medicine, Psychiatry and Behavioral Sciences<br>Bian, Jiang; University of Florida<br>Zhao, Yunpeng; University of Florida, Health Outcomes & Biomedical Informatics, College of Medicine<br>Tan, Andy; University of Pennsylvania, Annenberg School for CommunicationU
**Primary Subject Heading**:	Public health
Secondary Subject Heading:	Epidemiology, Smoking and tobacco
Keywords:	PREVENTIVE MEDICINE, PUBLIC HEALTH, SOCIAL MEDICINE, EPIDEMIOLOGY

SCHOLARONE™
Manuscripts

I, the Submitting Author has the right to grant and does grant on behalf of all authors of the Work (as defined in the below author licence), an exclusive licence and/or a non-exclusive licence for contributions from authors who are: i) UK Crown employees; ii) where BMJ has agreed a CC-BY licence shall apply, and/or iii) in accordance with the terms applicable for US Federal Government officers or employees acting as part of their official duties; on a worldwide, perpetual, irrevocable, royalty-free basis to BMJ Publishing Group Ltd ("BMJ") its licensees and where the relevant Journal is co-owned by BMJ to the co-owners of the Journal, to publish the Work in this journal and any other BMJ products and to exploit all rights, as set out in our licence.

The Submitting Author accepts and understands that any supply made under these terms is made by BMJ to the Submitting Author unless you are acting as an employee on behalf of your employer or a postgraduate student of an affiliated institution which is paying any applicable article publishing charge ("APC") for Open Access articles. Where the Submitting Author wishes to make the Work available on an Open Access basis (and intends to pay the relevant APC), the terms of reuse of such Open Access shall be governed by a Creative Commons licence – details of these licences and which Creative Commons licence will apply to this Work are set out in our licence referred to above.

Other than as permitted in any relevant BMJ Author's Self Archiving Policies, I confirm this Work has not been accepted for publication elsewhere, is not being considered for publication elsewhere and does not duplicate material already published. I confirm all authors consent to publication of this Work and authorise the granting of this licence.

For peer review only

Effects of Brief Exposure to Misinformation about E-cigarette Harms on Twitter: A Randomised Controlled Experiment

Authors and affiliations:

Caroline Wright PhD[1]*, Philippa Williams MSc[1]*, Olga Elizarova MPH[2], Jennifer Dahne PhD[3], Jiang Bian PhD[4], Yunpeng Zhao MSc[4] and Andy SL Tan PhD[5]

[1] Population Health Sciences, Bristol Medical School, University of Bristol, Bristol, UK.
[2] Play Collaborate Change, Boston, USA
[3] Psychiatry and Behavioral Sciences, College of Medicine, Medical University of South Carolina
[4] Health Outcomes & Biomedical Informatics, College of Medicine, University of Florida
[5] Annenberg School for Communication, University of Pennsylvania

* Joint first authors

Corresponding author:

Caroline Wright, Population Health Sciences, Bristol Medical School, University of Bristol, BF4, Barley House, Oakfield Grove, Bristol, BS8 2BN caroline.wright@bristol.ac.uk +44(0)117 3314011

Word count (main text): 3,918
Word count (abstract): 300
References: 39
Figures: 5
Tables: 5
Supplementary figures: 2

Back to top

For peer review only

Abstract

Objectives: To assess the effect of exposure to misinformation about e-cigarette harms on Twitter on adult current smokers' intention to quit smoking cigarettes, intention to purchase e-cigarettes and perceived relative harm of e-cigarettes compared to regular cigarettes.

Setting: An online randomised controlled experiment conducted in November 2019 among United States (US) and United Kingdom (UK) current smokers.

Participants: 2,400 adult current smokers aged ≥18 years who were not current e-cigarette users recruited from an online panel. Participants' were randomised in a 1:1:1:1 ratio using a least-fill randomiser function.

Interventions: Viewing 4 tweets in random order within one of four conditions: 1) e-cigarettes are just as or more harmful than smoking, 2) e-cigarettes are completely harmless, 3) e-cigarette harms are uncertain, and 4) a control condition of tweets about physical activity.

Primary outcomes measures: Self-reported post-test intention to quit smoking cigarettes, intention to purchase e-cigarettes, and perceived relative harm of e-cigarettes compared with smoking.

Results: Among US and UK participants, after controlling for baseline measures of the outcome, exposure to tweets that e-cigarettes are as or more harmful than smoking versus control was associated with lower post-test intention to purchase e-cigarettes (β=-0.339,95%CI:-0.487,-0.191,p<0.001) and increased post-test perceived relative harm of e-cigarettes (β=0.341,95%CI:0.273,0.410,p<0.001). Among US smokers, exposure to tweets that e-cigarettes are completely harmless was associated with higher post-test intention to purchase e-cigarettes (β=0.229,95%CI:0.002,0.456,p=0.048) and lower post-test perceived relative harm of e-cigarettes (β=-0.154,95%CI:-0.258,-0.050,p=0.004).

Conclusions: US and UK adult current smokers may be deterred from considering using e-cigarettes after brief exposure to tweets that e-cigarettes were just as or more harmful than smoking. Conversely, US adult current smokers may be encouraged to use e-cigarettes after exposure to tweets that e-cigarettes are completely harmless. These findings suggest that misinformation about e-cigarette harms may influence some adult smokers' decisions to consider using e-cigarettes.

Trial registration: ISRCTN registry: ISRCTN16082420.

For peer review only

Strengths and limitations of this study

- This is the first study to explore the effect of exposure to misinformation about e-cigarette harms on Twitter, showing that after brief exposure to tweets that e-cigarettes are as or more harmful than smoking, current smokers may be deterred from using e-cigarettes (measured with intention to purchase e-cigarettes) as a harm reduction strategy. They are also more likely to wrongly believe that e-cigarettes are more harmful than regular cigarettes. We used a randomised controlled experimental design, which reduces the threat of potential confounding from observed and unobserved variables.
- We excluded visual content from our exposures and focused on Twitter: more research could be done to explore the impact of these factors.
- Our study sample did not fully represent the populations they were drawn from, which may mean our findings are not generalisable.

For peer review only

INTRODUCTION

Although e-cigarette use is not completely harmless,(1,2) there is a general agreement that the short-term health risks are considerably lower than smoking regular cigarettes.(2) Despite this growing consensus, several recent studies show misperceptions about the relative harms of e-cigarettes among current smokers are increasing in both the United States (US) and the United Kingdom (UK).(3–5) Between 2014-2019, the percentage of current adult smokers in the UK who thought e-cigarettes were less harmful than cigarettes decreased from 45% to 34% and an even lower proportions of people believe so among smokers who were not using e-cigarettes.(5) Many smokers also do not think that complete replacement of cigarettes with e-cigarettes would lead to major health benefits.(2) The US has a similar trend, with the percentage of adults perceiving e-cigarettes as less harmful than cigarettes declining from 29.3% to 25.8% between 2017-2018. Over the same period there was an increase from 1.8% to 4.4% of US adult smokers perceiving e-cigarettes as much more harmful than cigarettes.(4) The increasing trends of misperceptions about the relative harms of e-cigarettes compared with regular cigarettes are important for public health because perceived harms of e-cigarettes are associated with smokers' willingness to use e-cigarettes (6) as a harm reduction strategy.

Misperceptions, defined as false or inaccurate beliefs of the individual,(7) of e-cigarette harms may be related to exposure to misinformation — information that is incorrect or misleading.(8) Based on the state of the science of e-cigarette harms,(1,2) misinformation related to e-cigarette harms was classified as the statements that either claim that e-cigarettes are equally or more harmful than smoking regular cigarettes or are completely harmless. As the evidence-base on e-cigarette harms has developed, related media and public discussion has involved uncertainty, defined as existing "when details of the situation are ambiguous, complex, unpredictable, or

For peer review only

probabilistic; when information is unavailable or inconsistent".(9) Therefore, the impact of exposure to statements that claim the evidence of e-cigarette harms are uncertain are also important.

Health information is commonly accessed online, with 63% of UK adults using the internet to look for health-related information,(10) and 75% of US adults using the internet as their first source of health information.(11) People are increasingly encountering health information through social media platforms such as Twitter or Facebook. (12) These platforms enable users to generate and share content (13) and contrary to other media, there is often limited verification of accuracy of health information.(14,15) A systematic review found user generated content was often inconsistent with clinical guidelines and health misinformation was increasingly available online.(16) We therefore focused on misinformation of e-cigarettes occurring on social media. We used Twitter data because it is free and publicly available and because of the documented prevalence of health misinformation on Twitter.(17,18) It is estimated that just over one in 5 Americans (22%) and 45% of social media users in the UK use Twitter. (19,20)

This study comprised US and UK participants as the contrasting policy approaches toward e-cigarette use across the two countries may mean that US and UK participants view harms associated with e-cigarettes differently. While the US approach focuses on protecting non-smokers from uptake of smoking via e-cigarette use, the UK's approach emphasises e-cigarettes as a harm reduction strategy to reduce the burden of risk on current smokers.(21) Further, the UK also has much stricter regulations relating to e-cigarette advertising and nicotine content of e-liquids compared with the US.(5)

To date, most studies have focused on health misinformation in relation to communicable diseases (8) and there is limited research on misinformation related to tobacco product use

For peer review only

including e-cigarettes. While existing studies examined current perceptions of e-cigarette harms, little is known about the role of exposure to misinformation on social media on these perceptions, and consequently on e-cigarette intentions and use.(22) To address this research gap, we conducted a web-based randomised controlled experiment to assess the effect of exposure to misinformation about e-cigarette harm found on Twitter, on smokers' intentions to quit smoking, intentions to purchase e-cigarettes, and perceptions of the relative harm of e-cigarettes compared to regular cigarettes.

For peer review only

METHODS

Study design

We used a randomised controlled experimental design.(23) The study was conducted using the online consumer research panel, Prodege which recruited participants from the US and the UK via internet sources (i.e., email invitations, telephone alerts, banners and messaging on web sites and online communities). Participants' received reward points as per Prodege policies. The University of Bristol's Institutional Review Board approved this study.

Participants

Study participants were 2,400 self-reported adult smokers aged ≥18 years, who were not currently using e-cigarettes. Informed consent was obtained electronically through the survey platform.

Randomisation and masking

Following eligibility screening and having provided informed consent, participants completed baseline measures of study outcomes. Participants were then randomised to one of four experimental conditions using a least-fill randomiser function: 1) E-cigarettes are as or more harmful than regular cigarettes, 2) E-cigarettes are completely harmless, 3) Uncertain messages about e-cigarettes, and 4) Messages for the control condition about physical activity from Twitter. Participants were randomised in a 1:1:1:1 ratio using the in-built least-fill randomiser function on the Prodege survey platform. Randomisation ensures that all participants have an equal chance of being assigned to each of the exposure conditions, and as such eliminates selection bias and associated problems with confounding. Adjusting for covariates is thus not needed in subsequent analysis, provided randomisation has been successful and covariates are equally distributed across experimental conditions.

For peer review only

Procedures

Participants were told they would be shown different types of health-related information and asked for their opinions about e-cigarettes. Next, participants' provided baseline measures for: intention to quit smoking, intention to purchase e-cigarettes and perceived relative harm of e-cigarettes compared to regular cigarettes. After randomisation to a condition, they viewed one tweet at a time in random order (four tweets in total) and were asked brief questions about each tweet -perceived effectiveness of the tweet, likelihood of replying, retweeting, liking and sharing the tweet, and their emotional response to the tweet, more details of these questions can be found in supplementary material 1. Next, they completed post-test measures of the study outcomes, current tobacco use behaviours, health information exposure, media use, and sociodemographic and psychological characteristics. The average time taken to complete the survey was 29 minutes.

We captured tweets about e-cigarette harms using a validated machine learning algorithm the study team developed in an earlier phase of this research.(24,25) Using the random sample function within SPSS we selected a random 1% sample (n=499) of these tweets. Next the study team narrowed this sample of tweets to 20 tweets per experimental condition using the following criteria: 1) explicit statement that e-cigarettes were either as or more harmful than smoking, completely harmless, or uncertain; 2) no mention of children or young people: 3) no mention of specific diseases; 4) no profanities; 5) had multiple 'likes' or 'retweets'; 6) no advertising; 7) no pictures; and 8) was available publicly (i.e., not deleted).

We selected four representative tweets for each of the three experimental conditions: 1) E-cigarettes are as or more harmful, 2) E-cigarettes are completely harmless, and 3) Uncertain message about e-cigarettes. Tweets for the control condition comprised four tweets about

For peer review only

physical activity from Twitter. We selected physical activity promotion messages as the control condition to reduce potential bias due to experimenter demand and avoided topics related to e-cigarettes such as other forms of tobacco, alcohol or substance use behaviours. Figures 1-4 and supplementary material 2 displays the content from the tweets that comprised each experimental condition.

Outcome measures

Baseline and post-test intention to quit smoking

Participants were asked to consider a smoking cessation contemplation ladder.(26) They were asked: “You have told us that you are currently smoking cigarettes. Each number below represents where various smokers are in their thinking about quitting. Please enter a number that indicates where you are now, ranging from “No thought of quitting” (0) to “Taking action to quit (e.g., cutting down, enrolling in a program)” (10).

Baseline and post-test intention to purchase e-cigarettes

Participants were asked: "How probable is it that you will purchase e-cigarettes in the next month?" Answer options ranged from “No chance, almost no chance” (0) to “Certain, practically certain” (10).(27)

Baseline and post-test perceived relative harm of e-cigarettes compared to regular cigarettes

Participants were asked: “Compared to smoking cigarettes, would you say that electronic cigarettes are” Much less harmful (1) to much more harmful (5). This question included the option of don’t know.(28) Two hundred and thirty-three participants answered ‘don’t know’ to this question either at baseline or post-exposure and as such were not included in the analysis. Participants who answered ‘don’t know’ to the baseline question and post-exposure regarding

For peer review only

relative harm distribute evenly across the experimental conditions and therefore pose no problem with respect to confounding or selection bias.

Demographic and health information.

Participants were asked to provide sociodemographic information including age, sex, race, ethnicity, highest education level, number of days of cigarette smoking in the past 30 days, ever use of e-cigarettes, information search about e-cigarettes, and social media use (see Table 1).

For peer review only

Table 1: Sociodemographic characteristics of study sample by experimental condition and country

	US				UK			
**Condition**	1: as or more	2: completely harmless	3: uncertainty	4: control	1: as or more	2: completely harmless	3: uncertainty	4: control
**Characteristics**	n=300	n=300	n=300	n=300	n=300	n=300	n=300	n=300
**Age: Mean (SD)**	50.5 (13.6)	50.0 (13.6)	50.0 (14.7)	50.3 (13.5)	44.1 (14.6)	44.2 (14.4)	44.0 (14.8)	42.8 (14.6)
**Sex: No. (%)**								
Female	153 (51.0)	154 (51.3)	154 (51.3)	140 (46.7)	126 (42.0)	136 (45.3)	125 (41.7)	135 (45.0)
**US Race: No. (%)**								
White	206 (68.7)	214 (71.3)	211 (70.3)	220 (73.3)				
Black or African American	51 (17.0)	47 (15.7)	52 (17.3)	51 (17.0)				
Other races	43 (14.3)	39 (13.0)	37 (12.3)	29 (9.7)				
US Ethnicity: No (%)								
Non-Hispanic	271 (90.3)	269 (89.7)	270 (90.0)	274 (91.3)				
Hispanic	29 (8.7)	31 (10.3)	30 (10.0)	26 (8.7)				
**UK Ethnicity: No. (%)**								
White					284 (94.7)	276 (92.0)	278 (92.7)	282 (94.0)
Other ethnicity					16 (5.3)	24 (8.0)	22 (7.3)	18 (6.0)
**Education: No. (%)**								
High/Secondary school or below	83 (27.7)	99 (33.0)	91 (30.3)	89 (29.7)	118 (39.3)	126 (42.0)	122 (40.7)	129 (43.0)
Some college/ further education college	111 (37.0)	122 (40.7)	123 (41.0)	110 (36.7)	110 (36.7)	103 (34.3)	105 (35.0)	105 (35.0)
College/ University degree or higher	106 (35.3)	79 (26.3)	86 (28.7)	101 (33.7)	72 (24.0)	71 (23.7)	73 (24.3)	66 (22.0)
**Smoking status: Mean (SD)**								
No. days smoked in last 30 days	28.9 (4.2)	27.8 (5.9)	27.7 (5.9)	28.1 (5.4)	27.5 (6.3)	27.4 (6.9)	26.7 (7.7)	27.1 (7.00)
**E-cigarette use: No. (%)**								
Never used e-cigarettes	145 (48.3)	144 (48.0)	152 (50.7)	158 (52.7)	138 (46.0)	124 (41.3)	152 (50.7)	148 (49.3)
**Have you ever looked for e-cigarette information: No. (%)**								
Yes	75 (25.0)	81 (27.0)	58 (19.3)	76 (25.3)	72 (24.0)	78 (26.0)	74 (24.7)	82 (27.3)
**Frequency of hearing e-cigarettes harmful: No. (%)**								
Not at all	22 (7.3)	37 (12.3)	38 (12.7)	16 (5.3)	42 (14.0)	54 (18.0)	64 (21.3)	45 (15.0)
A little	68 (22.7)	78 (26.0)	83 (27.7)	67 (22.3)	123 (41.0)	113 (37.7)	131 (43.7)	122 (40.7)
Some	105 (35.0)	102 (34.0)	103 (34.3)	96 (32.0)	81 (27.0)	90 (30.0)	69 (23.0)	75 (25.0)
A lot	105 (35.0)	83 (27.7)	76 (25.3)	121 (40.3)	54 (18.0)	43 (14.3)	36 (12.0)	58 (19.3)
**Frequency of hearing e-cigarettes harmless: No. (%)**								
Not at all	132 (44.0)	107 (35.7)	150 (50.0)	137 (45.7)	122 (40.7)	97 (32.3)	115 (38.3)	145 (48.3)
A little	86 (28.7)	100 (33.3)	76 (25.3)	75 (25.0)	97 (32.3)	104 (34.7)	110 (36.7)	85 (28.3)

For peer review only

Some	56 (18.7)	61 (20.3)	55 (18.3)	61 (20.3)	53 (17.7)	68 (22.7)	53 (17.7)	58 (19.3)
A lot	26 (8.7)	32 (10.7)	19 (6.3)	27 (9.0)	28 (9.3)	31 (10.3)	22 (7.3)	12 (4.0)
**Twitter use:**								
Several times a day	21 (7.0)	29 (9.7)	21 (7.0)	29 (9.7)	31 (10.3)	31 (10.3)	39 (13.0)	32 (10.7)
About once a day	16 (5.3)	15 (5.0)	32 (10.7)	23 (7.7)	22 (7.3)	32 (10.7)	33 (11.0)	24 (8.0)
A few times a week	28 (9.3)	31 (10.3)	25 (8.3)	22 (7.3)	28 (9.3)	32 (10.7)	24 (8.0)	27 (9.0)
Every few weeks	12 (4.0)	8 (2.7)	10 (3.3)	17 (5.7)	20 (6.7)	17 (5.7)	20 (6.7)	17 (5.7)
Once a month or less	22 (73)	28 (9.3)	27 (9.0)	33 (11.0)	21 (7.0)	33 (11.0)	21 (7.0)	21 (7.0
Never	201 (67.0)	189 (63.0)	185 (61.7)	176 (58.7)	178 (59.3)	155 (51.7)	163 (54.3)	179 (59.7)

Notes. Test for variance across conditions; continuous variables analysed using one-way Anova test, categorical variables analysed using χ^2 test

For peer review only

Statistical Analysis

We used GPower (version 3.1) (29) to estimate effect sizes in the outcome variables as a function of message condition, assuming two-tailed tests, with 80% power and α=0.05. Based on these analyses, a final sample size of 2,400 (600 in each arm) was deemed sufficient power to detect small effects in between-subject analyses of the main effect of condition among adult smokers (f=0.07). In stratified analyses by country, a sample size of 1,200 (300 in each arm) will also ensure sufficient power to detect small effects between conditions (f=0.10).

Analyses were completed in 2020. Randomised controlled trials aim to compare groups of participants that differ only with respect to the intervention, (30) in this case exposure to misinformation. We performed univariate analyses for all study variables. Next, we analysed whether participants across conditions differed in terms of individual characteristics. To address the study aims, we utilised linear regression to predict post-test intentions to quit smoking, intentions to purchase e-cigarettes, and perceived relative harm of e-cigarettes by experimental condition compared with the control condition, adjusting for baseline measures of each outcome respectively. Owing to overdispersion of the second outcome measure, intentions to purchase e-cigarettes, we additionally ran negative binomial regression models. We also ran sensitivity analyses, including country as a covariate (owing to the differences in baseline measurements between the US and the UK; analysis using robust standard errors and bootstrapping – owing to non-normal distribution of residuals. We further conducted stratified analyses to compare the effects of experimental condition on each study outcome among US and UK participants separately. We also tested for interactions between experimental conditions and country (US or UK). Stata version 15.1 was used to conduct all analyses.(31)

For peer review only

RESULTS

Participants were 2,400 adult current smokers recruited between 8-28 November 2019 (see Figure 5: CONSORT diagram). They were aged 18-84 years (mean=47.0, SD=14.58), 46.8% were female, 70.9% of the US participants were White, 16.8% Black or African American and 12.3% were of Other racial background, 90.3% of US participants were non-Hispanic. While 93.3% of the UK cohort were White and 6.7% were from other ethnic backgrounds. Most of the cohort (82.1%) smoked cigarettes every day and 51.6% had ever used e-cigarettes. Table 1 summarises the sample characteristics by experimental condition. We found that randomisation had been achieved and all covariates were distributed evenly across the four study conditions. Three quarters of participants (n=1,804, 75.2%) had not previously searched for information on e-cigarettes. Participants were more likely to report that they had heard that e-cigarettes are more harmful than cigarettes (n=1,297, 54.0%), than hearing that e-cigarettes are harmless (n=662, 27.6%). Over half of the sample (n=1,426, 59.4%) had never used Twitter, with Facebook being the most common social media platform used several times a day (n=1,194, 49.8%).

At baseline, 25.2% of participants (n= 605) placed themselves in the middle of the intention to quit ladder (mean=5.0, SD=3.0), this was similar for both US and UK participants. Over half the participants (n=1,312, 54.7%) said that there was no chance/almost no chance that they would buy e-cigarettes in the next month. The distributions for intentions to buy, were also very similar for US and UK participants. However, there were marked differences between the two populations with respect to perceptions of the relative harm of e-cigarettes: nearly twice as many UK participants said that e-cigarettes are much less harmful than regular cigarettes compared to US participants. Similarly, more than twice as many UK participants said that e-cigarettes are less harmful than regular cigarettes (n=448, 37.3%), compared to US participants (n=222,

For peer review only

18.5%). Conversely, more than three times as many US participants thought that e-cigarettes are much more harmful than regular cigarettes (US: n=217, 18.1%, UK: n=69, 5.8%) and more than twice as many saw them as more harmful (US: n=128, 10.7%, UK: n=62, 5.2%).

We additionally compared the mean and standard deviation (SD) for the outcome measures, both pre- and post-exposure across the four conditions for the US and UK separately (Table 2). We found that pre-exposure intentions to quit and perceptions of the relative harm of e-cigarettes were generally higher and intentions to purchase e-cigarettes were generally lower among US participants.

Tables 3 and 4 summarise the results from the regression analyses. The adjusted analysis includes both the experimental condition as the exposure and the baseline measure of the outcomes. We present the adjusted analysis here. Compared with the control condition, there was no difference in the post-test intention to quit smoking among those who viewed tweets stating that e-cigarettes are as or more harmful than cigarettes, the completely harmless condition or tweets that are uncertain. The results did not change substantially in the stratified analysis (Table 4).

Compared with participants assigned to the control group, there was a statistically significant reduction in post-test intention to purchase e-cigarettes for those exposed to the as or more harmful messages (β= -0.339, 95%CI: -0.487, -0.191, p<0.001). In the stratified analysis, the effect of viewing as or more harmful tweets on reducing intentions to purchase e-cigarettes was observed in both US (β=-0.312, 95%CI: -0.522, -0.073, p=0.011) and UK samples (β= -0.365, 95%CI: -0.551, -0.178, p<0.001). Further, the effect of viewing tweets that e-cigarettes are completely harmless was associated with an increase in intention to purchase e-cigarettes but

For peer review only

Table 2: Outcome measures by experimental condition and country

	US				UK			
**Condition**	1	2	3	4	1	2	3	4
**Outcome measures**								
**Intentions to quit smoking**	n=300	n=300	n=300	n=300	n=300	n=300	n=300	n=300
Pre-exposure: Mean (SD)	5.16 (2.94)	5.25 (3.17)	5.23 (3.00)	5.48 (3.14)	4.72 (2.85)	4.73 (2.86)	4.83 (2.90)	4.78 (3.04)
Post-exposure: Mean (SD)	5.29 (2.96)	5.34 (3.15)	5.46 (3.04)	5.72 (3.20)	4.93 (2.90)	4.80 (2.91)	4.96 (2.89)	4.93 (3.09)
**Intentions to purchase e-cigarettes**	n=300	n=300	n=300	n=300	n=300	n=300	n=300	n=300
Pre-exposure: Mean (SD)	1.33 (2.24)	1.15 (2.08)	1.25 (2.20)	1.29 (2.23)	1.67 (2.37)	1.57 (2.33)	1.88 (2.54)	1.71 (2.47)
Post-exposure: Mean (SD)	0.98 (2.02)	1.30 (2.27)	1.16 (2.17)	1.27 (2.31)	1.21 (2.16)	1.68 (2.56)	1.73 (2.50)	1.79 (2.61)
**Perceptions of relative harms of e-cigarettes**	n=274	n=268	n=274	n=276	n=272	n=270	n=262	n=271
Pre-exposure: Mean (SD)	3.17 (1.03)	3.35 (1.28)	3.20 (1.04)	3.26 (1.10)	2.64 (0.95)	2.67 (0.93)	2.60 (0.90)	2.68 (0.90)
Post-exposure: Mean (SD)	3.45 (1.06)	3.15 (1.12)	3.22 (1.02)	3.22 (1.07)	3.02 (1.00)	2.60 (0.98)	2.60 (0.93)	2.66 (0.92)

For peer review only

Table 3: Regression analysis predicting intention to quit regular cigarettes, intention to purchase an e-cigarette and perceived relative harm of e-cigarettes compared to regular cigarettes (adjusted for baseline measures of outcome)

	Intention to quit smoking regular cigarettes (n=2,400)			Intention to purchase e-cigarette[1] (n=2,400)			Perceived relative harm of e-cigarettes compared to regular cigarettes (n=2,167)		
	β	95% CI	p	β	95% CI	p	β	95% CI	p
Control (referent)									
As or more harmful	-0.031	[-0.152, 0.091]	0.622	-0.339	[-0.487, -0.191]	≤0.001	0.341	[0.273, 0.410]	≤0.001
Completely harmless	-0.120	[-0.241, 0.002]	0.054	0.111	[-0.029, 0.250]	0.120	-0.106	[-0.174, -0.037]	0.003
Uncertainty	-0.017	[-0.139, 0.104]	0.780	-0.106	[-0.247, 0.036]	0.143	-0.018	[-0.051, 0.086]	0.615
Pre-exposure intention to quit	0.945	[0.931, 0.960]	≤0.001	-	-	-	-	-	-
Pre-exposure intention to purchase	-	-	-	0.437	[0.417, 0.458]	≤0.001	-	-	-
Pre-exposure Perceived relative harm of e-cigarettes	-	-	-	-	-	-	0.841	[0.818, 0.864]	≤0.001
		R^2= 0.874			*Pseudo R^2 = 0.2125*<br>*Alpha=0: p≤0.001*			*R^2 =0.704*	

1. For intention to purchase e-cigarettes Negative Binomial Regression was conducted due to a zero-inflated distribution / non-normal distribution

Table 4: Regression analysis predicting intention to quit regular cigarettes, intention to purchase an e-cigarette and perceived relative harm of e-cigarettes compared to regular cigarettes stratified by country of residence status (adjusted for baseline measures of outcome)

	Intention to quit smoking regular cigarettes (US, n=1,200; UK n=1,200)			Intention to purchase e-cigarette[1] (US, n=1,200; UK n=1,200)			Perceived relative harm of e-cigarettes compared to regular cigarettes (US, n=1,092; UK n=1,075)		
	β	95% CI	p	β	95% CI	p	β	95% CI	p
**USA**									
Control (referent)									
As or more harmful	-0.126	[-0.305, 0.054]	0.169	-0.312	[-0.552, -0.073]	0.011	0.296	[0.193, 0.400]	≤0.001
Completely harmless	-0.161	[-0.340, 0.019]	0.079	0.229	[0.002, 0.456]	0.048	-0.154	[-0.258, -0.050]	0.004
Uncertainty	-0.025	[-0.204, 0.155]	0.786	-0.102	[-0.334, 0.130]	0.389	0.036	[-0.067, 0.140]	0.492
Pre-exposure intention to quit	0.940	[0.920, 0.961]	≤0.001	-	-	-	-	-	-
Pre-exposure intention to purchase	-	-	-	0.475	[0.439, 0.510]	≤0.001	-	-	-
Pre-exposure Perceived relative harm of e-cigarettes	-	-	-	-	-	-	0.807	[0.773, 0.841]	≤0.001
		R^2=0.869			*Pseudo R^2 = 0.205*<br>*Alpha=0: p≤0.001*			*R^2 =0.666*	
**UK**									
Control (referent)									
As or more harmful	0.063	[-0.101, 0.228]	0.451	-0.365	[-0.551, -0.178]	≤0.001	0.385	[0.297, 0.474]	≤0.001
Completely harmless	-0.079	[-0.244, 0.085]	0.344	0.034	[-0.141, 0.208]	0.707	-0.053	[-0.142, 0.035]	0.238
Uncertainty	-0.011	[-0.176, 0.154]	0.895	-0.113	[-0.289, 0.062]	0.205	-0.002	[-0.092, 0.087]	0.958
Pre-exposure intention to quit	0.948	[0.928, 0.968]	≤0.001	-	-	-	-	-	-
Pre-exposure intention to purchase	-	-	-	0.406	[0.381, 0.431]	≤0.001	-	-	-
Pre-exposure Perceived relative harm of e-cigarettes	-	-	-	-	-	-	0.875	[0.840, 0.909]	≤0.001

R^2= 0.879	*Pseudo R^2 = 0.217*<br>*Alpha=0: p*≤0.001	*R^2 =0.701*

Notes. Above models controlled for pre-exposure measure of outcome.

1. For intention to purchase e-cigarettes Negative Binomial Regression was conducted due to a zero-inflated distribution / non-normal distribution

For peer review only

only after stratification (β=0.229, 95%CI: 0.002, 0.456, p=0.048) and only among US participants.

Compared to participants assigned to the control messages, participants who viewed the as or more harmful messages were significantly more likely to perceive e-cigarettes as *more* harmful than regular cigarettes (β=0.341, 95%CI: 0.273, 0.410, p<0.001). Participants assigned to the completely harmless messages were significantly more likely to perceive e-cigarettes as *less* harmful than regular cigarettes (β=-0.106, 95%CI: -0.174, -0.037, p=0.003). These effects remained following stratification by country (UK: β=0.385, 95%CI: 0.298, 0.476, p<0.001; US: β= 0.296, 95%CI: 0.193, 0.400, p<0.001). The effect of the completely harmless misinformation on participants perceiving e-cigarettes as less harmful than cigarettes was limited to the US population after stratification (β= -0.154, 95%CI: -0.258, -0.050, p=0.004).

We additionally ran a number of sensitivity analyses owing to differences in baseline measurement between the US and the UK, and non-normality of residuals in the regression analyses. However, there were no substantial differences to report from any of the sensitivity analyses (see Table 5). We additionally tested for interactions between experimental conditions and country (US or UK), but found no evidence of an effect.

For peer review only

Table 5: Sensitivity analyses: adjusted regression analysis predicting intention to quit regular cigarettes, intention to purchase an e-cigarette and perceived relative harm of e-cigarettes compared to regular cigarettes A: includes country as a covariate, B: analysis with robust standard errors and C: analysis with bootstrapping

	Intention to quit smoking regular cigarettes (n=2,400)			Intention to purchase e-cigarette (n=2,400)			Perceived relative harm of e-cigarettes (n=2,167)		
	β	95% CI	p	β	95% CI	p	β	95% CI	p
A									
Control (referent)									
As or more harmful	-0.031	[-0.153, 0.091]	0.620	-0.337	[-0.485, -0.189]	≤0.001	0.341	[0.273, 0.410]	≤0.001
Completely harmless	-0.120	[-0.241, 0.002]	0.054	0.111	[-0.029, 0.250]	0.120	-0.105	[-0.174, -0.037]	0.003
Uncertainty	-0.017	[-0.139, 0.104]	0.779	-0.106	[-0.247, 0.035]	0.142	-0.017	[-0.052, 0.086]	0.628
B									
Control (referent)									
As or more harmful	-0.031	[-0.156, 0.095]	0.633	-0.339	[-0.499, -0.179]	≤0.001	0.341	[0.271, 0.412]	≤0.001
Completely harmless	-0.120	[-0.241, 0.002]	0.054	0.111	[-0.036, 0.258]	0.141	-0.106	[-0.163, -0.048]	≤0.001
Uncertainty	-0.017	[-0.132, 0.097]	0.767	-0.106	[-0.253, 0.042]	0.160	0.018	[-0.044, 0.079]	0.572
C									
Control (referent)									
As or more harmful	-0.031	[-0.147, 0.085]	0.605	-0.339	[-0.493, -0.185]	≤0.001	0.341	[0.280, 0.403]	≤0.001
Completely harmless	-0.120	[-0.237, 0.002]	0.047	0.111	[-0.040, 0.262]	0.151	-0.106	[-0.168, -0.044]	0.001
Uncertainty	-0.017	[-0.151, 0.116]	0.799	-0.106	[-0.239, 0.028]	0.121	0.018	[-0.051, 0.087]	0.617

For peer review only

DISCUSSION

Our results suggest that exposure to misinformation about e-cigarette harms influences adult smokers' decisions to purchase e-cigarettes and their perceived relative harm of e-cigarettes, compared to regular cigarettes. To our knowledge this is the first study to test the effect of brief exposure to misinformation and uncertainty about e-cigarette harms on Twitter on smokers' intentions to quit smoking, intentions to use e-cigarettes and perceptions of relative harm. Both US and UK samples of adult smokers were adversely affected by misinformation about e-cigarettes. We also observed that US smokers who viewed tweets that e-cigs were completely harmless reported lower perceived harms of vaping and higher intentions to purchase e-cigarettes in this study. This effect was absent among UK smokers. This difference between US and UK smokers may be due to the differing policy contexts of the countries. However, further research is needed to assess underlying policy and contextual factors that explain these differences between countries in the effects of e-cigarette misinformation.

These findings are important because they show that after brief exposure to tweets that e-cigarettes are as or more harmful than smoking, current smokers may be deterred from using e-cigarettes (measured with intention to purchase e-cigarettes) as a harm reduction strategy. They are also more likely to wrongly believe that e-cigarettes are more harmful than regular cigarettes. However, more research is needed to assess whether misinformation exposure about e-cigarette harms will negatively influence smokers' behaviours to reduce harms from using combusted cigarettes by opting for less harmful forms of nicotine delivery using e-cigarettes. . There is consensus that debunking or correcting exposure to misinformation is extremely challenging, common techniques have even been found to further engrain misinformation.(15,32) Reducing exposure to misinformation has its own challenges, as misinformation on social media spreads

For peer review only

more pervasively than accurate information and the spread is due to mostly human actions, rather than automated bots.(33) In addition, it is often hard to categorise content as misinformation, especially when the evidence around a given health topic is inconclusive, or the way the information is communicated is unclear. This creates challenges in both harnessing algorithms to alert users to misinformation and also communicating ways to spot misinformation. These points, combined with our findings, have the potential to undermine the efforts of the public health community to reduce harm among current smokers. However, innovative health communication approaches need to be developed and tested to both reduce exposure to and counter misinformation using effective harm reduction and health promotion strategies. Strategies are already being employed by social media platforms to address the problem of misinformation, for example, downranking content and removing or blocking users with content identified as misinformation. While it may be difficult to keep up with and identify health misinformation as such, it is possible to warn smokers of the problem of misinformation and encourage them to seek out their health information from official sources. Health care providers' should be aware that their patients may have seen misinformation on social media and hold incorrect beliefs about e-cigarettes. They should always correct these and consider the ways they can help their patients to identify accurate health information. Finally, governments and policy makers should make sure all social media searches for e-cigarettes are flagged with official health guidance. They should also regulate all forms of misinformation on social media and improve people's awareness and ability to find accurate information.

There are several limitations of the study, first, we excluded visual content from the exposures to ensure that the format of tweets was consistent across conditions and participants were focused on the content of the tweets. However, prior studies indicate that visual cues within e-cigarette

For peer review only

advertisements are associated with perceptions about and decisions to use e-cigarettes.(8,34) Second, health misinformation is spread in different ways. We used Twitter data because it is free and publicly available and because of the documented prevalence of health misinformation on Twitter.(17,18) However, over half of participants (59%) indicated they did not use Twitter meaning they may not be familiar with viewing or engaging with tweets. To address this, we included definitions of each of these engagement behaviours, prior to responding to questions on the likelihood of replying, retweeting, liking, or sharing each message. Further, our findings are still useful because intentions are strong predictors of behaviour, as shown by Ajzen's Theory of Planned Behavior.(35) Misinformation is ubiquitous - Allcott and colleagues found a total of 672 sites producing false stories or unique fake news sites.(36) Stories from these sites are shared on Facebook, Twitter and cross-posted on other social media platforms. Therefore, while this sample may not be exposed to misinformation on Twitter in real life, they are likely exposed via different channels. Third, there is the issue of the reliability of self-reported smoking compared to biochemical verification of smoking status. However, given that we used an online self-administered survey, it is unlikely to have a big impact on participants' answers. Further, it's been shown that self-reported smoking prevalence, checked by biochemical verification, was underestimated by only 0.6% in the US and 2.8% in the UK.(37) Fourth, our study sample was not fully representative of the populations they were drawn from. For example, White people make up 86% of the UK population, but represented 93.3% of the UK sample in this study, which may mean our findings are not generalisable. Fifth, previous research on health misinformation on social media identified important factors that might play a role in the mechanism of action of misinformation. Among those factors are the type of content, the source of the message, the sender's authoritativeness, the argument length, the novelty, timing,

For peer review only

repetition and hashtags. We were not able to examine the impact of these message features in detail. Future research is needed to determine the effects of varying these features on smokers' processing of misinformation about e-cigarettes. (33,38,39) Finally, there was an outbreak of e-cigarette or vaping product use-associated lung injuries that were first identified in August 2019 in the US and subsequently traced to products containing Tetrahydrocannabinol (THC) from the illicit market. This outbreak, in combination with the different contexts of the two countries, may have influenced participants' views on e-cigarettes during the time of the study data collection. However, because of the experimental design to randomly assign participants into conditions, we do not anticipate that this would have biased our findings systematically.

Future research should focus on identifying the factors that make misinformation effective and how it is perceived by exposed individuals. Conducting research using different social media platforms, study designs and analytical tools, and focusing on analysing the message or communication factors are all important. According to our study, Facebook was overwhelmingly the social media platform used by these participants. It would therefore be interesting to replicate this research using Facebook. Second, there is a need to explore the role of cognitive factors, beliefs, past experiences and other individual level factors in the effects of misinformation. For instance, based on the theory of bias assimilation stating that people gravitate to information they have previously heard, future research should test whether the observed results could be explained by the fact that many individuals were previously exposed to misinformation. Third, it is important to refine and further develop a reliable algorithm that could distinguish between accurate and misinformation about e-cigarettes. With the amount of information that is currently generated by users on different social media platforms, an automated approach of identifying misinformation could be most cost-effective and timely. Nevertheless, any algorithms, evident

For peer review only

from our prior work,(24,25) cannot achieve 100% accuracy, leading to misclassification errors and require constant refinement and evaluation as new types of misinformation emerge. Fourth, we were not able to examine the impact of specific features of the tweets, for example the source of the message or the sender's authoritativeness. Future research is needed to determine the effects of varying these features on smokers' processing of misinformation about e-cigarettes. Our exposure was only brief therefore, future research to evaluate the effect of longer or repeated exposures to misinformation would also be useful, to assess the effects on e-cigarette use intentions and subsequent vaping or smoking behaviours. Finally, future research could extend our analysis to include behaviours as well as intentions.

For peer review only

CONCLUSIONS

US and UK adult current smokers may be deterred from considering using e-cigarettes after brief exposure to tweets that e-cigarettes are as or more harmful than smoking. Conversely, US adult current smokers may be encouraged to use e-cigarettes and view them as less harmful than regular cigarettes, after exposure to tweets that e-cigarettes are completely harmless. These findings suggest that misinformation about e-cigarette harms may influence adult smokers' decisions to consider using e-cigarettes.

For peer review only

Acknowledgements: We would like to thank Cancer Research UK who funded this research.

Funding: Research reported in this publication was supported by a Cancer Policy Research Centre Innovation grant (C60153/A28664). Dr Wright is funded by a Cancer Research UK Population Research Postdoctoral Fellowship (C60153/A23895). Dr Dahne is supported by the National Institute on Drug Abuse, K23 DA045766. We would also like to thank the National Cancer Institute (NCI) for their support during the sandpit event where this research idea came about.

Competing interests: We declare no competing interests.

Contributors: CW, AT, OE, JD and JB all contributed to the original research idea. JB and YZ did the machine learning and JB and PW annotated the tweets. All authors contributed to the design of the survey instrument. CW and PW did the statistical analysis with input from all the authors. All authors contributed to the drafting and editing of the paper.

Data sharing statement: Following the completion of the research outlined in our research proposal and subsequent publications, instructions for accessing the data will be made available. Requests for data will be fielded by Dr Caroline Wright and shared with other researchers, provided a satisfactory data-sharing agreement has been completed by the requesting researcher. The data-sharing agreement will impose appropriate limitations on the secondary use of the data, with reference to the 'Samples and Data for Cancer Research: Template for Access Policy Development' document. We also plan to publish the data from the study once all research outlined in our research proposal has been submitted for publication.

Data availability: Unpublished data, the details of which can be found here: https://doi.org/10.1186/ISRCTN16082420 are available upon reasonable request.

Ethical approval: The University of Bristol's Institutional Review Board, Faculty of Health Sciences Research Ethics Committee (FREC) approved this study. Ref: 80323 The tweets used in this research are all in the public domain and participants could therefore have been exposed to this misinformation at any time. We further provided participants with a debrief of accurate information about e-cigarettes compared to regular cigarette harms as well as information about smoking cessation services.

Patient and Public Involvement: Patients were not involved in the design of this research.

Figure captions:

For peer review only

Figure 1. Condition 1 E-cigarettes are as or more harmful

Figure 2. Condition 2 E-cigarettes are completely harmless

Figure 3. Condition 3 Messages expressing uncertainty about e-cigarettes

Figure 4. Condition 4 Messages about Physical Activity

Figure 5. CONSORT Flow Diagram

For peer review only

REFERENCES

1. NASEM. Public Health Consequences of E-Cigarettes. Eaton DL, Kwan LY, Stratton K, editors. Washington (DC); 2018.
2. McNeill A, Brose LS, Calder R BL& RD. Evidence review of ecigarettes and heated tobacco products 2018. A report commissioned by Public Health England. London; 2018.
3. Smith CA, Shahab L, McNeill A, Jackson SE, Brown J, Brose L. Harm perceptions of e-cigarettes among smokers with and without mental health conditions in England: A cross-sectional population survey. Nicotine Tob Res Off J Soc Res Nicotine Tob. 2020 Jan;
4. Nyman AL, Huang J, Weaver SR, Eriksen MP. Perceived Comparative Harm of Cigarettes and Electronic Nicotine Delivery Systems. JAMA Netw Open [Internet]. 2019 Nov 20;2(11):e1915680–e1915680. Available from: https://doi.org/10.1001/jamanetworkopen.2019.15680
5. McNeill, A., Brose, L.S., Calder, R., Bauld, L., and Robson D. Vaping in England: an evidence update including mental health and pregnancy, March 2020: a report commissioned by Public Health England. London; 2020.
6. Persoskie A, O'Brien EK, Poonai K. Perceived relative harm of using e-cigarettes predicts future product switching among US adult cigarette and e-cigarette dual users. Addiction. 2019 Dec;114(12):2197–205.
7. Southwell BG, Niederdeppe J, Cappella JN, Gaysynsky A, Kelley DE, Oh A, et al. Misinformation as a Misunderstood Challenge to Public Health. Am J Prev Med. 2019 Aug;57(2):282–5.
8. Wang Y, McKee M, Torbica A, Stuckler D. Systematic Literature Review on the Spread of Health-related Misinformation on Social Media. Soc Sci Med [Internet]. 2019;240:112552. Available from: http://www.sciencedirect.com/science/article/pii/S0277953619305465
9. Brashers DE. Communication and Uncertainty Management. J Commun [Internet]. 2001 Sep 1;51(3):477–97. Available from: https://doi.org/10.1111/j.1460-2466.2001.tb02892.x
10. ONS. Internet access – households and individuals, Great Britain: 2019. London; 2019.
11. Finney Rutten LJ, Blake KD, Greenberg-Worisek AJ, Allen S V, Moser RP, Hesse BW. Online Health Information Seeking Among US Adults: Measuring Progress Toward a Healthy People 2020 Objective. Public Health Rep. 2019;134(6):617–25.
12. Chou W-YS, Oh A, Klein WMP. Addressing Health-Related Misinformation on Social Media. JAMA. 2018 Dec;320(23):2417–8.
13. Chong E, Xie B. The Use of Theory in Social Studies of Web 2.0. 2011. 1–10 p.
14. Walther JB, Jang J. Communication Processes in Participatory Websites. J Comput Commun [Internet]. 2012;18(1):2–15. Available from: https://doi.org/10.1111/j.1083-6101.2012.01592.x
15. Lewandowsky S, Ecker UKH, Seifert CM, Schwarz N, Cook J. Misinformation and Its Correction: Continued Influence and Successful Debiasing. Psychol Sci Public Interes [Internet]. 2012 Sep 17;13(3):106–31. Available from: https://doi.org/10.1177/1529100612451018
16. Chou WS, Prestin A, Lyons C, Wen K. Web 2.0 for Health Promotion: Reviewing the Current Evidence. 2013;103(1):e9–18. Available from: https://ajph.aphapublications.org/doi/abs/10.2105/AJPH.2012.301071
17. Allem J-P, Ferrara E, Uppu SP, Cruz TB, Unger JB. E-Cigarette Surveillance With Social

For peer review only

Media Data: Social Bots, Emerging Topics, and Trends. JMIR Public Heal Surveill [Internet]. 2017;3(4):e98. Available from: http://publichealth.jmir.org/2017/4/e98/
18. Jamison AM, Broniatowski DA, Quinn SC. Malicious Actors on Twitter: A Guide for Public Health Researchers. Am J Public Health. 2019 May;109(5):688–92.
19. Pew Research Center. Social Media Use in 2019. 2019.
20. We Are Social. Digital 2020: The United Kingdom [Internet]. London; 2020. Available from: https://wearesocial.com/uk/digital-2020-uk
21. Fairchild AL, Bayer R, Lee JS. The E-Cigarette Debate: What Counts as Evidence? Am J Public Health [Internet]. 2019 May 16;109(7):1000–6. Available from: https://doi.org/10.2105/AJPH.2019.305107
22. Amin S, Dunn AG, Laranjo L. Social Influence in the Uptake and Use of Electronic Cigarettes: A Systematic Review. Am J Prev Med. 2020 Jan;58(1):129–41.
23. Wright CL, Williams P, Elizarova O, Dahne J, Jiang B, Zhao Y, et al. Does viewing false messages about e-cigarette harms on Twitter change current smokers' perceptions of e-cigarettes in the US and the UK? A randomised controlled experiment [Internet]. ISRCTN. 2020. Available from: http://www.isrctn.com/ISRCTN16082420
24. Guo B, Ding Y, Yao L, Liang Y, Yu Z. The Future of Misinformation Detection: New Perspectives and Trends. 2019.
25. Thota, Aswini; Tilak, Priyanka; Ahluwalia, Simrat; and Lohia N. Fake News Detection: A Deep Learning Approach. SMU Data Sci Rev [Internet]. 2018;1(3). Available from: https://scholar.smu.edu/datasciencereview/vol1/iss3/10
26. Biener, L., & Abrams DB. The Contemplation Ladder: Validation of a measure of readiness to consider smoking cessation. Heal Psychol [Internet]. 1991;10(5):360–5. Available from: https://doi.org/10.1037/0278-6133.10.5.360
27. Juster FT. Consumer Buying Intentions and Purchase Probability: An Experiment in Survey Design. J Am Stat Assoc [Internet]. 1966 Sep 1;61(315):658–96. Available from: https://amstat.tandfonline.com/doi/abs/10.1080/01621459.1966.10480897
28. NIH. HINTS 5 Cycle 2 Survey Materials [Internet]. 2018. Available from: file://ads.bris.ac.uk/filestore/BRMS/Studies/E-Mute/Papers/Main_analysis_paper/HINTS5_Cycle2_Annotated_Instrument_English.pdf
29. Erdfelder E, Faul F, Buchner A. GPOWER: A general power analysis program. Behav Res Methods, Instruments, Comput [Internet]. 1996;28(1):1–11. Available from: https://doi.org/10.3758/BF03203630
30. Moher D, Hopewell S, Schulz KF, Montori V, Gøtzsche PC, Devereaux PJ, et al. CONSORT 2010 Explanation and Elaboration: updated guidelines for reporting parallel group randomised trials. J Clin Epidemiol [Internet]. 2010;63(8):e1–37. Available from: http://www.sciencedirect.com/science/article/pii/S0895435610001034
31. StataCorp. Stata Statistical Software: Release 15. Texas: StataCorp LLC.; 2017.
32. Cappella JN, Maloney E, Ophir Y, Brennan E. Interventions to Correct Misinformation About Tobacco Products. Tob Regul Sci [Internet]. 2015/07/01. 2015 Jul;1(2):186–97. Available from: https://pubmed.ncbi.nlm.nih.gov/27135046
33. Vosoughi S, Roy D, Aral S. The spread of true and false news online. Science (80-) [Internet]. 2018 Mar 9;359(6380):1146 LP – 1151. Available from: http://science.sciencemag.org/content/359/6380/1146.abstract
34. King AC, Smith LJ, Fridberg DJ, Matthews AK, McNamara PJ, Cao D. Exposure to electronic nicotine delivery systems (ENDS) visual imagery increases smoking urge and

For peer review only

desire. Psychol Addict Behav [Internet]. 2015/11/30. 2016 Feb;30(1):106–12. Available from: https://pubmed.ncbi.nlm.nih.gov/26618797
35. Ajzen I. The theory of planned behavior. Organ Behav Hum Decis Process [Internet]. 1991;50(2):179–211. Available from: https://www.sciencedirect.com/science/article/pii/074959789190020T
36. Allcott H, Gentzkow M, Yu C. Trends in the diffusion of misinformation on social media. Res Polit [Internet]. 2019 Apr 1;6(2):2053168019848554. Available from: https://doi.org/10.1177/2053168019848554
37. West R, Zatonski W, Przewozniak K, Jarvis MJ. Can We Trust National Smoking Prevalence Figures? Discrepancies Between Biochemically Assessed and Self-Reported Smoking Rates in Three Countries. Cancer Epidemiol Biomarkers & Prev [Internet]. 2007 Apr 1;16(4):820 LP – 822. Available from: http://cebp.aacrjournals.org/content/16/4/820.abstract
38. Zhou J, Liu F, Zhou H. Understanding health food messages on Twitter for health literacy promotion. Perspect Public Health [Internet]. 2018 Mar 7;138(3):173–9. Available from: https://doi.org/10.1177/1757913918760359
39. Wood MJ. Propagating and Debunking Conspiracy Theories on Twitter During the 2015-2016 Zika Virus Outbreak. Cyberpsychol Behav Soc Netw [Internet]. 2018/07/18. 2018 Aug;21(8):485–90. Available from: https://pubmed.ncbi.nlm.nih.gov/30020821

For peer review only

Figure 1. Condition 1: E-cigarettes are as or more harmful

How about let's focus on the matter at hand which is Big Tobacco duping an entire generation - again - on our watch. Let's get the word out and educate young and old, vaping and E-cigarettes kill. @RAI_News

Smoking takes decades to cause cancer. Vaping, it seems, takes only a few years. The evidence is clear enough for US to ban flavoured vaping today, yep, today. The rest will follow as facts emerge, I imagine. It's pretty disgusting anyway -try it.

Vaping is still pretty much just as dangerous as cigs bc everything goes DIRECTLY into your lungs. Oh I forgot to mention the flavoring chemicals in vapes can also cause cancer. Seriously, look up actual medical research please.

#JUUL should be banned immediately. Anyone thinking vaping cigarettes is better than smoking is being conned. Vaping chemicals into your lungs will kill you. Altria is a murderer. Flat out mass murder. $MO

For peer review only - http://bmjopen.bmj.com/site/about/guidelines.xhtml

Figure 2. Condition 2: E-cigarettes are completely harmless

WOW. You're a doctor and you are spreading this fearmonger propaganda? What happened to your oath to do no harm?

There are ZERO proven harms in the 15 years vaping has existed when used in the suggested parameters.

I highly suggest you educate yourself on all the facts.. 1/2

I'm an asthmatic lol. I know the science behind vaping. It's completely safe. Big tobacco scares ppl. Like thruth dot .org... big tobacco supports them. It's crazy.

Oh, it's not only safer, they are SAFE - or, you know of any harm by vaping tho ~15y on the market and ~50.000.000 users world-wide? - and ~8.000 flavours! No, didn't think so bcos NONE so far - NONE - that'ts how SAFE vaping is - did say vaping. Any objections to that?

I don't worry about the ingreds of ejuice for #vaping, they are harmless, but I do wonder about the artificial breathing, the regular deep puffing. Do trumpet players get a breathing disorder? My puffing #ecigs is kind of like that.

Figure 3. Condition 3: Messages expressing uncertainty about e-cigarettes

We Still Don't Know How Safe #Vaping Is - it's time to get more information about the risks of #ecigarettes: @nytimes editorial

And people are like "but it's not that bad because it's not smoke" ok but nicotine is harmful with or without smoke and there is very limited research done on e-cigs so the FDA doesn't know how harmful they actually are to the extent that we know that cigarettes are harmful

This whole anti-vaping schtick is cooked up by drug regulation & enforcement to make sure the MONEY keeps flowing to their coffers.

I have yet to see a single credible piece of evidence showing that vaping causes real harm.

(As in more harm than drinking too much coffee.)

Is San Francisco's vaping ban backed by science?: San Francisco has decided to ban the sale of e-cigarettes in 2020, hoping to curb a surge in vaping among adolescents. But is the policy backed up by the available evidence? – How harmful is vaping? –...

Figure 4. Condition 4: Messages about Physical Activity

Today reinforces my passion to push the need to exercise for not only the physical benefits. Get out and do something active for your mental health. Go for a walk and clear your mind. Find someone to join you & talk to them. My prayers go out to all today

---

Adults (18+) need 150 minutes per week of moderate-intensity physical activity to improve and maintain health.

Let's #BeActive!

It's #WorldMentalHealthDay and we know sport and physical activity can have a powerful and positive effect on our mental wellbeing. That's why we invest in projects that are changing lives.

Physical activity & Exercise can have immediate and long-term health benefits. Most importantly, regular activity can improve our quality of life.

For peer review only - http://bmjopen.bmj.com/site/about/guidelines.xhtml

Figure 5. CONSORT Flow Diagram

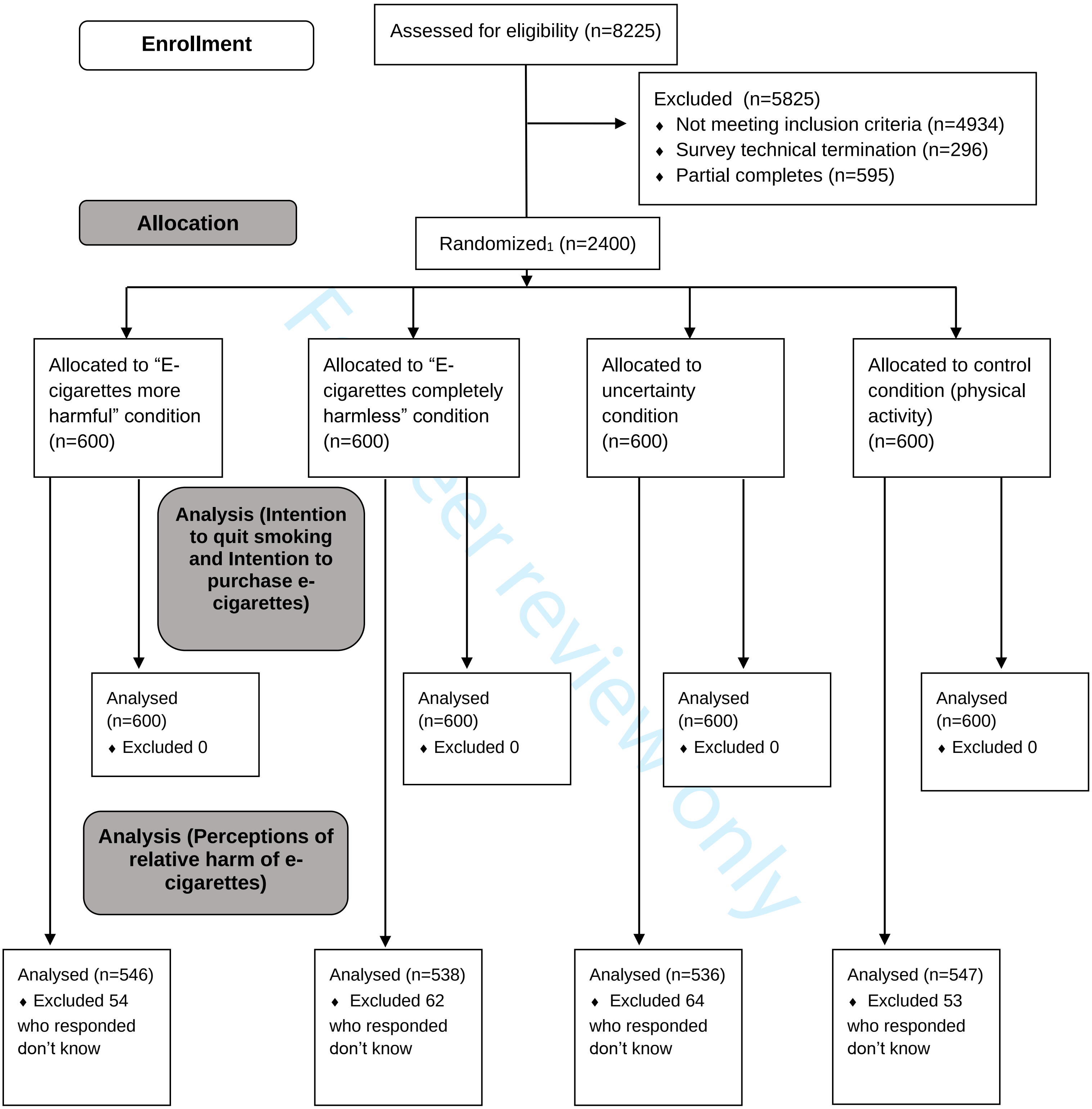

1. *Survey recruitment used a least-fill approach; as a respondent came in, they were assigned to the exposure with the lowest complete count.*

For peer review only

Supplementary material 1: additional questions about tweets

Questions asked after each tweet

We are interested in learning whether you would reply, retweet, like, and share this message, whether you use Twitter regularly or not. A reply is a response to another person's Tweet, a Retweet is a re-posting of a Tweet, Likes are used to show appreciation for a Tweet, and you can Share a tweet via direct message, text message, or email.

Please rate how much you disagree or agree with the following statements about the message you just saw.

a. PE1: This message is worth remembering.
b. PE2: This message grabbed my attention.
c. PE3: This message is powerful.
d. PE4: This message is informative.
e. PE5: This message is meaningful to me.
f. PE6: This message is convincing.

1. Strongly disagree (1)
2. Disagree (2)
3. Neither disagree nor agree (3)
4. Agree (4)
5. Strongly agree (5)

Intentions of replyiong/retweeting/liking/sharing Tweets

Are you likely to Reply to this message?
1. Yes
2. No

Are you likely Retweet this message?
1. Yes
2. No

Are you likely to Like this message?
1. Yes
2. No

Are you likely to Share this message?
1. Yes
2. No

Emotional responses

Please mark an answer for each question in the table below. When thinking about e-cigarettes, does the message you just saw make you feel…

a. Scared
b. Hopeful
c. Worried
d. Happy

For peer review only

e. Angry
f. Relieved

1. Not at all
2. A little
3. Some
4. A lot
5. Completely

For peer review only

For peer review only

Supplementary material 2: experimental conditions

Experimental condition	Exposure 1	Exposure 2	Exposure 3	Exposure 4
1: E-cigarettes are as or more harmful than regular cigarettes	Let's focus on the matter at hand which is big tobacco duping an entire generation – again – on our watch. Let's get the word out and educate young people and old, vaping and e-cigarettes kill.	Smoking takes decades to cause cancer. Vaping, it seems, takes only a few years. The evidence is clear enough for US to ban flavoured vaping today, yep, today. The rest will follow as facts emerge, I imagine. It's pretty disgusting anyway – try it.	Vaping is still pretty much as dangerous as cigs because everything goes directly into your lungs. Oh I forgot to mention the flavouring chemicals in vapes can also cause cancer. Seriously, look up actual medical research please.	Juul should be banned immediately. Anyone thinking vaping cigarettes is better than smoking is being conned. Vaping chemicals into your lungs will kill you. Altria is a murderer. Flat out mass murder.
2: E-cigarettes are completely harmless	Wow. You're a doctor and you are spreading this fearmonger propaganda? What happened to your oath do no harm? There are zero proven harms in the 15 years vaping has existed when used in the suggested parameters. I highly suggest you educate yourself on all the facts.	I'm an asthmatic lol. I know the science behind vaping. It's completely safe. Big tobacco scares ppl. Like truth dot .org…big tobacco supports them. It's crazy.	Oh, it's not only safer, they are safe – or, you know of any harm by vaping though ~15y on the market and ~50.000.000 users world-wide? – and ~8.000 flavours! No, didn't think so because none. That's how safe vaping is – did say vaping. Any objections to that?	I don't worry about the ingredients of e-juice for vaping, they are harmless, but I do wonder about the artificial breathing, the regular deep puffing. Do trumpet players get a breathing disorder? My puffing e-cigs is kind of like that.
3: Messages expressing uncertainty about e-cigarettes	We still don't know how safe vaping is – it's time to get more information about the risks of e-cigarettes.	And people are like "but it's not that bad because it's not smoke" ok but nicotine is harmful with or without smoke and there is very limited research done on e-cigs so the FDA doesn't know how harmful they actually are to the extent that	This whole anti-vaping schtick is cooked up by drug regulation & enforcement to make sure the money keeps flowing to their coffers. I have yet to see a single credible piece of evidence that vaping causes real harm.	Is San Francisco's vaping ban backed by science? San Francisco has decided to ban the sale of e-cigarettes in 2020, hoping to curb a surge in vaping among adolescents. But is the policy backed up by the available

		we know cigarettes are harmful.	(As in more harm than drinking too much coffee.)	evidence? How harmful is vaping?
4: Messages about physical activity (control condition)	Today reinforces my passion to push the need to exercise for not only the physical benefits. Get out and do something active for your mental health. Go for a walk and clear your mind. Find someone to join you and talk to them. My prayers go out to all today.	Adults (those aged 18 or older) need 150 minutes per week of moderate intensity physical activity to improve and maintain health.	It's world mental health day and we know sport and physical activity can have powerful and positive effect on our wellbeing. That's why we invest in projects that are changing lives.	Physical activity and exercise can have immediate and long-term health benefits. Most importantly, regular activity can improve our quality of life.

For peer review only

CONSORT 2010 checklist of information to include when reporting a randomised trial*

Section/Topic	Item No	Checklist item	Reported on page No
**Title and abstract**			
	1a	Identification as a randomised trial in the title	1
	1b	Structured summary of trial design, methods, results, and conclusions (for specific guidance see CONSORT for abstracts)	2
**Introduction**			
Background and objectives	2a	Scientific background and explanation of rationale	4
	2b	Specific objectives or hypotheses	5-6
**Methods**			
Trial design	3a	Description of trial design (such as parallel, factorial) including allocation ratio	7-8
	3b	Important changes to methods after trial commencement (such as eligibility criteria), with reasons	N/A
Participants	4a	Eligibility criteria for participants	7
	4b	Settings and locations where the data were collected	7
Interventions	5	The interventions for each group with sufficient details to allow replication, including how and when they were actually administered	8
Outcomes	6a	Completely defined pre-specified primary and secondary outcome measures, including how and when they were assessed	8-9
	6b	Any changes to trial outcomes after the trial commenced, with reasons	N/A
Sample size	7a	How sample size was determined	11
	7b	When applicable, explanation of any interim analyses and stopping guidelines	N/A
Randomisation:			
Sequence generation	8a	Method used to generate the random allocation sequence	7
	8b	Type of randomisation; details of any restriction (such as blocking and block size)	7
Allocation concealment mechanism	9	Mechanism used to implement the random allocation sequence (such as sequentially numbered containers), describing any steps taken to conceal the sequence until interventions were assigned	7
Implementation	10	Who generated the random allocation sequence, who enrolled participants, and who assigned participants to interventions	7
Blinding	11a	If done, who was blinded after assignment to interventions (for example, participants, care providers, those	N/A

For peer review only

For peer review only - http://bmjopen.bmj.com/site/about/guidelines.xhtml

		assessing outcomes) and how	
	11b	If relevant, description of the similarity of interventions	N/A
Statistical methods	12a	Statistical methods used to compare groups for primary and secondary outcomes	11
	12b	Methods for additional analyses, such as subgroup analyses and adjusted analyses	11
**Results**			
Participant flow (a diagram is strongly recommended)	13a	For each group, the numbers of participants who were randomly assigned, received intended treatment, and were analysed for the primary outcome	Figure 5
	13b	For each group, losses and exclusions after randomisation, together with reasons	Figure 5
Recruitment	14a	Dates defining the periods of recruitment and follow-up	12
	14b	Why the trial ended or was stopped	N/A
Baseline data	15	A table showing baseline demographic and clinical characteristics for each group	10
Numbers analysed	16	For each group, number of participants (denominator) included in each analysis and whether the analysis was by original assigned groups	12
Outcomes and estimation	17a	For each primary and secondary outcome, results for each group, and the estimated effect size and its precision (such as 95% confidence interval)	Table 3 p.15
	17b	For binary outcomes, presentation of both absolute and relative effect sizes is recommended	N/A
Ancillary analyses	18	Results of any other analyses performed, including subgroup analyses and adjusted analyses, distinguishing pre-specified from exploratory	Table 4 p.16
Harms	19	All important harms or unintended effects in each group (for specific guidance see CONSORT for harms)	N/A
**Discussion**			
Limitations	20	Trial limitations, addressing sources of potential bias, imprecision, and, if relevant, multiplicity of analyses	20-21
Generalisability	21	Generalisability (external validity, applicability) of the trial findings	21
Interpretation	22	Interpretation consistent with results, balancing benefits and harms, and considering other relevant evidence	19-22
**Other information**			
Registration	23	Registration number and name of trial registry	26
Protocol	24	Where the full trial protocol can be accessed, if available	26
Funding	25	Sources of funding and other support (such as supply of drugs), role of funders	24

*We strongly recommend reading this statement in conjunction with the CONSORT 2010 Explanation and Elaboration for important clarifications on all the items. If relevant, we also recommend reading CONSORT extensions for cluster randomised trials, non-inferiority and equivalence trials, non-pharmacological treatments, herbal interventions, and pragmatic trials. Additional extensions are forthcoming: for those and for up to date references relevant to this checklist, see www.consort-statement.org.

For peer review only - http://bmjopen.bmj.com/site/about/guidelines.xhtml
